# Change in skeletal muscle mass during systemic cancer treatment: a systematic review and meta-analysis

**DOI:** 10.2340/1651-226X.2026.45726

**Published:** 2026-05-27

**Authors:** Lukas Svendsen, Sandra Jensen, Stine Hansen, Victor Sørensen, Christoffer Johansen, Charlotte Suetta, Helle Pappot, Casper Simonsen, Lars Hermann Tang, Susanne Oksbjerg Dalton, Gunn Ammitzbøll, Bolette Skjødt Rafn

**Affiliations:** a Department of Clinical Oncology & Palliative Care, Zealand University Hospital, Naestved, Denmark; b Danish Research Center for Equality in Cancer (COMPAS), Naestved, Denmark; c Cancer Survivorship, Danish Cancer Institute, Copenhagen, Denmark; d Department of Oncology, Danish Cancer Society National Cancer Survivorship and Late Effects Research Center (CASTLE), Rigshospitalet, Copenhagen, Denmark; e Mental Health Center Glostrup, Centre for Applied Research in Mental Health Care (CARMEN), University of Copenhagen, Copenhagen, Denmark; f Institute of Clinical Medicine, Faculty of Health, University of Copenhagen, Copenhagen, Denmark; g Geriatric and Palliative Department, Copenhagen University Hospital, Bispebjerg and Frederiksberg Copenhagen, Denmark; h Department of Oncology, Rigshospitalet, University Hospital of Copenhagen, Copenhagen, Denmark; i Centre for Physical Activity Research, Rigshospitalet, Copenhagen, Denmark; j The research and implementation unit PROgrez, Department of Physiotherapy and Occupational Therapy, Naestved-Slagelse-Ringsted Hospitals & The Department of Regional Health Research, University of Southern Denmark, Odense, Denmark

**Keywords:** Sarcopenia, skeletal muscle mass, neoplasms, drug therapy, meta-analysis

## Abstract

**Background and purpose:**

Loss of skeletal muscle mass (SMM) is common during systemic cancer treatment, but the magnitude and variability across cancer and treatment types remain uncertain. We aimed to describe changes in SMM during systemic cancer treatment supported by pooled quantitative estimates.

**Patients/material and methods:**

We systematically searched PubMed, Embase, and Web of Science until April 2025 for longitudinal studies reporting SMM during chemotherapy and/or immunotherapy (± targeted therapy) in patients with cancer (PROSPERO CRD42022308388). Standardized mean changes (SMC) were pooled in random-effects meta-analyses using the restricted maximum-likelihood estimator with Hartung–Knapp adjustment. Heterogeneity was assessed using *I*^2^. Risk of bias was assessed with the NIH Quality Assessment Tool for Observational Cohort and Cross-Sectional Studies.

**Results:**

Seventy-eight studies (*n* = 10,502; 52% male; median age 64 years [interquartile range, IQR: 34–77]) were included. Meta-analysis across cancers showed an association between systemic cancer treatment and decline in SMM (59 studies; *n* = 6,373; SMC = −0.24, 95% confidence interval [CI]: −0.29 to −0.20; *I*^2^ = 92%), corresponding to −5% over a median interval of 90 (IQR: 71–129) days among studies (62%) reporting assessment intervals. Declines were most pronounced during chemotherapy (± targeted therapy).

**Interpretation:**

Declines in SMM are frequently observed during systemic cancer treatment, particularly during chemotherapy (± targeted therapy), although effect sizes were generally small per Cohen’s thresholds. However, substantial heterogeneity limits interpretation of a single pooled estimate. Prospective studies with standardized methods are needed to clarify trajectories, mechanisms and clinical implications of SMM loss.

## Graphical abstract

**Figure:**
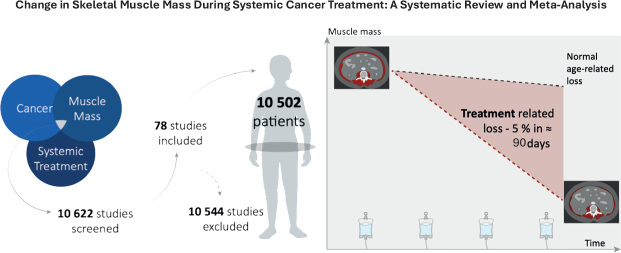

## Introduction

With increasing cancer survival, clinical priorities have shifted from tumor control alone toward also preserving physical function and quality of life during treatment and survivorship [1]. Across cancer types, skeletal muscle mass (SMM) has emerged as a key determinant of these outcomes and a potent factor influencing treatment tolerance and prognosis [2, 3].

Low SMM, often termed sarcopenia [4], is present in approximately one-third of patients with cancer [5, 6] and has consistently demonstrated clinical relevance. Low SMM at time of diagnosis is associated with shorter overall survival [7–10], shorter progression-free survival [11], higher treatment-related toxicity [12], postoperative complications [13], and reduced quality of life and depression [14]. Collectively, these findings demonstrate that low SMM is an important prognostic biomarker that could be incorporated into clinical evaluations and research [15]. However, while the baseline SMM could be clinically useful, particularly in settings where longitudinal imaging is unavailable, emerging evidence shows that changes in SMM during systemic therapy offer additional and often stronger prognostic information across cancer types [16–19]. In a multicohort study including patients with advanced non-small cell lung cancer (*n* = 1,791), less SMM decline was associated with a 26–54% lower mortality risk (Hazard Ratio [HR]: 0.46–0.74) [20]. Similarly, SMM loss during treatment was associated with poorer overall survival as in colorectal (*n* = 67; ≥ 9% SMM loss, HR: 4.47, 95% confidence interval [CI]: 2.21–9.05) [21], biliary tract (*n* = 524; SMM loss; HR: 2.58, 95% CI: 1.86–3.58) [22] and pancreatic cancer (*n* = 127; ≥ 7.9% SMM loss; HR: 4.02, 95% CI: 1.87–8.97) [23]. Collectively, these findings suggest that while baseline SMM is informative, the trajectory of SMM during treatment captures prognostic information that is not evident from a single time point.

Despite this, existing studies are heterogeneous with respect to cancer populations, treatment regimens, measurement methods, and timing of assessments, which complicates direct comparison and limits the ability to derive a single, generalizable estimate of treatment-associated SMM change. The body of literature describing treatment-related SMM loss therefore remains insufficiently characterized in terms of its magnitude, timing, and variability across clinical contexts.

This study aimed to systematically describe changes in SMM during systemic cancer treatment supported by quantitative pooled estimates and to explore if changes vary across cancer types and treatment modalities by conducting a systematic review and meta-analysis of SMM changes during chemotherapy and/or immunotherapy (± targeted therapy).

## Material and methods

This systematic review and meta-analysis was reported according to the Cochrane Handbook [24] and to the Preferred Reporting Items for Systematic Reviews and Meta-Analysis (PRISMA) guidelines [25] (Table S1). The protocol was pre-registered at PROSPERO (CRD42022308388).

### Search strategy

An initial systematic literature search was performed in PubMed, Embase, and Web of Science on May 17, 2023. A subsequent updated search was conducted on April 11, 2025, to ensure identification of all relevant studies. The search combined controlled vocabulary terms and free-text keywords organized into three concept blocks: cancer, muscle mass or sarcopenia, and chemotherapy or immunotherapy. The complete search strategies for all databases are provided in Table S2.

### Eligibility criteria

Studies were eligible if they were published in peer-reviewed journals, included adults with cancer receiving chemotherapy or immunotherapy between baseline and follow-up assessments of SMM or prevalence of low SMM, and reported absolute values at both timepoints. To be included in the meta-analysis, studies also had to provide corresponding measures of variability (e.g. standard deviation [SD] or range). Combination regimens with targeted agents were accepted; studies in which ≥ 10% of patients received targeted therapy were classified as chemotherapy + targeted therapy or immunotherapy + targeted therapy. Eligible studies were required to quantify SMM using an objective method – computed tomography (CT), magnetic resonance imaging (MRI), dual-energy X-ray absorptiometry (DXA), or bioelectrical impedance analysis (BIA). Studies involving surgery or radiotherapy between SMM assessments were excluded to isolate the effects of systemic therapy. Studies not obtainable in English or a Scandinavian language, or those involving structured physical exercise or nutritional interventions, were excluded. Complete inclusion and exclusion criteria are provided in Table S3.

### Selection of studies

The selection of studies was performed using Covidence software. After duplicate removal, two independent reviewers (LS, SH, SJ, VS, GA, and BSR) screened titles and abstracts, followed by full-text assessment according to eligibility criteria. Disagreements were resolved through discussion or consultation with a third reviewer until consensus was reached. Interrater reliability between reviewers was assessed using Cohen’s kappa coefficient (κ), which ranges from 0 to 1, with values of 0.01–0.20 indicating slight, 0.21–0.40 fair, 0.41–0.60 moderate, 0.61–0.80 substantial, and 0.81–1.00 almost perfect agreement.

### Data extraction

Data were extracted using a standardized Microsoft© Excel spreadsheet by two independent reviewers (LS, SH, SJ, VS, GA, and BSR). Disagreements were resolved through discussion or consultation with a third reviewer. When essential data were missing, two contact attempts by email were made to obtain additional information. The following data were extracted: author, year, study design, country, patient setting, sample size, sex, age, cancer type and stage, treatment regimen and number of cycles, SMM assessment method and anatomical site (e.g. skeletal muscle index [SMI] and area [SMA], pectoralis muscle area [PEMA], psoas muscle index [PMI] and area [PMA], and lumbar muscle volume [LMV]), time between assessments, outcome type and cut-off values, baseline and follow-up SMM, correlation coefficient between baseline and follow-up, low SMM prevalence, corresponding p-values and confidence intervals, data source, and funding.

### Risk of bias

Risk of bias was assessed independently by at least two reviewers using the National Heart, Lung, and Blood Institute (NHLBI) Quality Assessment Tool for Observational Cohort and Cross-Sectional Studies [26]. Individual items were rated as ‘yes’, ‘no’, or ‘other’ (not reported, not applicable, or not determinable). An overall study rating was then assigned based on the proportion of criteria rated ‘yes’: ≥ 75% indicates good quality, 50–74% fair quality, and < 50% poor quality [27]. We tailored the risk-of-bias assessment to the specific objective of this review, which addressed within-patient changes in SMM rather than exposure–outcome associations. Therefore, items related to confounding (of the exposure-outcome association) were considered not applicable in terms of risk of bias relevant to the data we extracted for this review. Further, sample size justification was considered not applicable due to the retrospective population-based study designs, and loss to follow-up was not relevant due to availability of two measurements being an inclusion criterion for this review. However, we recognize that the study designs included in this review combined with inclusion criteria of two available SMM measurements carry considerable risk of bias, which we highlight in the discussion section.

### Statistics

Random-effects meta-analyses were conducted using the restricted maximum-likelihood (REML) estimator with Hartung–Knapp adjustment. A random-effects model was chosen because substantial clinical and methodological heterogeneity was expected across cancer types, treatment regimens, and body composition assessment methods. Continuous outcomes were synthesized as standardized mean changes (SMC) with 95% confidence intervals (CI), relative to the baseline SD. Effect sizes were interpreted according to Cohen’s thresholds (0.2 = small, 0.5 = moderate, 0.8 = large). Because the standard error (SE) depends on the correlation between baseline and follow-up values, we calculated the mean correlation coefficient from studies that reported it and imputed this value to studies without available data. To assess robustness, sensitivity analyses were performed using correlation coefficients of 0.1, 0.5, and 0.9. Studies reporting medians with interquartile ranges (IQR) or ranges, means, and SDs were estimated using the method described by Wan and colleagues [28]. Between-study variance (τ^2^) was estimated using REML. Statistical heterogeneity was further quantified using *I*^2^, with thresholds of 0–40% (might not be important), 30–60% (may represent moderate heterogeneity), 50–90% (may represent substantial heterogeneity), and 75–100% (considerable heterogeneity). Potential sources of heterogeneity were explored through predefined subgroup analyses (cancer type, treatment type, treatment setting, assessment tool, and sex) followed by sensitivity analyses (estimated means, non-small trials, imputed correlation coefficient, and study design). Prediction intervals were calculated to illustrate the expected range of true effects in future comparable studies. Publication bias was assessed by visual inspection of funnel plots and with Egger’s test. Percentage change in SMM was calculated from reported baseline and follow-up values when not explicitly provided in the original studies, using the formula: percentage change = ((follow-up − baseline)/baseline) × 100. All analyses were conducted in R (version 4.4.1; RStudio version 2025.09.0, Posit Software) using the meta package.

## Results

**Figure 1:**
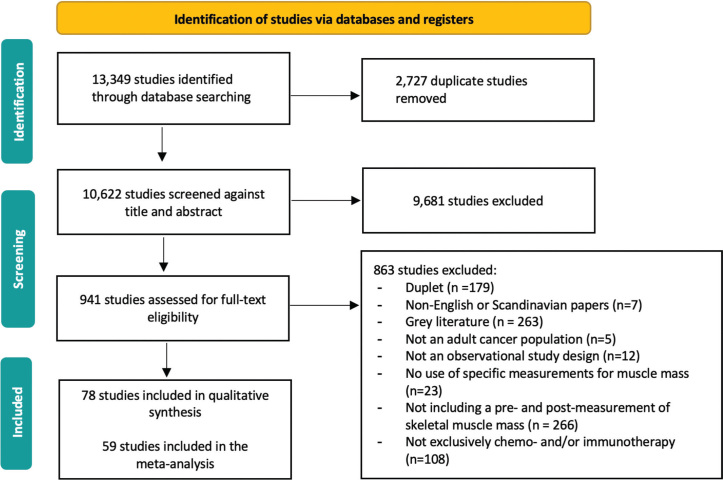
PRISMA flowchart.

The search yielded 13,349 records. After removal of duplicates and exclusion of ineligible studies, 78 studies were included (Figure 1). Study characteristics are summarized in Table 1 and Table S4. Seventy-eight studies (*n* = 10,502; 52% male; median age 64 years, IQR: 34–77) were included. Most studies (*n* = 65, 83%) were retrospective cohorts, and publications spanned from 2007 to 2025, with 58 (74%) published between 2019 and 2025. Interrater reliability was moderate for title and abstract screening (Cohen’s κ = 0.43) and substantial for full-text screening (Cohen’s κ = 0.79). Most studies were of good methodological quality (median rating 91%) (Table S5).

**Table 1:** Study characteristics.

Author (year)/country	Study design	*N* (male/ female)	Age (± SD) or min-max^a^	Cancer type	Cancer stage (TNM unless otherwise specified)	Setting	Planned treatment regimen	Cycle(s)
**Pancreatic cancer**								
Chemotherapy								
Shimura (2023) [51]/Japan	Retro	75 (40/35)	67 (± 8)	Pancreatic cancer	UICC 8th stage: I: 17, II: 34, III: 7, IV: 16	Neo	Gemcitabine + S-1	1
Jin (2021) [52]/ China	Retro	119 (59/60)	60 (± 8)	Pancreatic cancer	NR	Neo	Nab-paclitaxel + gemcitabine; gemcitabine-based; FOLFIRINOX (multi-agent)	NR
Griffin (2019) [53]/ Ireland	Retro	78 (37/41)	64 (± 8)	Pancreatic cancer	Tumor stage: I: 6, II:2, III:17, IV: 0	Neo	FOLFIRINOX; nab-paclitaxel + gemcitabine; gemcitabine monotherapy; gemcitabine + platinum	NR
Lee (2024) [40]/ South Korea	Retro	456 (272/184)	61 (± 10)	Pancreatic cancer	Metastatic	Palliative	FOLFIRINOX or gemcitabine + nab-paclitaxel (multi-agent)	NR
Rollins (2016) [54]/ UK	Retro	98 (55/44)	65 (± 9)	Pancreatic cancer	Locally advanced: 60, Metastatic: 38	Palliative	Gemcitabine-based	NR
Aberle (2025) [55]/Netherlands	Retro	52 (31/21)	64 (± 13)	Pancreatic cancer	Locally advanced: 27, Metastatic: 25	NR	FOLFIRINOX (multi-agent)	4
Davis (2025) [56]/ USA	Retro	103 (58/45)	68 (± 11)	Pancreatic cancer	I:5, II:35, III:15, IV:43	NR	Single-agent irinotecan/gemcitabine/oxaliplatin/paclitaxel; combinations incl. gemcitabine + paclitaxel ± irinotecan/oxaliplatin	NR
Uemura (2020) [23]/Japan	Retro	69 (38/31)	63 (38–74)	Pancreatic cancer	IV	NR	FOLFIRINOX (multi-agent)	Every 2 weeks
Lee (2019) [41]/ South Korea	Retro	57 (32/25)	61 (38–78)	Pancreatic cancer	(first line) II:12, III:11, IV:34	NR	FOLFIRINOX (multi-agent)	4 (3–6)
**Urological cancer**								
Chemotherapy								
Miyake (2018) [57]/ Japan	Retro	14 (12/2)	73 (64–77)	Advanced urothelial cancer	Clinical T II:8, III:3, IV:3	Neo	Gemcitabine + cisplatin/carboplatin (GC/GCa, platinum-based)	3
MacDonald (2024)/Canada [58]	Retro	70 (59/11)	65 (± 8)	Muscle-invasive bladder cancer	Clinical T cT1:9, cT2:50, cT3:6, cT4:5	Neo	Gemcitabine + cisplatin (97%) or gemcitabine + carboplatin (3%) (platinum-based)	2
Rimar (2018) [59]/ USA	Retro	26 (19/7)	67 (40–82)	Muscle-invasive bladder cancer	Clinical T T2:18, T3:8, N1:8	Neo	MVAC; gemcitabine + cisplatin; gemcitabine + carboplatin (platinum-based)	3–5
Lyon (2019) [60]/ USA	Retro	183 (155/28)	65 (57–72)	Muscle-invasive bladder cancer	Clinical T II:98, III:54, IV:18	Neo	Gemcitabine + cisplatin (majority); MVAC or related regimens (platinum-based)	4 (1–4)
Takai (2021) [38]/ Japan	Retro	44 (44/0)	37 (19–80)	Testicular cancer	II:10, III:9, IV:18	Adjuvant	BEP/EP; VIP/TIP/VeIP; GEMOX; other cisplatin-based regimens	4 (1–14)
Mitsui (2019) [61]/ Japan	Retro	50 (50/0)	34 (16–67)	Testicular cancer	Clinical S 0:1, I: 17, II :17, III: 8	NR	NR	2–4
Semerad (2022) [62]/ Czhechia	Pro	30 (30/0)	37 (22–60)	Testicular cancer	I: 7, II: 9, III: 7	NR	BEP (bleomycin, etoposide, cisplatin) (platinum-based)	3 (2–4)
Buxton (2024) [63]/ USA	Retro	182 (182/0)	31 (26–39)	Testicular cancer	Stage 1–IS:33; II–IIC:74; III–IIIC:71; Unknown:4	NR	BEP, EP, VIP, or related cisplatin-based regimens	3
**Lung cancer and pleural mesothelioma**								
Chemotherapy								
Goncalves (2018) [37]/USA	Retro	88 (42/46)	65 (55–71)	Non-Small Cell Lung cancer	I:12, II:15, III:58, IV:3	Neo	Taxane- or gemcitabine-based; *N* = 7, 8% received bevacizumab	2–6
Stene (2015) [64]/Norway	Pro	35 (18/17)	67 (± 7)	Non-Small Cell Lung cancer	IIIB: 6 IV: 29	Palliative	Carboplatin; vinorelbine; gemcitabine (platinum-based)	1–3
Kazemi-Bajestani (2019) [65]/Canada	Pro	50 (24/26)	65 (± 8)	Non-Small Cell Lung cancer	IV	Palliative	Carboplatin doublets: with vinorelbine, gemcitabine, paclitaxel, or pemetrexed (platinum-based)	1–4
Nattenmüller (2017) [66]/Germany	Retro	200 (130/70)	62 (± 10)	Non-Small Cell Lung cancer	UICC I:3, II:10, III: 43, IV: 144	NR	Carboplatin/cisplatin with gemcitabine, vinorelbine, etoposide, pemetrexed, or others (platinum-based)	1–8
Kidd (2024) [67]/UK	Retro	111 (91/20)	69 (63–72)	Pleural mesothelioma	I:45, II:22, III:12, IV:19	NR	Cisplatin or carboplatin with pemetrexed (platinum-based)	NR
Kakinuma (2018) [35]/Japan	Retro	44 (31/13)	67 (± 8)	Non-Small Cell Lung cancer	IV	NR	(Only chemotherapy cohort) Carboplatin or cisplatin with pemetrexed, gemcitabine, paclitaxel/nab-paclitaxel	NR
Immunotherapy								
Khan (2023) [68]/Australia	Retro	97 (55/42)	68 (± 10)	Non-Small Cell Lung cancer	III:15 IV:81	Palliative	Immune checkpoint inhibitors	NR
Chemotherapy + immunotherapy								
Chaunzwa (2024) [20]/USA	Retro	1,791 (913/878)	65 (26–84)	Non-Small Cell Lung cancer	Advanced or metastatic	Palliative	Chemotherapy (SOC); chemo-immunotherapy; or immunotherapy monotherapy	NR
Chemotherapy + targeted therapy								
Cortellini (2018) [69]/Italy	Retro	81 (53/28)	68 (39–90)	Non-Small Cell Lung cancer	IV	NR	Platinum doublets (pemetrexed, gemcitabine, paclitaxel + bevacizumab); or single agents (carboplatin, docetaxel, vinorelbine) (platinum-based)	NR
**Gastric, esophagogastric and esophageal cancers**								
Chemotherapy								
Sato (2024) [70]/Japan	Pro	50 (39/11)	64 (38–75)	Locally advanced gastric cancer	cStage (14^th^) II:1, III:27, IV:22	Neo	Docetaxel + cisplatin + S-1 (DCS, platinum-based)	2
Juez (2024) [71]/Spain	Retro	61 (34/27)	68 (± 9)	Locally advanced gastric cancer	AJCC I:10, II:30, III:21	Neo	FLOT (docetaxel, oxaliplatin, leucovorin, 5-FU)	4
Li (2024) [17]/China	Retro	345 (109/236)	61 (± 12)	Advanced gastric cancer	II:189, III:156	Neo	SOX (S-1 + oxaliplatin), XELOX (capecitabine + oxaliplatin), or FOLFOX (5-FU + leucovorin + oxaliplatin)	4–6
Mirkin (2017) [72]/USA	Retro	36 (13/23)	65 (NR)	Advanced gastric cancer	NR	Neo	Epirubicin + cisplatin + 5-FU (epirubicin-based)	NR
Matsuura (2020) [73]/Japan	Retro	41 (28/13)	72 (48–82)	Advanced gastric cancer	II:9, III:25, IV:7	Neo	S-1 + cisplatin; S-1 + docetaxel + cisplatin; or S-1 + oxaliplatin (platinum-based)	2 (1–4)
Horii (2022) [74]/Japan	Retro	38 (28/10)	64 (44–78)	Advanced gastric cancer	Clinical stage (stage J) II: 18, III: 13: IV:7	Neo	DCS (S-1 + cisplatin + docetaxel), DS (S-1 + docetaxel), XP (capecitabine + cisplatin), SP (S-1 + cisplatin, platinum-based)	2
Sugiyama (2018) [16]/Japan	Retro	118 (69/48)	64 (27–84)	Advanced gastric cancer	Metastatic	Palliative	Fluoropyrimidine + cisplatin; oxaliplatin (platinum-based)	NR
Park (2020) [39]/Korea	Retro	111 (80/31)	65 (31–87)	Advanced gastric cancer	IV	Palliative	S-1 + cisplatin; XP; FOLFOX/XELOX; S-1 or capecitabine alone; *N* = 11, 9.9% received trastuzumab + XP	NR
Palmela (2017) [75]/Portugal	Retro	47 (32/15)	68 (± 10)	Esophagogastric cancer	III:5, IV:42	Neo	ECF, EOF, EOX, ECX, XELOX, FOLFOX, capecitabine, or DCF	2
den Boer (2020) [76]/ NR	Retro	199 (158/41)	66 (28–80)	Esophagogastric cancer	Clinical T T1:1, T2:62, T3:107, T4: 28	Neo	ECX, CX, or related platinum–fluoropyrimidine regimens	1–4
Rinninella (2021) [77]/Italy	Retro	26 (18/8)	63 (± 11)	Esophagogastric cancer	Pathologic stage: 0: 0, 1: 4, 2: 5, 3: 6, 4: 9, missing: 2	Neo	FLOT (perioperative regimen)	4
Fujihata (2021) [78]/Japan	Retro	99 (89/10)	68 (61–72)	Esophagogastric cancer	Pathologic stage 0:3, I:12, II:41, III:43	Neo	5-FU + cisplatin (FP) or docetaxel + cisplatin + 5-FU (DCF, platinum-based)	1–2
Dijksterhuis (2019) [79]/Netherlands	Retro	88 (66/22)	63 (56–69)	Esophagogastric cancer	Metastatic	Palliative	Capecitabine + oxaliplatin (CAPOX, platinum-based)	1–6
Hacker (2022) [80]/Germany	Pro	509 (387/122)	< 65: 375 ≥ 65: 134	Esophagogastric cancer	Metastatic	Palliative	Platinum–fluoropyrimidine chemotherapy (platinum-based)	NR
Awad (2012) [81]/ UK	Retro	47 (34/17)	63 (± 12)	Esophagogastric cancer	T3 N0/1: 23, T2 N0/1: 10, T3 N2/3: 5, T1 N0: 5, T4 N1: 1, Tis N0: 1, No residual tumor: 2	Neo	Epirubicin + cisplatin + 5-FU; cisplatin + 5-FU; capecitabine + cisplatin; epirubicin + oxaliplatin	1–4
Onishi (2024) [82]/ Japan	Retro	215 (178/37)	67 (40–81)	Esophageal cancer	DCF: II:11, III:58; CF: II:80, III:66	Neo	Docetaxel + cisplatin + 5-FU (DCF) or cisplatin + 5-FU (CF, platinum-based)	2–3
Harada (2025) [83]/Japan	Retro	69 (53/16)	73 (4)	Esophageal cancer	Clinical stage IB, II, III, or IV without distant organ metastasis	Neo	Cisplatin + 5-FU (FP), FOLFOX, or DCF (platinum-based)	2–4
Yip (2014) [84]/ UK	Retro	35 (30/5)	63 (34–78)	Esophageal cancer	II:10, III:23, IV:2.	Neo	ECF/ECX (epirubicin, cisplatin, 5-FU/capecitabine) (platinum-based)	3 (1–6)
Miyata (2017) [85]/ Japan	Retro	94 (76/18)	64 (± 9)	Esophageal cancer	I:5, II:24, III:54, IV:11	Neo	Adriamycin + cisplatin + 5-FU (ACF) or docetaxel + cisplatin + 5-FU (DCF, platinum-based)	2 (1–3)
Ishida (2019) [86]/Japan	Retro	165 (144/21)	65 (NR)	Esophageal cancer	Clinical T I+II:14; III+IV:29	Neo	DCF vs. ACF (platinum-based)	2
Chemotherapy + immunotherapy								
Zhao (2024) [87]/China	Retro	85 (69/16)	67 (59–71)	Esophageal cancer	II:37, III:40, IV:8	Neo	PD-1 inhibitor (camrelizumab) + platinum + paclitaxel (platinum-based)	2–4
Ying (2025) [88]/China	Retro	83 (81/2)	68 (49–87)	Esophageal cancer	Lymph node metastasis: 98% Distant metastasis: 37%	NR	PD-1 inhibitor + chemotherapy	NR
**Ovarian cancer**								
Chemotherapy								
Wood (2023) [89]/USA	Retro	174 (0/174)	64 (± 10)	Ovarian cancer	II:1, III:108, IV:65	Neo	NR	NR
Ubachs (2020) [90]/Netherlands	Retro	212 (0/212)	61 (± 8)	Ovarian cancer	FIGO III:212	Neo	Carboplatin + paclitaxel.	2
Yoshino (2020) [91]/Japan	Retro	60 (0/60)	64 (43–81)	Ovarian cancer	FIGO III:36, IV:24	Neo	Carboplatin + paclitaxel/docetaxel/irinotecan (platinum-based)	4 (2–6)
Del Grande (2021) [92]/Switzerland	Retro	25 (0/25)	65 (± 11)	Ovarian cancer	FIGO I: 1, II:3, III:45, IV:20.	Neo	Platinum-based	NR
Van der Zanden (2021) [93]/Netherlands	Retro	111 (0/111)	77 (74–79)	Ovarian cancer	FIGO III:73, IV:38	Neo	Platinum-based	2
**Studies including multiple cohorts with multiple cancers**								
Chemotherapy								
Toama (2022) [94]/ USA	Retro	474 (161/313)	61 (53–68)	Breast cancer (*n* = 192) Lymphoma (*n* = 184) Sarcoma (*n* = 98)	I:49, II:73, III:84, IV:234, Missing: 34	NR	Anthracycline-based chemotherapy	NR
Immunotherapy								
Loosen (2021) [19]/Germany	Pro	88 (48/42)	67 (34–87)	Lung: 39.8% Malignant melanoma: 15.9% Urothelial cancer: 13.6% GI cancer: 13.6% Head and neck cancer: 8.0% Others: 9.1%	UICC III: 7%, UICC IV: 93%	NR	Nivolumab, pembrolizumab, nivolumab + ipilimumab, or others	NR
Chemotherapy + immunotherapy								
Roeland (2021) [95]/USA	Pro	38 (20/18)	62 (± 2)	Gastrointestinal: 71% Lung: 13% Gynecologic: 8% Head and neck: 8% Other: 5%	Metastatic	NR	NR	NR
Chemotherapy + targeted therapy								
Oflazoglu (2020) [96]/Turkey	Pro	276 (122/154)	57 (± 11)	Breast: 33.7% Colorectal: 26.8% Pancreaticobiliary: 10.9% Urological: 8.7% Gastroesophageal: 7.6% Lung: 3.6% Head and neck: 2.5% Others: 6.2%	Metastasis status: No: 229 Yes: 47	NR	Site-specific regimens: breast (AC + paclitaxel ± trastuzumab), colorectal (XELOX, FOLFOX ± bevacizumab/panitumumab, capecitabine), pancreaticobiliary (cisplatin-based, gemcitabine, FOLFIRINOX), urological (platinum-based), gastroesophageal (cisplatin-based, XELOX), lung (cisplatin-based, carboplatin + paclitaxel), head & neck (cisplatin-based), others	NR
**Melanoma**								
Immunotherapy								
Daly (2017) [97]/Ireland	Retro	84 (52/32)	54 (43–66)	Melanoma	M1a: 9, M1b: 9 , M1c: 66	NR	Ipilimumab (CTLA-4 inhibitor)	4
**Liver cancer**								
Immunotherapy								
Chen (2025) [98]/China	Retro	85 (68/17)	62 (± 12)	Intermediate and advanced liver cancer	Child-Pugh score: A: 48, B: 31, C: 6	NR	PD-1 inhibitors (sintilimab, tislelizumab, camrelizumab, pembrolizumab) or PD-L1 inhibitors (atezolizumab, durvalumab)	NR
Immunotherapy + targeted therapy								
Shigefuku (2024) [99]/Japan (ATZ-BEV)	Retro	56 (45/11)	74 (69–80)	Advanced liver cancer	Child–Pugh score: 5: 52, 6: 37, 7: 7, 8: 1	NR	Atezolizumab + bevacizumab	NR
**Colorectal cancer**								
Chemotherapy								
Okuno (2019) [100]/USA	Retro	169 (97/72)	56 (± 12)	Colorectal cancer	NR	Neo	Oxaliplatin-based; irinotecan-based; multiple regimens (platinum-based)	6 (2–24)
Chemotherapy + targeted therapy								
Nozawa (2021) [101]/Japan	Retro	98 (58/40)	65 (28–88)	Colorectal cancer	IV	Neo Conversion Palliative	FOLFOX; CAPOX; SOX; FOLFIRI; FOLFOXIRI; IRIS (platinum- or irinotecan-based ± targeted)	NR
Palle (2016) [102]/Denmark	Pro	18 (10/8)	67 (± 6)	Colorectal cancer	Patients with tumor stage T3–4, N0–N1 and/or V0–V1.	Adjuvant	Capecitabine; capecitabine + oxaliplatin; capecitabine + oxaliplatin + bevacizumab (platinum-based)	1–8
Huemer (2019) [103]/Austria	Retro	10 (6/4)	65 (42–81)	Colorectal cancer	Metastatic	NR	TAS-102 (trifluridine/tipiracil); regorafenib	NR
Blauwhoff-Buskermolen (2016) [21]/Netherlands	Pro	67 (42/25)	66 (± 11)	Colorectal cancer	Metastatic	Palliative	CAPOX ± bevacizumab; FU + oxaliplatin ± bevacizumab; capecitabine + irinotecan; irinotecan monotherapy; capecitabine ± bevacizumab (platinum-based)	NR
Gallois 2021 [104]/France	Pro	149 (82/67)	70 (NR)	Colorectal cancer	Metastatic	NR	5-FU-based regimens: oxaliplatin-based; irinotecan-based; single agent; doublet; triplet; ± bevacizumab; ± cetuximab/panitumumab (platinum/irinotecan-based)	NR
**Breast cancer**								
Chemotherapy								
Jang (2022) [105] (AC-T)/South Korea	Retro	214 (0/214)	53 (± 11)	Breast cancer	I:3, II:153, III:51	Neo	Anthracycline–cyclophosphamide → taxane (AC-T)	6
Campbell (2007) [106]/Canada	Pro	10 (0/10)	47 (± 6)	Breast cancer	I: 2, II-IIIA: 8	Adjuvant	CEF or AC (anthracycline-based)	5
Jung (2020) [107]/South Korea	Pro	37 (0/37)	51 (± 9)	Breast cancer	I:13, II:22, III:2	Adjuvant	AC or TC (docetaxel + cyclophosphamide)	NR
Chemotherapy + immunotherapy (+ targeted therapy)								
Camilleri (2024) [108]/France	Retro	111 (0/111)	60 (12)	Breast Cancer	Metastatic	Palliative	Not otherwise specified	NR
Chemotherapy + targeted therapy								
Zhang (2024) [109]/China	Retro	43 (0/43)	51 (± 10)	Breast cancer	II-III	Neo	Taxanes and anthracyclines. HER2-positive received targeted therapy with trastuzumab and pertuzumab.	6–8
Rossi (2023) [110]/Italy	Retro	52 (0/52)	37 (± 5)	Breast cancer	NR	Neo	Epirubicin + cyclophosphamide (EC) → paclitaxel; some with carboplatin + paclitaxel; HER2+ with trastuzumab	4
Karaca (2024) [111]/Turkey	Retro	226 (0/226)	50 (± 12)	Breast cancer	II–III	Neo	Anthracycline–cyclophosphamide → paclitaxel/docetaxel; HER2+ with trastuzumab/pertuzumab	NR
Amitani (2022) [18]/Japan	Retro	141 (0/141)	53 (± 10)	Breast cancer	II:89, III:52	Neo	FEC/EC → taxane (docetaxel or paclitaxel); HER2+ with trastuzumab	4
Lee (2021) [112]/ South Korea	Retro	246 (0/246)	48 (42–54)	Breast cancer	I:9, II:123, III:114	Neo	AC ± paclitaxel; subset with trastuzumab	NR
Rossi (2020) [113]/ Italy	Retro	101 (0/21)	56 (± 11)	Breast cancer	NR	Neo	EC → paclitaxel; subset with pertuzumab + trastuzumab	4–6
Mazzuca (2018) [114]/ Italy	Retro	21 (0/21)	54 (39–72)	Breast cancer	I:7, II:11, III:3	Adjuvant	FEC/EC; EC + taxane; ~43% with trastuzumab	4
**Lymphoma**								
Chemotherapy + targeted therapy								
Xiao (2016) [115]/ USA	Retro	342 (331/11)	63 (± 11)	Diffuse large B-cell lymphoma	Clinical stage I+II:145; III+IV:195, 2: missing	NR	CHOP (cyclophosphamide, doxorubicin, vincristine, prednisone) ± rituximab (R-CHOP)	NR

TNM: Tumor, Node, and Metastasis; N: number of participants at baseline; SD: standard deviation; NR: not reported; Neo: neoadjuvant treatment; Adjuvant: adjuvant chemotherapy; Retro: Retrospective cohort; Pro: prospective cohort.

a rounded to the nearest whole number.

**Figure 2:**
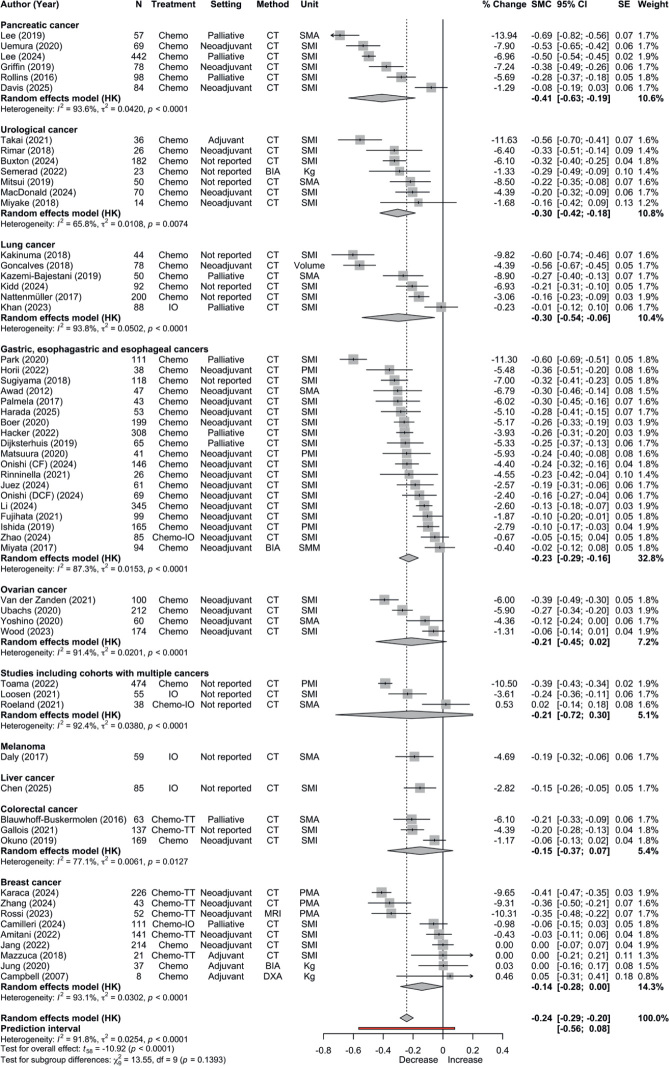
Meta-analyses of the change in skeletal muscle mass during systemic cancer treatment.

Across cancer types, systemic treatment with chemotherapy and/or immunotherapy (± targeted therapy) was associated with a decline in SMM (59 studies; *n* = 6,373; SMC = –0.24, 95% CI: –0.29 to –0.20; *I*^2^ = 92%), corresponding to an unweighted median of −5% (IQR: −7 to −2) over a median interval of 90 (IQR: 71–129) days. A total of 30 (38%) studies did not report the interval between SMM assessments**.** The 95% prediction interval (–0.56 to 0.08) indicated between-study variability in the magnitude and direction of effects (Figure 2 and Table 2). Most studies (*n* = 70, 89%) assessed SMM using CT imaging, predominantly at the third lumbar vertebra (L3) (*n* = 63, 73%). The SMI was the most common outcome measure, reported in 51 (65%) studies. Assessment methods and outcomes are presented in Table 3. No evidence of publication bias was identified by Egger’s test (*p* = 0.229) or visual inspection of the funnel plot (Figure S1). Among the five studies [28–32] reporting the correlation coefficient between repeated measurements, the mean correlation was 0.88. Sensitivity analyses using assumed correlations of 0.1, 0.5, and 0.9 yielded nearly identical pooled estimates, with only slight increases in SE.

**Table 2:** Meta-analyses of the change in skeletal muscle mass during systemic cancer treatment.

Meta-analyses	*N*	SMC (95% CI)	Comparisons	*I* ^2^	*P*
Primary analysis	6,373	−0.24 (−0.29 to −0.20)	59 [16–19, 21, 23, 35, 37–41, 53, 54, 56–59, 61–63, 65–68, 71,, 73–83, 85–87, 89–91, 93–95, 97, 98, 100, 104–111, 114]	92%	-
**Subgroup analyses^a^**					
Treatment type					
Chemotherapy	5,169	−0.27 (−0.32 to −0.22)	45 [16, 17, 23, 35, 37–41, 53, 54, 56–59, 61–63, 65–67, 71, 73–83, 85, 86, 89–91, 93, 94, 100, 105–107]	92%	-
IO	287	−0.15 (−0.30 to 0.01)	4 [19, 68, 97, 98]	63%	-
Chemo-IO	234	−0.05 (−0.13 to 0.04)	3 [87, 95, 108]	0%	-
Chemo-TT	683	−0.23 (−0.38 to −0.08)	7 [18, 21, 104, 109–111, 114]	91%	-
Treatment setting					
Neoadjuvant	3,321	−0.22 (−0.28 to −0.17)	32 [17, 18, 23, 37, 53, 56–59, 71, 73–78, 81–83, 85–87, 89–91, 93, 100, 105, 109–111]	90%	-
Adjuvant	102	−0.14 (−0.61 to 0.33)	4 [38, 106, 107, 114]	91%	-
Palliative	1,393	−0.31 (−0.47 to −0.16)	10 (21, 39–41, 54, 65, 68, 79, 80, 108)	95%	-
Not reported	1,557	−0.25 (−0.34 to −0.17)	13 [16, 19, 35, 61–63, 66, 67, 94, 95, 97, 98, 104]	86%	-
Assessment tool					
CT SMI (cm/m^2^)	4,670	−0.23 (−0.29 to −0.18)	39 [16–19, 23, 35, 38–40, 53, 54, 56–59, 63, 66–68, 71, 75–80, 82, 83, 87, 89, 90, 93, 98, 100, 104, 105, 108, 114]	92%	-
CT SMA (cm^2^)	424	−0.25 (−0.42 to −0.08)	8 [21, 41, 61, 65, 81, 91, 95, 97]	89%	-
CT PMI (cm/m^2^)	718	−0.27 (−0.49 to −0.05)	4 [73, 74, 86, 94]	93%	-
CT PMA (cm^2^)	269	−0.40 (−0.66 to −0.15)	2 [109, 111]	0%	
BIA (kg)	154	−0.09 (−0.49 to 0.29)	3 [62, 85, 107]	67%	-
**Sensitivity analyses**					
Estimated means^b^	3,968	−0.22 (−0.27 to −0.17)	40 [16, 18, 21, 23, 35, 38, 53, 54, 56, 58, 59, 62, 65, 66, 68, 75–77, 79–83, 85, 86, 89, 90, 95, 97, 98, 100, 104–111]	88%	-
Non-small trials^c^	2,286	−0.24 (−0.32 to −0.16)	20 [16–18, 39, 40, 63, 66, 76, 80, 82, 86, 89, 90, 93, 94, 100, 104, 105, 108, 111)	96%	-
Correlation coefficient estimation^d^	6,373	−0.25 (−0.29 to −0.20)	59 [16–19, 21, 23, 35, 37–41, 53, 54, 56–59, 61–63, 65–68, 71, 73–83, 85–87, 89–91, 93–95, 97, 98, 100, 104–111, 114]	72%	-
Prospective study design	917	−0.18 (−0.27 to −0.09)	9 [19, 21, 62, 65, 80, 95, 104, 106, 107]	61%	-
By sex (male)	438	−0.21 (−0.42 to −0.01)	8 [16, 21, 39, 55, 79, 101, 102, 104]	92%	
By sex (female)	263	−0.40 (−0.69 to −0.12)	8 [16, 21, 39, 55, 79, 101, 102, 104]	93%	
**Funnel plot asymmetry**					0.229

N: number with complete data; SMC: standardized mean change; CI: confidence interval; *I*^2^, heterogeneity; Chemo: chemotherapy; TT: targeted therapy; IO: immunotherapy; Multiple: studies combining multiple diagnoses; CT: computed tomography; CM: centimeter; SMI: Skeletal Muscle Index; SMA: Skeletal Muscle Area; PMI: Psoas (or Pectoralis) Muscle Index; PMA: Psoas (or Pectoralis) Muscle Area; BIA: bioimpedance analysis; Kg.: kilograms.

a Only subgroups with ≥ 2 studies are presented.

b Estimated means: Sensitivity analysis excluding studies where means and SDs were derived from medians/IQRs using Wan et al.’s method.

c Non-small studies: Sensitivity analysis excluding studies with *n* ≤ 100.

d Correlation coefficient estimation: Sensitivity analysis with imputed correlation coefficient (*r* = 0.5).

**Table 3:** Changes in skeletal muscle mass during systemic cancer treatment for studies reporting continuous pre- and post-treatment skeletal muscle mass.

Author (year)*	Assessment method	Outcome measure	Body segment	Days between measurements	Pre-treatment muscle mass		Post-treatment muscle mass		Change mean or median	Change %	*P*
					Mean or Median	SD or min–max	Mean or median	SD or min–max			
**Pancreatic cancer**											
Chemotherapy											
Shimura (2023) [51]	CT	SMI	L3	NR	M: 45.70 F: 35.70	M: 9.70 F: 5.70	M: 41.10 F: 34.90	M: 8.80 F: 6.10	NR	−10.07%** −2.24%**	M: < 0.01 F: 0.153
Griffin (2019) [53]	CT	SMI	L3	128	45.60	8.70	42.30	9.30	NR	−7.24%**	< 0.01
Lee (2024) [40]	CT	SMI	L3	60	43.10	39.10–49.90	40.10	35.90–45.00	NR	−6.96%**	< 0.01
Rollins (2016) [54]	CT	SMI	L3	60	42.20	8.60	39.80	8.00	NR	−5.69%**	0.060
Aberle (2025) [55]	CT	SMI	L3	90	M: 49.10 F: 39.20	M: 8.80 F: 4.70	M: 45.90 F: 35.50	M: 8.70 F: 4.20	M: −2.20 F: −3.50	−6.52%** −9.44%**	M: 0.002 F: 0.001
Davis (2025) [56]	CT	SMI	L3	71.50	46.60	7.65	46.00	7.94	−0.90	−1.29%**	NR
Uemura (2020) [23]	CT	SMI	L3	71	40.20	7.30	36.30	6.30	NR	−7.90%	NR
Lee (2019) [41]	CT	SMM	L3	60	100.40	20.30	86.40	20.20	NR	−13.94%**	< 0.001
**Urological cancer**											
Chemotherapy											
Miyake (2018) [57]	CT	SMI	L3	NR	53.70	48.80–58.00	52.80	48.80–55.20	NR	−1.68%**	0.016
MacDonald (2024) [58]	CT	SMI	L3	69	52.40	10.80	50.10	10.10	−2.2 (± 3.2)	−4.39%**	< 0.001
Rimar (2018) [59]	CT	SMI	L3	110	49.10	NR	44.50	NR	NR	−6.40%	< 0.01
Lyon (2019) [60]	CT	SMI	L3	139	50.70	NR	48.60	NR	NR	−4.14%**	NR
Takai (2021) [38]	CT	SMI	L3	182	51.60	28.60–70.60	45.60	32.40–60.60	NR	−11.63%**	NR
Mitsui (2018) [61]	CT	SMA	L3	21	150.20	76.30–206.90	140.50	73.40–200.70	NR	−8.50%	NR
Semerad (2022) [62]	BIA	SMM kg	Whole body	NR	32.67	4.59	31.34	4.65	NR	−1.33%**	0.005
Buxton (2024) [63]	CT	SMI	L3	114	57.50	52.50–62.90	55.00	49.30–60.70	−3.60 (−6.50 to 0.40)	−6.10%	< 0.001
**Lung cancer and pleural mesothelioma**											
Chemotherapy											
Goncalves (2018) [37]	CT	LMV	T10–L1	NR	296.50	249.00–369.00	283.00	241.00–337.00	NR	−4.39%	NR
Stene (2015) [64]	CT	SMA	L3	88	121.90	30.80	117.40	NR	NR	−3.69%**	< 0.01
Kazemi−Bajestani (2019) [65]	CT	SMA	L3	112	130.50	36.00	120.90	29.70	NR	−8.90%	< 0.01
Nattenmüller (2017) [66]	CT	SMI	L2–L3	129	45.70	8.70	44.30	8.60	NR	−3.06%**	< 0.01
Kidd (2024) [67]	CT	SMI	L3	NR	50.50	44.00–57.80	47.00	42.00–57.00	NR	−6.93%**	< 0.01
Kakinuma (2018) [35]	CT	SMI	L3	132	44.80	7.30	40.40	6.60	NR	−9.82%**	NR
Immunotherapy											
Khan (2023) [68]	CT	SMI	L3	NR	43.80	8.50	43.70	9.40	−0.10	−0.23%**	NR
**Gastric, esophagogastric and esophageal cancers**											
Chemotherapy											
Sato (2024) [70]	CT	SMI	L3	NR	47.90	NR	44.10	NR	NR	−3.40%	NR
Juez (2024) [71]	CT	SMI	L3	NR	46.69	40.10–55.20	45.52	39.00–51.00	NR	−2.57%	< 0.01
Li (2024) [17]	CT	SMI	L3	NR	40.40	32.10–44.20	39.60	30.50–43.10	−1,3	−2.60%	< 0.01
Matsuura (2020) [73]	CT	PMI	L3	NR	4.77	1.11	4.50	1.20	NR	−5.93%	< 0.01
Horii (2022) [74]	CT	PMI	Umbilicus level	60	6.57*	3.84–9.74	6.21*	3.30–8.89	NR	−5.48%**	< 0.01
Sugiyama (2018) [16]	CT	SMI	L3	429	39.00	8.02	36.40	8.05	NR	−7.00%	< 0.01
Park (2020) [39]	CT	SMI	L3	NR	40.70	9.00	35.30	8.30	NR	−11.30%	< 0.01
Palmela (2017) [75]	CT	SMI	L3	86.40	48.20	9.60	45.30	9.50	NR	−6.02%**	< 0.01
Boer (2020) [76]	CT	SMI	L3	105	51.87	10.31	49.19	9.71	NR	−5.17%**	< 0.01
Rinninella (2021) [77]	CT	SMI	L3	95.50	48.74	9.76	46.52	9.80	NR	−4.55%**	< 0.01
Fujihata (2021) [78]	CT	SMI	L3	NR	40.66	36.3–46.61	39.33	35.70–46.13	NR	−1.87%	NR
Dijksterhuis (2019) [79]	CT	SMI	L3	79.00	46.90	9.90	44.40	10.00	NR	−5.33%**	< 0.01
Hacker (2022) [80]	CT	SMI	L3	84	61.62	9.44	59.2	NR	NR	−3.93%**	NR
Awad (2012) [81]	CT	SMA	L3	107	140.00	31.70	130.50	28.00	NR	−6.79%**	< 0.01
Onishi (2024) [82] (DCF treatment)	CT	SMI	L3	NR	41.50	7.60	40.30	7.20	NR	−2.40%	NR
Onishi (2024) [82] (CF treatment)	CT	SMI	L3	NR	40.40	8.30	38.40	7.50	NR	−4.40%	NR
Harada (2025) [83]	CT	SMI	L3	NR	43.10	7.80	40.90	7.60	NR	−5.10%**	< 0.01
Miyata (2017) [85]	BIA	SMM	Whole body	77	25.00	4.80	24.90	4.80	NR	−0.40%**	NR
Ishida (2019) [86]	CT	PMI	L3	NR	7.17	2.01	6.97	1.86	NR	−2.79%**	< 0.01
Chemotherapy + immunotherapy											
Zhao (2024) [87]	CT	SMI	L3	NR	45.10	42.25–49.70	44.80	42.17–48.83	−0.12	−0.67%**	0.146
Ying (2025) [88]	CT	SMI	L3	NR	69.20	NR	65.40	NR	NR	−5.50%**	NR
**Ovarian cancer**											
Chemotherapy											
Wood (2023) [89]	CT	SMI	L4	93	38.30	7.90	37.80	7.90	NR	−1.31%**	NR
Ubachs (2020) [90]	CT	SMI	L3	60	39.60	5.40	38.10	5.00	NR	−5.90%	NR
Yoshino (2020) [91]	CT	SMA	L3	NR	87.20	52.50–129.00	83.40	59.20–122.00	NR	−4.36%**	0.019
Del Grande (2021) [92]	CT	SMI	L3	NR	48.00	8.90	45.00	68.10	NR	−6.25%**	0.052
Van der Zanden (2021) [93]	CT	SMI	L3	66	39.10	36.30–43.10	37.20	34.70–40.50	NR	−6.00%	0.001
**Studies including multiple diagnoses**											
Chemotherapy											
Toama (2022) [94]	CT	PMI	T2–T3	NR	5.80	4.90–7.70	5.20	4.40–6.40	NR	−10.50%	NR
Immunotherapy											
Loosen (2021) [19]	CT	SMI	L3	84	76.79	46.00–124.90	74.02	44.20–113.50	NR	−3.61%**	NR
Chemotherapy + immunotherapy											
Roeland (2021) [95]	CT	SMA	NR	90	132.10	35.90	132.80	36.30	NR	+0.53%**	0.648
**Melanoma**											
Immunotherapy											
Daly (2017) [97]	CT	SMA	L3	146	151.30	37.20	144.20	37.30	NR	−4.69%**	NR
**Liver cancer**											
Immunotherapy											
Chen (2025) [98]	CT	SMI	L3	90	42.84	7.87	41.63	8.11	−1.21 (± 3.72)	−2.82%**	NR
Immunotherapy + targeted therapy											
Shigefuku 2024 [99]	CT	PMI	L3	213	5.00	4.10–6.30	4.91	NR	NR	−1.20%	0.06
**Colorectal cancer**											
Chemotherapy											
Okuno (2019) [100]	CT	SMI	L3	NR	51.20	10.60	50.60	10.70	NR	−1.17%**	0.033
Chemotherapy + targeted therapy											
Nozawa (2021) [101] (Conversion)	CT	SMI	L3	127	M: 38.8 F: 30.20	8.10 6.10	M: 42.20 F: 32.25	6.20 5.75	NR	+9.40%	NR
Nozawa (2021) [101] (NACT)	CT	SMI	L3	75	M: 41.70 F: 34.00	7.30 6.60	M: 41.20 F: 30.60	6.60 6.10	NR	−5.90%	NR
Nozawa (2021) [101] (Palliation)	CT	SMI	L3	118	M: 39.90 F: 28.00	7.10 4.90	M: 38.50 F: 26.60	5.00 6.70	NR	−3.70%	NR
Palle (2016) [102]	BIA	SMM	Whole body	27.60	M: 37.40 F: 25.20	2.50 2.70	M: 37.40 F: 25.00	2.80 3.20	NR	0.00%**	0.944 0.156
Blauwhoff-Buskermolen (2016) [21]	CT	SMA	L3	78	138.60	32.10	131.90	31.70	NR	−6.10%	< 0.01
Gallois (2020) [104]	CT	SMI	L3	60	41.00	8.80	39.20	8.090	NR	−4.39%**	NR
**Breast cancer**											
Chemotherapy											
Jang (2022) [105] (AC-T)	CT	SMI	L3	161	42.40	5.40	42.40	5.90	−0.22	0.00%**	0.83
Campbell (2007) [106]	DXA	SMM	Whole body	105	43.30	4.20	43.50	4.50	NR	+0.46%**	0.65
Jung (2020) [107]	BIA	SMM	Whole body	NR	39.41	4.89	39.42	5.15	NR	+0.03%**	0.187
Chemotherapy + immunotherapy (+ targeted therapy)											
Camilleri (2024) [108]	CT	SMI	L3	182	40.80	6.40	40.40	6.40	NR	−0.98%**	0.09
Chemotherapy + targeted therapy											
Zhang (2024) [109]	CT	PEMA	TH12	152	26.09	6.80	23.66	5.98	NR	−9,31%**	< 0.00
Rossi (2023) [110]	MRI	PMA	T4–T5	158	9.70	2.60	8.70	2.20	−1.41	−10.31%**	< 0.01
Karaca (2024) [111]	CT	PMA (mm^2^)	L3	NR	502.80	118.00	454.30	115.10	NR	−9.65%**	< 0.01
Amitani (2022) [18]	CT	SMI	L3	NR	46.50	7.60	46.30	8.00	NR	−0.43%**	NR
Rossi (2020) [113]	MRI	PMA	Pectoralis (sternal angle)	166.80	8.12	NR	7.03	NR	NR	−13.33%**	< 0.01
Mazzuca (2018) [114]	CT	SMI	L3	NR	39.20	31.60–52.90	39.20	31.60–52.90	NR	0.00%**	NR
**Lymphoma**											
Chemotherapy + targeted therapy											
Xiao (2016) [115]	CT	SMA	L3	NR	173.6	NR	168.8	NR	−4.8	−2.8%	NR

* Table 3 includes only studies reporting mean or median skeletal muscle mass values. Studies reporting solely the prevalence of low SMM are presented in Table S14.

** *Percentage change was calculated by the present authors.*

SD: standard deviation; CT: computerized tomography; MRI: magnetic resonance imaging; DXA: dual-energy X-ray absorptiometry; BIA: bioelectrical impedance analysis; SMI: skeletal muscle index, cm^2^/m^2^; SMM: skeletal muscle mass, kg; SMA: skeletal muscle area, cm^2^; PEMA: pectoralis muscle area, cm^2^; PMI: psoas muscle index, cm^2^/m^2^; PMA: psoas muscle area, cm^2^; LMV: lumbar muscle volume, cm^3^; SMI inclination: (SMI-change/SMI)/duration; M: male. F: female. NR: not reported.

In exploratory subgroup analyses by treatment type, the largest decline was observed in chemotherapy (45 studies; *n* = 5,169; SMC: –0.27, 95% CI: –0.32 to –0.22; *I*^2^ = 92%), followed by chemotherapy + targeted therapy (7 studies; *n* = 683; SMC: –0.23, 95% CI: –0.38 to –0.08; *I*^2^ = 91%) and immunotherapy (4 studies; *n* = 287; SMC: –0.15, 95% CI: –0.30 to 0.01; *I*^2^ = 63%). No loss of SMM with combined chemotherapy and immunotherapy was observed. By treatment setting, the largest decline was observed for palliative treatment (10 studies; *n* = 1,393; SMC: –0.31, 95% CI: –0.47 to –0.16; *I*^2^ = 95%).

The magnitude of SMM loss varied across cancer types, but differences were not statistically significant (*p* = 0.193). Still, overlapping confidence intervals together with high heterogeneity limit direct comparisons between individual groups.

The largest and most consistent reductions were observed among patients with pancreatic (6 studies; *n* = 828; SMC = –0.41, 95% CI: –0.63 to –0.19; *I*^2^ = 94%), urological (7 studies; *n* = 401; SMC = –0.30, 95% CI: –0.42 to –0.18; *I*^2^ = 66%) and lung cancer (6 studies; *n* = 552; SMC = –0.30, 95% CI: –0.54 to –0.06; *I*^2^ = 94%), corresponding to unweighted mean declines of –8%, –6%, and –5%, respectively.

No significant change in SMM was observed in breast cancer, colorectal cancer, or studies including multiple cancer types. Overall heterogeneity remained considerable, but exploratory sensitivity analyses did not alter the pooled estimates, except for the sex-stratified subgroup analysis.

Males (*n* = 438) had smaller SMM losses (SMC: –0.21, 95% CI: –0.42 to –0.01; *I*^2^ = 92%) than females (*n* = 263; SMC: –0.40, 95% CI: –0.69 to –0.12; *I*^2^ = 93%). All subgroup analyses are presented in Table 2 and Tables S6–S13.

Thirty-one studies (*n* = 5,376 patients) reported the prevalence or percentage of low SMM during treatment (Table S14). In total, 20 distinct definitions of low SMM were identified. The SMI measured by CT at the L3 was the dominant criterion used in 28 (90%) studies, typically expressed with sex-specific cut-offs between < 52–55 cm^2^/m^2^ for males and < 38–41 cm^2^/m^2^ for females. In 26 (84%) studies, the prevalence of low SMM increased during treatment, with the mean percentage rising from 43% to 51% (Table S14). Excluded studies on full-text screening are presented in Table S15.

## Discussion

Findings from our comprehensive systematic review and meta-analysis indicate that declines in SMM are frequently observed during systemic cancer treatment, particularly during chemotherapy (± targeted therapy), although effect sizes were generally small per Cohen’s thresholds. Among the 59 studies included in the meta-analysis, 78% showed a decline in SMM. However, substantial between-study heterogeneity suggests that the magnitude of change varies considerably across clinical contexts.

The wasting of SMM may have several possible mechanistic pathways. Cancer itself may directly and indirectly suppress muscle protein synthesis while increasing protein catabolism [29]. Tumor growth and systemic disease can create a chronic catabolic environment characterized by pro-inflammatory signaling, hormonal imbalance, altered metabolism, and energy deficit [3, 29]. Pro-inflammatory cytokines such as interleukin-6, interleukin-1β, tumor necrosis factor-α, and transforming growth factor-β promote proteolysis and inhibit muscle protein synthesis through activation of the ubiquitin, proteasome system and suppression of mTOR signaling [3, 30]. Chronic inflammation may also interact with hormonal changes such as reduced testosterone and insulin-like growth factor-1, to further impair anabolism [3, 30]. In a large cohort of patients with colorectal cancer (*n* = 2,470), the coexistence of low SMM and systemic inflammation more than doubled the risk of cancer-specific mortality (HR: 2.43, 95% CI: 1.79–3.29) [31]. These findings support the concept that cancer-related systemic inflammation may contribute to SMM loss, although the relative contribution of disease- versus treatment-related mechanisms cannot be disentangled in the included studies.

The magnitude of SMM loss differed markedly by treatment type. Across (*n* = 45) cohorts with patients receiving chemotherapy (*n* = 5,169), the pooled SMC was –0.27 (95% CI:–0.32 to –0.22), with consistent direction of change across studies, albeit considerable heterogeneity. These findings align closely with the review by Jang et al. [32], who synthesized 15 studies (*n* = 2,662) and reported a mean absolute reduction of 2.72 cm^2^/m^2^ (95% CI: 1.77–3.67) in SMI during chemotherapy treatment [32]. Our study extends these observations across a broader range of cancer types, treatment settings and sample sizes.

Across other treatment modalities, SMM loss was evident but varied in magnitude. Estimates for immunotherapy and combined treatment should be interpreted with caution due to the limited number of studies**.** The observed treatment differences likely reflect distinct biological and metabolic mechanisms. Cytotoxic chemotherapy, particularly platinum-based regimens, induces direct myotoxicity through mitochondrial dysfunction, oxidative stress, and activation of catabolic transcription factors, while concurrently suppressing anabolic pathways and increasing myostatin expression [3]. These molecular effects are further compounded by systemic inflammation, toxicities, and treatment-related symptoms such as nausea, fatigue and pain, which collectively can diminish nutrient intake and physical activity, and thus reinforcing a cycle of muscle disuse and atrophy [3, 33].

Immunotherapy could influence SMM though similar pathways as cytotoxic regimens through symptom burden, cytokine-driven inflammation, and physical inactivity rather than direct cellular injury [34]. Nonetheless, biological pathways underlying muscle depletion during immunotherapy remain incompletely understood and should be addressed through robust mechanistic and prospective longitudinal studies.

Our findings did not indicate a clear additional effect of targeted agents on SMM loss when administered in combination with chemotherapy. This interpretation aligns with Kakinuma et al. [35], in a study of patients with advanced non-small cell lung cancer (NSCLC) (*n* = 65). In their cohort, chemotherapy led to a 10% decline in SMI (from 44.8 to 40.4 cm^2^/m^2^), whereas targeted therapy treatment did not reduce SMI [35]. Sex differences in SMM loss may partly explain these findings. In our study, six of eight studies (75%) in chemotherapy + targeted therapy subgroups included breast cancer cohorts. In Jang et al. [32] (males, *n* = 823; females, *n* = 352), absolute declines in SMI were 1.6 times greater in males than in females (–4.52 vs. –2.86 cm^2^/m^2^) in sex-stratified sub-analyses. By contrast, in our review, eight sex-stratified studies showed larger relative losses in females (SMC: –0.40; *n* = 263) than in males (SMC: –0.21; *n* = 438). However, these findings are based on a limited number of studies and should be interpreted cautiously**.** Thus, further elucidation of absolute and relative changes stratified by sex and treatment type is warranted to conclude whether sex-based differences exist.

Across treatment settings, the greatest SMM loss was observed in patients in palliative treatment settings with SMC –0.31 (95% CI: –0.47 to –0.16). In agreement, a longitudinal cohort of (*n* = 3,075) community-dwelling older adults aged 70–79 years. Williams and colleagues [36] found that among the (*n* = 515) adults who developed cancer, the loss in SMM was most pronounced among people with metastatic disease, indicating that cancer stage and treatment setting amplify age-related SMM wasting [36]. Still, among the seven studies showing the most pronounced declines (SMC from –0.69 to –0.50), neoadjuvant [23, 37], adjuvant [38], palliative [39–41], and unclassified treatment [35] settings were present. This distribution suggests that SMM loss may occur across different treatment settings, although the magnitude of change varies substantially between studies**.**

### Clinical implications of SMM loss

Reduced overall and progression-free survival could, in part, be explained by reduced treatment tolerance in patients with SMM wasting, potentially reflecting a pharmacokinetic mismatch driven by current dosing practices. Most cytotoxic agents are dosed by body surface area, which does not account for lean versus fat mass distribution [1, 42]. Because anticancer drugs distribute primarily into metabolically active tissues, patients with low SMM and high body surface area may experience a higher relative dose per unit of lean tissue, predisposing them to toxicity and dose reductions [1, 42]. Conversely, individuals with preserved SMM have greater drug clearance and fewer adverse effects [43]. In the recent phase II Randomized LEANOX Trial, Assenat et al. [44] found that using an lean body mass-based oxaliplatin dose significantly reduced peripheral neurotoxicity and improved quality of life without affecting relapse-free and overall survival [44]. This indicates that SMM loss may contribute directly to treatment-related toxicity.

In healthy individuals around age 75, muscle strength declines by roughly 3–4% per year in men and 2–3% per year in women [46]. Studies evaluating both strength and SMM within the same cohorts further indicate that strength decreases at a rate two to five times greater than SMM [46]. Importantly, loss of muscle strength and power is a more consistent predictor of disability and mortality than loss of SMM in older adults [46, 47].

Still, prospective studies directly linking cancer-related SMM and strength loss to clinical outcomes remain scarce.

Physical exercise remains the most potent non-pharmacological strategy to preserve SMM and strength [45], and progressive resistance training can provide an anabolic stimulus in patients with cancer. A meta-analysis of 34 randomized trials demonstrated a mean gain of 0.85 kg (95% CI: 0.26–1.43) in lean body mass compared with controls [4, 48]. Yet, most studies have excluded older, malnourished, or patients with low physiological fitness – the individuals most vulnerable to muscle wasting but also those with the greatest potential for relative improvements in physical function and clinical outcomes [33]. Current evidence suggests that patients should aim for a protein intake of approximately 1.5 g/kg/day, or 15–20% of total caloric intake, to mitigate treatment-related SMM loss [49]. However, high-quality trials are required to confirm feasibility and efficacy of such multimodal interventions [33, 45].

### Strengths and limitations

To our knowledge, this meta-analysis represents the largest and most comprehensive synthesis to date of systemic treatment-related SMM loss across cancer types. By excluding studies involving surgery or radiotherapy, we provide a clearer picture of chemotherapy and immunotherapy-related changes in SMM. Most studies (76%) were eligible for quantitative synthesis, which enhanced statistical power and generalizability. No evidence of publication bias was detected.

Nonetheless, substantial heterogeneity persisted, likely reflecting variation in cancer types and treatment regimens. Accordingly, the pooled estimate should be interpreted as a summary of heterogeneous findings rather than a single generalizable effect**.** We recognize that observed changes in SMM are most likely influenced by factors such as disease progression, treatment-related toxicity, and nutritional status, and should not be interpreted as independent of these processes. In particular, the inclusion criteria of two available SMM assessments, which preclude a loss to follow-up evaluation, introduce selection and survivorship bias in retrospective studies. Accordingly, the risk-of-bias assessment focuses on the validity of estimating within-patient change while acknowledging the inherent limitations of observational study designs, including residual confounding and selection bias. In addition, variation in outcome definitions (e.g. SMI, SMA, and absolute SMM) represents a further source of heterogeneity, as these measures are not directly equivalent despite standardization.

Because analyses relied on study-level summary estimates, planned stratification by treatment agent, age, disease stage or number of cycles were not possible. Furthermore, 38% of studies did not report the interval between SMM assessments, limiting precise interpretation of the rate and timing of SMM loss. Finally, varying definitions of low SMM, primarily lacking functional measures [1], precluded evaluation of sarcopenia as this is defined by combined assessments of muscle strength and SMM [50].

## Conclusion

In summary, declines in SMM are frequently observed during systemic cancer treatment. However, substantial heterogeneity across studies indicates that the magnitude of change varies across clinical contexts, and findings should not be interpreted as a uniform effect**.** The extent varies across cancer types and treatment modalities, reflecting the interplay of biological, treatment-related, and behavioral factors. Integrating automated body composition analysis into routine imaging could enable early detection of clinically meaningful SMM wasting, inform treatment planning, and facilitate timely preventative interventions.

## Supplementary Material



## Data Availability

The data underlying this article are available in the article and in its online supplementary material. Any further information is available on request by contacting the corresponding author.
