# Supplementary material for: Change in skeletal muscle mass during systemic cancer treatment: a systematic review and meta-analysis
Source: Acta Oncol. 2026 May 27;65:45726. doi: 10.2340/1651-226X.2026.45726 (PMC13221715; doi:10.2340/1651-226X.2026.45726)
Supplement: Supplementary file 1 [file AO-65-45726-s1.pdf]

Supplementary material has been published as submitted. It has not been copyedited, or typeset by Acta Oncologica

Supplementary matarial

Table S1: PRISMA checklist
Table S2: Search strategy
Table S3: Eligibility criteria
Table S4: Funding, data source and patient setting
Table S5: Quality Assessment Tool for Observational Cohort and Cross-Sectional Studies published by the National Heart Lung and Blood Institute
Figure S1: Funnel plot
Table S6: Meta-analyses of the change of skeletal muscle mass during treatment of pancreatic cancer
Table S7: Meta-analyses of the change of skeletal muscle mass during treatment of urological cancer
Table S8: Meta-analyses of the change of skeletal muscle mass during treatment of lung cancer
Table S9: Meta-analyses of the change of skeletal muscle mass during treatment of gastric, esophagastric or esophageal cancers
Table S10: Meta-analyses of the change of skeletal muscle mass during treatment of ovarian cancer
Table S11: Meta-analyses of the change of skeletal muscle mass during treatment of studies including cohorts with multiple cancers
Table S12: Meta-analyses of the change of skeletal muscle mass during treatment of colorectal cancer
Table S13: Meta-analyses of the change of skeletal muscle mass during treatment of breast cancer
Table S14: Prevalence of low skeletal muscle mass during systemic cancer treatment
Table S15: Excluded studies on full-text screening

Table S1: PRISMA checklist

Section and Topic	Item #	Checklist item	Location where item is reported
TITLE			
Title	1	Identify the report as a systematic review.	1
ABSTRACT			
Abstract	2	See the PRISMA 2020 for Abstracts checklist.	1
INTRODUCTION			
Rationale	3	Describe the rationale for the review in the context of existing knowledge.	1-2
Objectives	4	Provide an explicit statement of the objective(s) or question(s) the review addresses.	2
METHODS			
Eligibility criteria	5	Specify the inclusion and exclusion criteria for the review and how studies were grouped for the syntheses.	2<br>Table S3
Information sources	6	Specify all databases, registers, websites, organisations, reference lists and other sources searched or consulted to identify studies. Specify the date when each source was last searched or consulted.	2
Search strategy	7	Present the full search strategies for all databases, registers and websites, including any filters and limits used.	Table S2
Selection process	8	Specify the methods used to decide whether a study met the inclusion criteria of the review, including how many reviewers screened each record and each report retrieved, whether they worked independently, and if applicable, details of automation tools used in the process.	2
Data collection process	9	Specify the methods used to collect data from reports, including how many reviewers collected data from each report, whether they worked independently, any processes for obtaining or confirming data from study investigators, and if applicable, details of automation tools used in the process.	2
Data items	10a	List and define all outcomes for which data were sought. Specify whether all results that were compatible with each outcome domain in each study were sought (e.g. for all measures, time points, analyses), and if not, the methods used to decide which results to collect.	2-3
	10b	List and define all other variables for which data were sought (e.g. participant and intervention characteristics, funding sources). Describe any assumptions made about any missing or unclear information.	2-3
Study risk of bias assessment	11	Specify the methods used to assess risk of bias in the included studies, including details of the tool(s) used, how many reviewers assessed each study and whether they worked independently, and if applicable, details of automation tools used in the process.	2-3
Effect measures	12	Specify for each outcome the effect measure(s) (e.g. risk ratio, mean difference) used in the synthesis or presentation of results.	2-3
Synthesis methods	13a	Describe the processes used to decide which studies were eligible for each synthesis (e.g. tabulating the study intervention characteristics and comparing against the planned groups for each synthesis (item #5)).	3
	13b	Describe any methods required to prepare the data for presentation or synthesis, such as handling of missing summary statistics, or data conversions.	3
	13c	Describe any methods used to tabulate or visually display results of individual studies and syntheses.	3
	13d	Describe any methods used to synthesize results and provide a rationale for the choice(s). If meta-analysis was performed, describe the model(s), method(s) to identify the presence and extent of statistical heterogeneity, and software package(s) used.	3
	13e	Describe any methods used to explore possible causes of heterogeneity among study results (e.g. subgroup analysis, meta-regression).	3
	13f	Describe any sensitivity analyses conducted to assess robustness of the synthesized results.	3
Reporting bias assessment	14	Describe any methods used to assess risk of bias due to missing results in a synthesis (arising from reporting biases).	3

Certainty assessment	15	Describe any methods used to assess certainty (or confidence) in the body of evidence for an outcome.	-
RESULTS			
Study selection	16a	Describe the results of the search and selection process, from the number of records identified in the search to the number of studies included in the review, ideally using a flow diagram.	3-4, Figure 1
	16b	Cite studies that might appear to meet the inclusion criteria, but which were excluded, and explain why they were excluded.	Table S15
Study characteristics	17	Cite each included study and present its characteristics.	Table 1
Risk of bias in studies	18	Present assessments of risk of bias for each included study.	Table S5
Results of individual studies	19	For all outcomes, present, for each study: (a) summary statistics for each group (where appropriate) and (b) an effect estimate and its precision (e.g. confidence/credible interval), ideally using structured tables or plots.	3-4
Results of syntheses	20a	For each synthesis, briefly summarise the characteristics and risk of bias among contributing studies.	3-4
	20b	Present results of all statistical syntheses conducted. If meta-analysis was done, present for each the summary estimate and its precision (e.g. confidence/credible interval) and measures of statistical heterogeneity. If comparing groups, describe the direction of the effect.	3-4 Table 2, Figure 2, Table S6-13
	20c	Present results of all investigations of possible causes of heterogeneity among study results.	3-4, Table 2, Figure 2, Table S6-13
	20d	Present results of all sensitivity analyses conducted to assess the robustness of the synthesized results.	3-4 Table 2, Figure 2, Table S6-13
Reporting biases	21	Present assessments of risk of bias due to missing results (arising from reporting biases) for each synthesis assessed.	-
Certainty of evidence	22	Present assessments of certainty (or confidence) in the body of evidence for each outcome assessed.	3-4 Table 2, Figure 2, Table S6-13
DISCUSSION			
Discussion	23a	Provide a general interpretation of the results in the context of other evidence.	4-14
	23b	Discuss any limitations of the evidence included in the review.	13-14
	23c	Discuss any limitations of the review processes used.	13-14
	23d	Discuss implications of the results for practice, policy, and future research.	14
OTHER INFORMATION			
Registration and protocol	24a	Provide registration information for the review, including register name and registration number, or state that the review was not registered.	2
	24b	Indicate where the review protocol can be accessed, or state that a protocol was not prepared.	2
	24c	Describe and explain any amendments to information provided at registration or in the protocol.	14
Support	25	Describe sources of financial or non-financial support for the review, and the role of the funders or sponsors in the review.	14
Competing interests	26	Declare any competing interests of review authors.	14
Availability of data, code and other materials	27	Report which of the following are publicly available and where they can be found: template data collection forms; data extracted from included studies; data used for all analyses; analytic code; any other materials used in the review.	14

Table S2: Search strategy

PubMed	Web of Science	Embase
((((((("cancer survivors"[MeSH Terms]) OR (cancer survivors[Title/Abstract])) OR ("late effect*"[Title/Abstract])) OR (late-effect*[Title/Abstract])) OR ((((chemotherapy[MeSH Terms]) OR (chemotherap*[Title/Abstract])) OR (chemotherapy toxicity[Title/Abstract])) OR (late toxicit*[Title/Abstract]))) OR (((immunotherapy[MeSH Terms])) OR (immunotherap*[Title/Abstract])))<br><br>AND<br><br>(((((((((sarcopenia[MeSH Terms]) OR (sarcopen*[Title/Abstract])) OR (muscle depletion[Title/Abstract])) OR (skeletal muscle index[Title/Abstract])) OR (total muscle area[Title/Abstract])) OR (psoas muscle[Title/Abstract])) OR (cachexia[Title/Abstract])) OR (muscle change[Title/Abstract])) OR (muscle mass[Title/Abstract])))<br><br>AND<br><br>(((malignant neoplasm[MeSH Terms]) OR (malignant neoplasm*[Title/Abstract])) OR (malignanc*[Title/Abstract]))	((TS=("Cancer Survivor" OR "Cancer Survivors" OR survivor OR survivors OR survival OR late-effect OR late-effects OR "late effect" OR "late effects" OR "chemotherapy toxicity" OR "late toxicities" OR "late toxicity" OR immunotherapy OR immunotherap*))<br><br>AND<br><br>(TS=(sarcopenia OR sarcopenic OR "muscular atrophy" OR "muscle atrophy" OR "muscle wasting" OR cachexia OR "muscle failure" OR "muscle depletion" OR "skeletal muscle index" OR "total muscle area" OR "Psoas muscle" OR "muscle change" OR "muscle mass"))<br><br>AND<br><br>(TS=(Cancer OR cancers OR neoplasm OR neoplasms OR malignancy OR malignancies OR tumor OR tumors OR tumour OR tumours))	1 exp cancer survivor/<br>2 cancer surviv*.ti,ab,kw.<br>3 late effect*.ti,ab,kw.<br>4 late-effect*.ti,ab,kw.<br>5 1 or 2 or 3 or 4<br>6 exp chemotherapy/<br>7 chemotherap*.ti,ab,kw.<br>8 chemotherapy toxicity.ti,ab,kw.<br>9 late toxicit*.ti,ab,kw.<br>10 6 or 7 or 8 or 9<br>11 exp immunotherapy/<br>12 immunotherap*.ti,ab,kw.<br>13 11 or 12<br>14 5 or 10 or 13<br>15 exp sarcopenia/<br>16 sarcopen*.ti,ab,kw.<br>17 muscle depletion.ti,ab,kw.<br>18 skeletal muscle index.ti,ab,kw.<br>19 total muscle area.ti,ab,kw.<br>20 psoas muscle.ti,ab,kw.<br>21 cachexia.ti,ab,kw.<br>22 muscle change.ti,ab,kw.<br>23 muscle mass.ti,ab,kw.<br>24 15 or 16 or 17 or 18 or 19 or 20 or 21 or 22 or 23<br>25 exp malignant neoplasm/<br>26 malignant neoplasm*.ti,ab,kw.<br>27 malignanc*.ti,ab,kw.<br>28 25 or 26 or 27<br>29 14 and 24 and 28

Table S3: Eligibility criteria

Inclusion	Exclusion
Studies published in English, German, Scandinavian or other languages where professional translation is not required	Non-English, -German or -Scandinavian papers
Papers published in peer-reviewed journal	Grey literature (e.g. dissertations, conference abstracts)
Observational study design (not systematic reviews, not interventions study i.e., exercise or nutrition)	Not an observational study design (systematic reviews, interventions study i.e. exercise or nutrition)
Adult cancer patients > 18 years	Not an adult cancer population
Uses CT, MRI, DXA, bioimpedance to measure or estimate skeletal muscle mass and report these outcomes	No use of specific measurements for muscle mass (and report these outcomes, i.e., CT, MRI, DXA, bioimpedance)
Including at least two measurements of skeletal muscle mass i.e. pre- and post-chemotherapy or immunotherapy or a combination and report these outcomes	Not including at least two measurements of skeletal muscle mass pre- and post- chemotherapy, immunotherapy, or a combination and report these outcomes

Table S4: Funding, data source and patient setting

Study	Funding	Data Source	Patient setting
Shimura 2023	Reported	Medical records	Hospital
Jin 202	Reported	Medical records	Teaching hospital
Griffin 2019	NR	Prospectively maintained database	University hospital
Lee 2024	NR	Medical records	Hospital
Rollins 2016	Reported	Electronic records of the Nottingham Information System	University hospital
Aberle 2025	NR	Medical records	Hospital
Davis 2025	NR	Medical records	Hospital
Uemura 2020	NR	Medical records	University hospital
Lee 2019	NR	NR	Academic hospital
Miyake 2018	Reported	Medical records	Hospital
MacDonald 2024	NR	Medical records	Hospital
Rimar 2018	None	NR	Single institution
Lyon 2019	NR	Cystectomy registry	Hospital
Takai 2021	NR	NR	Hospital
Mitsui 2018	None	NR	University Hospital
Semerad 2022	NR	Medical records	Hospital
Buxton 2024	NR	Medical records	Hospital
Goncalves 2018	Reported	Clinical chart review	Hospital
Stene 2015	NR	Medical records	Hospital
Kazemi-Bajestani 2019	NR	NR	Hospital
Nattenmüller 2017	None	Medical records	Hospital
Kidd 2024	Reported	Medical records	Hospital
Kakinuma 2018	NR	Medical records	Hospital
Khan 2023	None	Medical records	Hospital
Chaunzwa 2024	NR	NR	Hospital
Cortellini 2018	NR	Medical records	Hospital
Sato 2024	Reported	Medical records	Hospital
Juez 2024	Reported	Medical records	Hospital
Li 2024	Reported	Medical records	Hospital
Mirkin 2017	NR	Medical records	Hospital
Matsuura 2020	NR	Medical records	Hospital
Horii 2022	NR	Medical records	Hospital
Sugiyama 2018	None	Medical records	Hospital
Park 2020	NR	Medical records	Hospital
Palmela 2017	NR	Medical records	Hospital
Boer 2020	None	Specific database	NR
Rinninella 2021	None	Medical records	Hospital
Fujihata 2021	NR	Medical record	Hospital
Dijksterhuis 2019	Reported	NR	Medical center
Hacker 2022	None	Medical record	Hospital
Awad 2012	None	NR	Regional
Onishi 2024	None	Database	Hospital
Harada 2025	Reported	Medical record	Hospital
Yip 2014	Reported	NR	NR
Miyata 2017	NR	Medical records	Hospital
Ishida 2019	None	NR	University hospital
Zhao 2024	NR	Medical record	Hospital
Ying 2025	Reported	Medical record	Hospital
Wood 2023	NR	Medical records	Hospital
Ubachs 2020	Reported	NR	Hospital
Yoshino 2020	NR	Medical records	Hospital
Del Grande 2021	NR	Medical records	Hospital
Van der Zanden 2021	None	Medical records	Multicenter hospital

Toama 2022	Supported by NIH grants	NR	NR
Loosen 2021	Reported	Medical records	Hospital
Roeland 2021	Reported	NR	Cancer center
Oflazoglu 2020	NR	NR	Oncology clinic and training hospital
Daly 2017	Science Foundation Ireland	Medical records	Hospital
Chen 2025	Reported	Medical records	Hospital
Shigefuku 2024	Reported	Medical records	Hospital
Okuno 2019	Reported	Medical records	Hospital
Nozawa 2021	Reported	Medical records	Hospital
Palle 2016	Reported	Medical records	Hospital
Huemer 2019	Reported	NR	Tertiary-care cancer center
Blauwhoff-Buskermolen 2016	Reported	Medical records	Medical center
Gallois 2021	Reported	Computer platform	8 Medical centers
Jang 2022	Reported	Medical records	Hospital
Campbell 2007	Reported	Medical records	Hospital
Jung 2020	NR	Medical records	Hospital
Camilleri 2024	None	Medical records	Hospital
Zhang 2024	None	Medical records	Hospital
Rossi 2023	NR	Medical records	Hospital
Karaca 2024	NR	Medical records	Hospital
Amitani 2022	None	Medical records	Hospital
Lee 2021	Reported	Medical records	Hospital
Rossi 2020	None	NR	University hospital
Mazzuca 2018	None	NR	Hospital
Xiao 2016	Reported	Patient records + Veteran's Health Administration Central Cancer Registry	50 treatment sites

Table S5: Quality Assessment Tool for Observational Cohort and Cross-Sectional Studies published by the National Heart Lung and Blood Institute

1. Research question/objective clearly stated?	2. Study population clearly specified?	3. Participation rate of eligible persons at least 50%?	4. All subjects selected/recruited from same/similar populations? Inclusion/exclusion criteria applied uniformly to all participants?	5. Sample size justification, power description, or variance and effect estimates provided?	6. Re: analyses - exposure(s) of interest measured prior to the outcome(s) being measured?	7. Sufficient timeframe to see an association between exposure and outcome?
8. For exposures that can vary in amount or level, did the study examine different levels of the exposure as related to the outcome?	9. Exposure measures (independent variables) clearly defined, valid, reliable, and implemented consistently across all participants?	10. Was the exposure(s) assessed more than once over time?	11. Outcome measures (dependent variables) clearly defined, valid, reliable, and implemented consistently across all participants?	12. Were the outcome assessors blinded to the exposure status of participants?	13. Was loss to follow-up after baseline 20% or less?	14. Key potential confounding variables measured/adjusted statistically for impact on relationship between exposure(s) and outcome(s)?

	1	2	3	4	5	6	7	8	9	10	11	12	13	14	Yes (%)*
Shimura 2023	√	√	√	√	/	√	√	√	√	√	√	√	/	/	100
Jin 2022	√	√	√	÷	/	√	√	÷	√	√	√	ǁ	/	/	73
Griffin 2019	√	÷	÷	√	/	√	÷	÷	√	√	√	ǁ	/	/	55
Lee 2024	√	√	√	√	/	√	√	√	√	√	√	√	/	/	100
Rollins 2016	√	√	√	√	/	√	√	÷	÷	√	√	ǁ	/	/	73
Aberle 2025	√	√	√	√	/	√	√	√	√	√	√	ǁ	/	/	91
Davis 2025	√	√	√	√	/	√	√	√	√	√	√	ǁ	/	/	91
Uemura 2020	√	√	√	√	/	√	√	÷	√	√	√	ǁ	/	/	82
Lee 2019	√	÷	√	√	/	√	√	√	÷	√	√	ǁ	/	/	73
Miyake 2018	√	÷	√	√	/	√	√	√	√	√	√	ǁ	/	/	82
MacDonald 2024	√	√	√	√	/	√	√	√	√	√	√	ǁ	/	/	91
Rimar 2018	√	√	√	√	/	√	√	÷	÷	√	√	ǁ	/	/	73
Lyon 2019	√	÷	√	√	/	√	√	÷	÷	√	√	ǁ	/	/	64
Takai 2021	√	÷	√	√	/	√	√	√	√	√	√	ǁ	/	/	82
Mitsui 2019	÷	÷	√	√	/	√	√	÷	÷	√	√	ǁ	/	/	55
Semerad 2022	√	√	√	√	/	√	√	÷	√	√	√	ǁ	/	/	82
Buxton 2024	√	√	√	√	/	√	√	√	√	√	√	√	/	/	100
Goncalves 2018	√	÷	√	√	/	√	√	÷	√	√	√	√	/	/	82

	1	2	3	4	5	6	7	8	9	10	11	12	13	14	Yes (%)*
Stene 2015	√	÷	√	√	/	√	√	÷	√	√	√	ǁ	/	/	82
Kazemi-Bajestani 2019	√	÷	√	÷	/	√	√	√	÷	√	√	√	/	/	73
Nattenmüller 2017	√	√	√	√	/	√	√	÷	√	√	√	√	/	/	82
Kidd 2024	√	√	√	√	/	√	√	√	√	√	√	ǁ	/	/	91
Kakinuma 2018	√	÷	√	÷	/	√	√	÷	√	√	√	√	/	/	73
Khan 2023	√	√	√	√	/	√	√	√	√	√	√	ǁ	/	/	100
Chaunzwa 2024	√	÷	√	√	/	√	√	√	√	√	√	ǁ	/	/	73
Cortellini 2018	√	√	√	√	/	√	√	÷	÷	√	√	ǁ	/	/	73
Sato 2024	√	√	√	√	/	√	√	√	√	√	√	√	/	/	100
Juez 2024	√	√	√	√	/	√	ǁ	√	√	√	√	ǁ	/	/	91
Li 2024	√	√	√	√	/	√	√	√	√	√	√	ǁ	/	/	100
Mirkin 2017	√	÷	√	√	/	√	√	÷	√	√	√	√	/	/	82
Matsuura 2020	√	÷	√	√	/	√	√	÷	√	√	√	√	/	/	82
Horii 2022	√	√	√	√	/	√	√	÷	÷	√	√	÷	/	/	73
Sugiyama 2018	√	√	√	√	/	√	√	÷	√	√	√	ǁ	/	/	82
Park 2020	√	÷	√	√	/	√	√	÷	√	√	√	ǁ	/	/	73
Palmela 2017	√	÷	√	√	/	√	√	÷	÷	√	√	ǁ	/	/	73
Boer 2020	√	÷	√	√	/	√	√	÷	√	√	√	ǁ	/	/	73
Rinninella 2021	√	√	√	√	/	√	√	÷	÷	√	√	√	/	/	91
Fujihata 2021	√	√	√	√	/	√	√	√	√	√	√	√	/	/	100
Dijksterhuis 2019	÷	÷	√	√	/	√	√	÷	√	√	√	ǁ	/	/	82
Hacker 2022	√	√	√	√	/	√	√	√	√	√	√	√	/	/	100
Awad 2012	√	√	√	√	/	√	√	÷	√	√	√	√	/	/	91
Onishi 2024	√	√	√	√	/	√	√	√	√	√	√	ǁ	/	/	91
Harada 2025	√	√	√	√	/	√	√	√	√	√	√	ǁ	/	/	91
Yip 2014	√	√	√	√	/	√	√	÷	÷	√	√	√	/	/	73
Miyata 2017	√	√	√	√	/	√	√	√	√	√	√	ǁ	/	/	91
Ishida 2019	√	√	√	√	/	√	√	√	√	√	√	√	/	/	91
Zhao 2024	√	√	√	√	/	√	√	÷	√	√	√	ǁ	/	/	91
Ying 2025	√	√	÷	√	/	√	√	√	√	√	√	ǁ	/	/	82
Wood 2023	√	√	√	√	/	√	√	√	√	√	√	ǁ	/	/	100
Ubachs 2020	√	√	√	√	/	√	√	÷	√	√	√	ǁ	/	/	91
Yoshino 2020	√	÷	√	√	/	√	√	÷	÷	√	√	ǁ	/	/	73
Del Grande 2021	√	√	√	√	/	√	√	÷	√	√	√	ǁ	/	/	91
Van der Zanden 2021	√	÷	√	√	/	√	√	÷	÷	√	√	ǁ	/	/	73
Toama 2022	√	√	√	√	/	√	√	√	√	√	√	ǁ	/	/	100

	1	2	3	4	5	6	7	8	9	10	11	12	13	14	Yes (%)*
Loosen 2021	√	√	√	√	/	√	√	÷	√	√	√	√	/	/	91
Roeland 2021	√	√	√	√	/	√	√	÷	√	√	√	√	/	/	91
Oflazoglu 2020	√	√	√	√	/	√	√	÷	÷	√	√	√	/	/	82
Daly 2017	√	÷	√	√	/	√	√	÷	√	√	√	‖	/	/	73
Chen 2025	√	√	√	√	/	√	√	√	√	√	√	‖	/	/	100
Shigefuku 2024	√	√	√	‖	/	√	√	√	√	√	√	‖	/	/	91
Okuno 2019	√	÷	√	√	/	√	‖	÷	÷	√	√	‖	/	/	73
Nozawa 2021	√	√	√	√	/	√	√	÷	÷	√	√	‖	/	/	73
Palle 2016	√	÷	√	√	/	√	‖	÷	÷	√	√	‖	/	/	64
Huemer 2019	√	√	√	√	/	√	√	÷	√	√	√	√	/	/	91
Blauwhoff-Buskermolen 2016	√	√	√	√	/	√	√	÷	÷	√	√	‖	/	/	82
Gallois 2021	√	√	√	√	/	√	√	÷	√	√	√	‖	/	/	91
Jang 2022	√	√	√	√	/	√	√	√	√	√	√	‖	/	/	100
Campbell 2007	√	√	√	√	/	√	√	√	√	√	√	‖	/	/	100
Jung 2020	√	√	√	√	/	√	√	÷	√	√	√	√	/	/	91
Camilleri 2024	√	√	√	√	/	√	√	√	√	√	√	‖	√	/	100
Zhang 2024	√	√	√	√	/	√	√	÷	√	√	√	‖	/	/	91
Rossi 2023	√	√	√	√	/	√	√	÷	√	√	√	‖	/	/	91
Karaca 2024	√	√	√	√	/	√	‖	√	√	√	√	‖	/	/	91
Amitani 2022	√	√	√	√	/	√	√	√	√	√	√	‖	/	/	100
Lee 2021	√	√	√	√	/	√	√	÷	√	√	√	√	/	/	91
Rossi 2020	√	√	√	√	/	√	√	÷	÷	√	√	‖	/	/	82
Mazzuca 2018	√	÷	√	√	/	√	√	÷	÷	√	√	‖	/	/	73
Xiao 2016	√	√	√	√	/	√	√	÷	√	√	√	√	/	/	91
√ Yes, ÷ = No, ‖ = Not Reported, / = Not Applicable, * excluding item 5, 13 and 14 (see methods section). In total (median)															91

Figure S1: Funnel plot

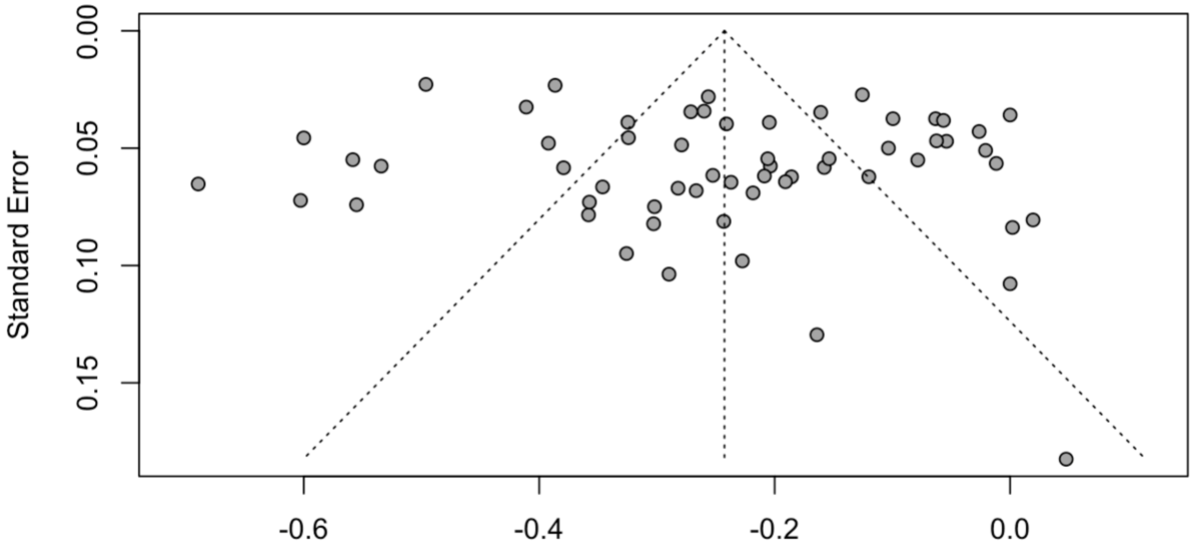

Table S6-S13

Table S6: Meta-analyses of the change in skeletal muscle mass during treatment of pancreatic cancer

Pancreatic cancer	*N*	SMC (95% CI)	Comparisons	I^2	*P*-value
Primary analysis	828	-0.41 (-0.63 to -0.19)	5 [1-6]	94%	
Subgroup analyses [a]					
Treatment type					
Chemotherapy	828	-0.41 (-0.63 to -0.19)	5 [1-6]	94%	
Treatment setting	-	-	-	-	
Neoadjuvant	231	-0.33 (-0.91 to 0.25)	3 [1,4,5]	95%	
Palliative	597	-0.49 (-0.99 to 0.02)	3 [2,3,6]	93%	
Assesment tool					
CT SMI (cm/m^2)	771	-0.25 (-0.58 to -0.13)	4 [1-5]	94%	
Sensitivity analyses					
Estimated means [b]	329	-0.32 (-0.62 to to -0.01)	4 [1,3-5]	92%	
Correlation coefficient estimation [d]	828	-0.36 (-0.59 to -0.13)	5 [1-6]	78%	
Funnel plot assymetry					

Abbreviations: N, number with complete data; SMC, Standardized mean change; CI, Confidence interval; I^2; Heterogeneity; Chemo, Chemotherapy; TT, Targeted therapy; IO, Immunotherapy; CT, Computed tomography; CM, Centimeter; SMI, Skeletal Muscle Index; SMA, Skeletal Muscle Area; PMI, Psoas (or Pectoralis) Muscle Index; PMA, Psoas (or Pectoralis) Muscle Area; BIA, Bioimpedance anaysis; Kg., Kilograms.

[a] Only subgroups with ≥2 studies are presented.

[b] Estimated means: Sensitivity analysis excluding studies where means and SDs were derived from medians/IQRs using Wan et al.'s method.

[c] Non-small studies: Sensitivity analysis excluding studies with n ≤ 100.

[d] Correlation coefficient estimation: Sensitivity analysis with imputed correlation coefficient (r = 0.5)

Table S7: Meta-analyses of the change in skeletal muscle mass during treatment of urological cancer

Urological cancer	*N*	SMC (95% CI)	Comparisons	I^2	*P*-value
Primary analysis	401	-0.30 (-0.42 to -0.18)	7 [7-13]	65%	
Subgroup analyses [a]					
Treatment type					
Chemotherapy	401	-0.30 (-0.42 to -0.18)	7 [7-13]	70%	
Treatment setting					
Neoadjuvant	110	-0.23 (-0.40 to -0.06)	3 [8,12,13]	0%	
Not reported	255	-0.29 (-0.43 to -0.16)	3 [9-11]	0%	
Assesment tool					
CT SMI (cm/m^2)	328	-0.32 (-0.51 to -0.14)	5 [7-9,12,13]	74%	
Sensitivity analyses					
Estimated means [b]	155	-0.34 (-0.59 to -0.10)	4 [7,8,10,12]	78%	
Correlation coefficient estimation [d]	416	-0.33 (-0.47 to -0.20)	7 [7-13]	43%	
Funnel plot assymetry					

Abbreviations: N, number with complete data; SMC, Standardized mean change; CI, Confidence interval; I^2; Heterogeneity; Chemo, Chemotherapy; TT, Targeted therapy; IO, Immunotherapy; CT, Computed tomography; CM, Centimeter; SMI, Skeletal Muscle Index; SMA, Skeletal Muscle Area; PMI, Psoas (or Pectoralis) Muscle Index; PMA, Psoas (or Pectoralis) Muscle Area; BIA, Bioimpedance anaysis; Kg., Kilograms.

[a] Only subgroups with ≥2 studies are presented.

[b] Estimated means: Sensitivity analysis excluding studies where means and SDs were derived from medians/IQRs using Wan et al.'s method.

[c] Non-small studies: Sensitivity analysis excluding studies with n ≤ 100.

[d] Correlation coefficient estimation: Sensitivity analysis with imputed correlation coefficient (r = 0.5)

Table S8: Meta-analyses of the change in skeletal muscle mass during treatment of lung cancer

Lung cancer	*N*	SMC (95% CI)	Comparisons	I^2	*P*-value
Primary analysis	552	-0.30 (-0.54 to -0.06)	6 [14-19]	94%	
Subgroup analyses [a]					
Treatment type					
Chemotherapy	464	-0.36 (-0.61 to -0.10)	5 [14,16-19]	93%	
Treatment setting					
Palliative	138	-0.14 (-1.76 to 1.48)	2 [14,15]	88%	
Not reported	336	-0.32 (-0.92 to 0.28)	3 [16-18]	94%	
Assesment tool					
CT SMI (cm/m^2)	424	-0.24 (-0.64 to 0.16)	4 [15-18]	93%	
Sensitivity analyses					
Estimated means [b]	382	-0.26 (-0.65 to 0.14)	4 [14-17]	93%	
Correlation coefficient estimation [d]	552	-0.29 (-0.53 to -0.05)	6 [14-19]	76%	
Funnel plot assymetry					

Abbreviations: N, number with complete data; SMC, Standardized mean change; CI, Confidence interval; I^2; Heterogeneity; Chemo, Chemotherapy; TT, Targeted therapy; IO, Immunotherapy; CT, Computed tomography; CM, Centimeter; SMI, Skeletal Muscle Index; SMA, Skeletal Muscle Area; PMI, Psoas (or Pectoralis) Muscle Index; PMA, Psoas (or Pectoralis) Muscle Area; BIA, Bioimpedance anaysis; Kg., Kilograms.

[a] Only subgroups with ≥2 studies are presented.

[b] Estimated means: Sensitivity analysis excluding studies where means and SDs were derived from medians/IQRs using Wan et al.'s method.

[c] Non-small studies: Sensitivity analysis excluding studies with n ≤ 100.

[d] Correlation coefficient estimation: Sensitivity analysis with imputed correlation coefficient (r = 0.5)

Table S9: Meta-analyses of the change in skeletal muscle mass during treatment of gastric, esophagastric and esophageal cancers

Gastric, esophagastric and esophageal cancers	*N*	SMC (95% CI)	Comparisons	I^2	*P*-value
Primary analysis	2113	-0.23 (-0.29 to -0.16)	19 [20-37]	87%	
Subgroup analyses [a]					
Treatment type					
Chemotherapy	2028	-0.24 (-0.30 to -0.17)	18 [20-30,32-37]	87%	
Treatment setting					
Neoadjuvant	1511	-0.19 (-0.24 to -0.13)	15 [20,21,23-31,33,35,37]	71%	
Palliative	484	-0.37 (-0.87 to 0.13)	3 [22,34,36]	95%	
Assesment tool					
CT SMI (cm/m^2)	1728	-0.24 (-0.32 to -0.16)	14 [20-22,24,25,29-34,36,37]	88%	
CT PMI (cm/m^2)	718	-0.22 (-0.55 to 0.11)	3 [27,28,35]	80%	
Sensitivity analyses					
Estimated means [b]	1333	-0.22 (-0.28 to -0.16)	12 [21-23,25-27,29,30,32,36,37]	72%	
Non-small trials [c]	1112	-0.27 (-0.42 to -0.12)	7 [22,24,25,27,30,32,34]	94%	
Correlation coefficient estimation [d]	2113	-0.22 (-0.29 to -0.16)	19 [20-37]	59%	
By sex (male)	215	-0.40 (-1.06 to 0.25)	3 [32,34,36]	96%	
By sex (female)	101	-0.71 (1.64 to 0.21)	3 [32,34,36]	94%	
Funnel plot assymetry					0.718

Abbreviations: N, number with complete data; SMC, Standardized mean change; CI, Confidence interval; I^2; Heterogeneity; Chemo, Chemotherapy; TT, Targeted therapy; IO, Immunotherapy; CT, Computed tomography; CM, Centimeter; SMI, Skeletal Muscle Index; SMA, Skeletal Muscle Area; PMI, Psoas (or Pectoralis) Muscle Index; PMA, Psoas (or Pectoralis) Muscle Area; BIA, Bioimpedance anaysis; Kg., Kilograms.

[a] Only subgroups with ≥2 studies are presented.

[b] Estimated means: Sensitivity analysis excluding studies where means and SDs were derived from medians/IQRs using Wan et al.'s method.

[c] Non-small studies: Sensitivity analysis excluding studies with n ≤ 100.

[d] Correlation coefficient estimation: Sensitivity analysis with imputed correlation coefficient (r = 0.5)

Table S10: Meta-analyses of the change in skeletal muscle mass during treatment of ovarian cancer

Ovarian cancer	*N*	SMC (95% CI)	Comparisons	I^2	*P*-value
Primary analysis	546	-0.21 (-0.45 to 0.02)	4 [38-41]	92%	
Subgroup analyses [a]					
Treatment type					
Chemotherapy	546	-0.21 (-0.45 to 0.02)	4 [38-41]	92%	
Treatment setting					
Neoadjuvant	546	-0.21 (-0.45 to 0.02)	4 [38-41]	92%	
Assesment tool					
CT SMI (cm/m^2)	486	-0.24 (-0.65 to -0.17)	3 [38,39,41]	94%	
Sensitivity analyses					
Estimated means [b]	386	-0.17 (-1.49 to 1.15)	2 [39,41]	94%	
Non-small trials [c]	486	-0.24 (-0.65 to -0.17)	3 [38,39,41]	94%	
Correlation coefficient estimation [d]	546	-0.22 (-0.45 to 0.01)	4 [38-41]	70%	
Funnel plot assymetry					-

Abbreviations: N, number with complete data; SMC, Standardized mean change; CI, Confidence interval; I^2; Heterogeneity; Chemo, Chemotherapy; TT, Targeted therapy; IO, Immunotherapy; CT, Computed tomography; CM, Centimeter; SMI, Skeletal Muscle Index; SMA, Skeletal Muscle Area; PMI, Psoas (or Pectoralis) Muscle Index; PMA, Psoas (or Pectoralis) Muscle Area; BIA, Bioimpedance anaysis; Kg., Kilograms.

[a] Only subgroups with ≥2 studies are presented.

[b] Estimated means: Sensitivity analysis excluding studies where means and SDs were derived from medians/IQRs using Wan et al.'s method.

[c] Non-small studies: Sensitivity analysis excluding studies with n ≤ 100.

[d] Correlation coefficient estimation: Sensitivity analysis with imputed correlation coefficient (r = 0.5)

Table S11: Meta-analyses of the change in skeletal muscle mass during treatment of studies combining multiple diagnoses

Studies combining multiple cancers	*N*	SMC (95% CI)	Comparisons	I^2	*P*-value
Primary analysis	567	-0.21 (-0.72 to 0.30)	3 [42-44]	92%	
Subgroup analyses [a]					
Treatment type	-	-	-	-	
Treatment setting					
Not reported	567	-0.21 (-0.72 to 0.30)	3 [42-44]	92%	
Assesment tool					
Sensitivity analyses					
Correlation coefficient estimation [d]	567	-0.24 (-0.73 to 0.25)	3 [42-44]	70%	
Prosepctive study design	93	-0.11 (-1.74 to 1.52)	2 [43,44]	86%	
Funnel plot assymetry					-

Abbreviations: N, number; SMC, Standardized mean change; CI, Confidence interval; I^2; Heterogeneity; Chemo, Chemotherapy; TT, Targeted therapy; IO, Immunotherapy; Multiple, Studies combining multiple diagnoses; CT, Computed tomography; CM, Centimeter; SMI, Skeletal Muscle Index; SMA, Skeletal Muscle Area; PMI, Psoas (or Pectoralis) Muscle Index; PMA, Psoas (or Pectoralis) Muscle Area; BIA, Bioimpedance anaysis; Kg., Kilograms.

[a] Only subgroups with ≥2 studies are presented.

[b] Estimated means: Sensitivity analysis excluding studies where means and SDs were derived from medians/IQRs using Wan et al.'s method.

[c] Non-small studies: Sensitivity analysis excluding studies with n ≤ 100.

[d] Correlation coefficient estimation: Sensitivity analysis with imputed correlation coefficient (r = 0.5)

Table S12: Meta-analyses of the change in skeletal muscle mass during treatment of colorectal cancer

Colorectal cancer	*N*	SMC (95% CI)	Comparisons	I^2	*P*-value
Primary analysis	369	-0.15 (-0.37 to 0.07)	3[45-47]	77%	
Subgroup analyses [a]					
Treatment type					
Chemo-TT	200	-0.21 (-0.23 to -0.18)	2[46,47]	0%	
Treatment setting		-	-	-	
Assesment tool					
CT SMI (cm/m^2)	306	-0.13 (-1.07 to 0.81)	2[45,47]	86%	
Sensitivity analyses					
Estimated means [b]	369	-0.15 (-0.37 to 0.07)	3[45-47]	78%	
Non-small trials [c]	306	-0.13 (-1.07 to 0.81)	2[45,47]	87%	
Correlation coefficient estimation [d]	369	-0.16 (-0.37 to 0.04)	3[45-47]	33%	
Prosepctive study design	200	-0.21 (-0.23 to -0.18)	2[46,47]	0%	
By sex (male)	192	-0.08 (-0.37 to 0.20)	4 [46-49]	87%	
By sex (female)	140	-0.17 (-0.48 to 0.14)	4 [46-49]	85%	
Funnel plot assymetry					-

Abbreviations: N, number with complete data; SMC, Standardized mean change; CI, Confidence interval; I^2; Heterogeneity; Chemo, Chemotherapy; TT, Targeted therapy; IO, Immunotherapy; CT, Computed tomography; CM, Centimeter; SMI, Skeletal Muscle Index; SMA, Skeletal Muscle Area; PMI, Psoas (or Pectoralis) Muscle Index; PMA, Psoas (or Pectoralis) Muscle Area; BIA, Bioimpedance anaysis; Kg., Kilograms.

[a] Only subgroups with ≥2 studies are presented.

[b] Estimated means: Sensitivity analysis excluding studies where means and SDs were derived from medians/IQRs using Wan et al.'s method.

[c] Non-small studies: Sensitivity analysis excluding studies with n ≤ 100.

[d] Correlation coefficient estimation: Sensitivity analysis with imputed correlation coefficient (r = 0.5)

Table S13: Meta-analyses of the change in skeletal muscle mass during treatment of breast cancer

Breast cancer	*N*	SMC (95% CI)	Comparisons	I^2	*P*-value
Primary analysis	853	-0.14 (-0.28 to 0.00)	9 [50-58]	93%	
Subgroup analyses [a]					
Treatment type					
Chemotherapy	259	0.00 (-0.03 to 0.03)	3 [56-58]	0%	
Chemo-TT	483	-0.24 (-0.48 to 0.01)	5 [50-54]	93%	
Treatment setting					
Neoadjuvant	676	-0.22 (-0.47 to 0.02)	5 [50-53,58]	96%	
Adjuvant	66	0.01 (-0.04 to 0.05)	3 [54,56,57]	0%	
Assesment tool					
CT SMI (cm/m^2)	487	-0.02 (-0.07 to 0.02)	4 [53-55,58]	0%	
CT PMA (cm^2)	269	-0.40 (-0.66 to -0.15)	2 [50,51]	0%	
Sensitivity analyses					
Estimated means [b]	832	-0.16 (-0.31 to 0.01)	8 [50-53,55-58]	94%	
Non-small trials [c]	594	-0.13 (-0.43 to 0.18)	4 [50,53,55,58]	97%	
Correlation coefficient estimation [d]	855	-0.15 (-0.30 to -0.01)	9 [50-58]	72%	
Prosepctive study design	45	0.01 (-0.21 to 0.23)	2 [56,57]	0%	
Funnel plot assymetry					

Abbreviations: N, number with complete data; SMC, Standardized mean change; CI, Confidence interval; I^2; Heterogeneity; Chemo, Chemotherapy; TT, Targeted therapy; IO, Immunotherapy; CT, Computed tomography; CM, Centimeter; SMI, Skeletal Muscle Index; SMA, Skeletal Muscle Area; PMI, Psoas (or Pectoralis) Muscle Index; PMA, Psoas (or Pectoralis) Muscle Area; BIA, Bioimpedance anaysis; Kg., Kilograms.

[a] Only subgroups with ≥2 studies are presented.

[b] Estimated means: Sensitivity analysis excluding studies where means and SDs were derived from medians/IQRs using Wan et al.'s method.

[c] Non-small studies: Sensitivity analysis excluding studies with n ≤ 100.

[d] Correlation coefficient estimation: Sensitivity analysis with imputed correlation coefficient (r = 0.5).

Table S14: Prevalence of low skeletal muscle mass during systemic cancer treatment

Study and Location	Skeletal Muscle Index (SMI) cutoff value in cm^2/m^2 Males; Females	Body segment	Low skeletal muscle mass Baseline *N* (%)	Low skeletal muscle mass Follow-up *N* (%)	*P*-value
Pancreatic cancer					
Chemotherapy					
Jin (2022) [51]	< 41; < 38.5	L3	57 (48)	54 (45)	0.77
Uemura (2020) [23]	< 42; < 38	L3	33 (48)	51 (74)	NR
Urological cancers					
Chemotherapy					
Macdonald (2024) [57]	< 55; < 39	L3	38 (54)	41 (58)	0.73
Rimar (2018) [58]	< 55; < 38.5	L3	18 (69)	21 (81)	0.002
Lyon (2019) [59]	< 55; < 39	L3	101 (55)	107 (58)	NR
Lung cancer and pleural mesothelioma					
Chemotherapy					
Kidd (2024) [66]	(< 53 & BMI ≥ 25) or (< 43 & BMI < 25); < 41	L3	32 (35)	44 (48)	0.07
Chemotherapy + immunotherapy					
Chaunzwa (2024) [20]	(< 53 & BMI ≥ 25) or (< 43 & BMI < 25);	L3	NR (28)	NR (46)	NR

	< 41				
Chemotherapy + targeted therapy					
Cortellini (2018) [68]	< 53; < 41	L3	28 (35)	34 (59)	NR
Gastric, esophagastric and esophageal cancers					
Chemotherapy					
Juez (2024) [70]	(< 53 & BMI ≥ 25) or (< 43 & BMI < 25); SMI < 43	L3	NR (38)	NR (46)	0.125
Mirkin (2017) [71]	< 54.5; < 38.5	L3	7 (19)	12 (33)	NR
Boer (2020) [75]	< 52.4; < 38.5	L3	84 (42)	108 (54)	<0.001
Dijksterhuis (2019) [78]	(< 53 & BMI ≥ 25) or (< 43 & BMI < 25); < 41	L3	43 (49)	36 (55)	<0.001
Awad (2012) [28]	< 52.4; < 38.5	L3	27 (57)	37 (79)	0.046
Onishi (2024) [80] (DCF treatment)	< 42; < 38	L3	63 (91)	62 (90)	NR
Onishi (2024) [80] (CF treatment)	< 42; < 38	L3	130 (89)	140 (96)	NR
Yip (2014) [82]	< 52.4; < 38.5	L3	9 (26)	15 (43)	NR
Miyata (2017) [83]	< 90% of standard SMM according to Inbody	Whole body	44 (47)	50 (53)	0.382
Ishida (2019) [84]	< 6.36; < 3.92	L3	43 (26)	51 (31)	NR
Ovarian cancer					
Chemotherapy					

Wood (2023) [86]	NA; < 38	L4	79 (56)	79 (56)	NR
Yoshino (2020) [87]	NA; < 39	L3	36 (60)	41 (68)	0.025
Studies including cohorts with multiple cancers					
Chemotherapy + targeted therapy					
Oflazoglu (2020) [92]	< 10.76 kg/m² & handgrip strength (< 30 kg.);<br>< 6.76 kg/m² & handgrip strength (< 20 kg.)	Whole body	40 (15)	59 (21)	<0.001
Colorectal cancer					
Chemotherapy					
Okuno (2019) [96]	(< 53 & BMI ≥ 25) or (< 43 & BMI < 25);<br>< 41	L3	58 (34)	61 (36)	NR
Chemotherapy + targeted therapy					
Huemer (2019) [99]	< 52.4; < 38.5	L3	NR (41)	NR (59)	NR
Blauwhoff-Buskermolen (2016) [21]	NR	L3	NR (57)	NR (70)	NR
Gallois (2020) [30]	NR	L3	38 (26)	34 (25)	NR
Breast cancer					
Chemotherapy					
Jang (2022) [100] (AC-T)	< 38.5	L3	NR (25)	NR (26)	NR

Chemotherapy + immunotherapy (+ targeted therapy)					
Camilleri (2024) [103]	NA; < 39	L3	35 (36)	40 (42)	0.20
Karaca (2024) [106]	NA; (Right + Left PMA) ÷ 2 → average PMA; 25th percentile (415.4 mm^2) = cut-off.	L3	NR (25)	NR (41)	<0.01
Lee (2021) [107]	NR	T4	30 (12)	33 (13)	NR
Mazzuca (2018) [109]	NA; < 38.5	L3	8 (38)	10 (48)	NR
Lymphoma					
Chemotherapy + targeted therapy					
Xiao (2016) [110]	< 53; < 41	L3	NR (41)	NR (38)	NR
Prevalence of low muscle mass (mean %)			43%	51%	NA

Abbreviations: N: number; NR: not reported; NA: not applicable; Skeletal Muscle Index (SMI) calculated as cm^2/m^2; BMI (kg/m^2); Third Lumbar Vertebrae (L3)

Table S15: Excluded studies on full-text screening

Reason for full text exclusion	*N*
0. Duplet	179
1. Non-English Papers	7
2. Grey literature (e.g., dissertations, conference abstracts)	263
3. Not an adult cancer population	5
4. Not an observational study design (Not systematic reviews, not intervention study, i.e., exercise or nutrition)	12
5. No use of specific measurements for muscle mass (and report these outcomes, i.e., CT, MRI, DEXA, bioimpedance)	23
6. Not including at least two measurements of skeletal muscle mass pre- and post-chemotherapy or immunotherapy or a combination and report these outcomes.	266
7. Not exclusively chemo- or immunotherapy	108
**In total**	863

Author & Date	Title	Reason
Baldwin et al. 2015	Sarcopenia as a predictor of mortality in advanced pancreatic cancer	2
Beamish et al. 2014	Prognostic significance of CT muscle density in upper GI cancer	2
Neto et al. 2018	Association of sarcopenia with toxicities and survival after autologous hematopoietic stem cell transplantation for adults with lymphomas	2
Cortenellini et al. 2020	Weighing the role of skeletal muscle mass and muscle density in cancer patients receiving PD-1/PD-L1 checkpoint inhibitors: a multicenter real-life study	6
Cortellini et al. 2018	Single-institution study of correlations between skeletal muscle mass, its density, and clinical outcomes in non-small cell lung cancer patients treated with first-line chemotherapy	0
Gopaul et al. 2016	Survival and body composition analysis in hepatocellular carcinoma patients with myosteatosis	2
Hoeben et al. 2018	Muscle wasting is an independent prognostic factor in patients with locally advanced head and neck cancer treated with chemo- or bioradiation and predicts treatment toxicity but not tumour control	2
Kurniawan et al. 2019	Comparison Between Low and Normal Muscle Mass to Quality of Life in Breast Cancer Patients A Preliminary Study	2

Mishra et al 2020	CT-Defined Fat Index Is a Prognostic Factor of Chronic Graft-Versus-Host Disease Outcomes in Adult Allogeneic Transplant Recipients	2
Boiles et al. 2015	Establishing valid functional / PRO measures in cancer cachexia or sarcopenia to correlate with changes in Skeletal Muscle Mass Index (SMI): Results of a prospective study in patients with non-small cell lung cancer	2
Berghoff et al. 2016	Temporal muscle thickness (TMT) is an independent prognostic parameter in patients with newly diagnosed brain metastases (BM) of breast cancer (BC)	2
Salman et al 2017	Patterns of muscle loss in patients with pancreas cancer	2
Sanchez et al 2019	Surgical Resection after Neoadjuvant Chemoradiation in Patients with Sarcopenia and Pancreatic Cancer	2
Van der Werf et al 2017	Cancer Cachexia: Identification by Clinical Assessment versus International Consensus Criteria in Patients with Metastatic Colorectal Cancer	7
Vigano et al. 2009	Use of the scored Patient-Generated Subjective Global Assessment (PG-SGA) to characterize cachexia in newly diagnosed advanced cancer patients	2
Vigano et al 2010	Beyond the tip of the iceberg: The human cancer cachexia database	2
Abe et al 2017	Significance of sarcopenia as a prognostic factor for metastatic urothelial carcinoma patients treated with systemic chemotherapy	0
Abe et al 2018	Significance of sarcopenia as a prognostic factor for metastatic urothelial carcinoma patients treated with systemic chemotherapy	6
Aberle et al 2021	The Association between Body Composition and Overall Survival of Advanced Stage Pancreatic Cancer Patients Treated with FOLFIRINOX	2
Agatha et al 2018	Risk of sarcopenia as the side effect of chemotherapy among breast cancer patients: Preliminary study	2
Ahn et al 2015	Correlation of changes in lean muscle weight with outcome in metastatic pancreatic adenocarcinoma (mPDAC) who undergo taxane-based chemotherapy (CT)	2
Ahn et al 2016	Comprehensive analysis of radiographic, clinical, and inflammatory markers demonstrating changes in lean muscle correlate with outcome in patients (pts) with metastatic pancreatic adenocarcinoma (mPDAC) who undergo taxane-based chemotherapy (CT)	2
Ahn et al 2016	Comprehensive analysis of radiographic, clinical, and inflammatory markers demonstrating changes in lean muscle correlate with outcome in patients (pts) with metastatic pancreatic adenocarcinoma (mPDAC) who undergo taxane-based chemotherapy (CT)	0
Ahn et al 2016	Association of body composition with function in women with early breast cancer	6

Aleixo et al 2020	The association of body composition parameters and adverse events in women receiving chemotherapy for early breast cancer	7
Aleixo et al 2020	The association of body composition parameters and adverse events in women receiving chemotherapy for early breast cancer	6
Aleixo et al 2020	Myosteatosis evaluation using erector spinae and psoas muscles to predict adverse events during adjuvant chemotherapy for breast cancer	7
Aleixo et al 2021	Sorafenib Might Induce Sarcopenia in Patients With Hepatocellular Carcinoma by Inhibiting Carnitine Absorption	7
Amanuma et al 2020	The impact of sarcopenia on patients undergoing treatment for pancreatic ductal adenocarcinoma	4
Amundson et al 2020	Sarcopenic obesity: A probable risk factor for dose limiting toxicity during neo-adjuvant chemotherapy in oesophageal cancer patients	7
Anandavadivelan et al 2016	Sarcopenic obesity: A probable risk factor for dose limiting toxicity during neo-adjuvant chemotherapy in oesophageal cancer patients	7
Anandavadivelan et al 2016	Sarcopenia is associated with reduced survival in patients with advanced hepatocellular carcinoma undergoing sorafenib treatment	6
Antonelli et al 2018	Low body mass index and sarcopenia associated with dose-limiting toxicity of sorafenib in patients with renal cell carcinoma	6
Antoun et al 2010	No evidence for changes in skeletal muscle mass or weight during first-line chemotherapy for metastatic colorectal cancer	0
Antoun et al 2019	Skeletal muscle density predicts prognosis in patients with metastatic renal cell carcinoma treated with targeted therapies	6
Antoun et al 2013	The association between muscle mass and the degree of myosteatosis of the psoas muscle and mortality in older patients with cancer	0
Ariën et al 2021	Impact of Sarcopenia on Adverse Outcomes After Allogeneic Hematopoietic Cell Transplantation	6
Armenian et al 2019	Predictive Value of Skeletal Muscle Mass in Recurrent/Metastatic Head and Neck Squamous Cell Carcinoma Patients Treated With Immune Checkpoint Inhibitors	6
Arribas et al 2021	Changes in body weight, skeletal muscle and adipose tissue after gastrectomy: a comparison between proximal gastrectomy and total gastrectomy	0
Asaoka et al 2019	Factors which modulate the rates of skeletal muscle mass loss in non-small cell lung cancer patients: a pilot study	0

Atlan et al 2017	Prediction of Everolimus Toxicity and Prognostic Value of Skeletal Muscle Index in Patients With Metastatic Renal Cell Carcinoma	6
Auclin et al 2017	Impact of quantitative body composition on survival in patients with epithelial ovarian cancer undergoing primary debulking surgery	2
Ataseven et al 2018	Sarcopenia is a weak prognostic factor before chemoradiotherapy of esophageal carcinomas	2
Bethsabee et al 2019	A comparison of CT based measures of skeletal muscle mass and density from the Th4 and L3 levels in patients with advanced non-small-cell lung cancer	6
Gronberg et al 2018	Explaining the obesity paradox: The association between body composition and colorectal cancer survival (c-scans study)	6
Caan et al 2017	Clinical impact of postgastrectomy sarcopenia on the prognosis in patients with gastric cancer	2
Kim et al 2020	Clinical impact of newly developed sarcopenia after surgical resection for gastric cancer	2
Kim et al 2020	Impact of body morphology on survival in patients with bone metastases: A prospective cohort study	2
Pielkenrood et al 2019	Impact of sarcopenia (Sp) on outcomes in patients (pts) undergoing trimodality therapy for esophageal cancer	2
Weston et al 2015	Postdiagnosis Loss of Skeletal Muscle, but Not Adipose Tissue, Is Associated with Shorter Survival of Patients with Advanced Pancreatic Cancer	0
Babic et al 2019	Baseline Sarcopenia is Associated with Lack of Response to Therapy, Liver Decompensation and High Mortality in Hepatocellular Carcinoma Patients	0
Badran et al 2020	Sarcopenia prior to and following chemotherapy to predict morbidity in patients undergoing post-chemotherapy retroperitoneal lymphadenectomy (PC-RPLND)	2
Baky et al 2021	Impact of body composition, nutritional and inflammatory status on outcome of non-small cell lung cancer patients treated with immunotherapy	4
Baldessari et al 2021	Impact of primary breast cancer therapy on energetic capacity and body composition	7
Ballinger 2018	BODY COMPOSITION IMPACT ON SURVIVAL AND TOXICITY OF TREATMENT IN PANCREATIC CANCER: CROSS-SECTIONAL PILOT STUDY	6
Barrére et al 2020	Sarcopenia is linked to treatment toxicity in patients with metastatic colorectal cancer	6
Barret et al 2014	The IMPACT study: early loss of skeletal muscle mass in advanced pancreatic cancer patients	0

Basile et al 2019	Sarcopenia predicts reduced survival in patients with hepatocellular carcinoma at first diagnosis	6
Begini et al 2017	Muscle Loss Is Associated with Overall Survival in Patients with Metastatic Colorectal Cancer Independent of Tumor Mutational Status and Weight Loss	6
Best et al 2021	Cancer-associated malnutrition: Prevalence of cachexia, sarcopenia and impact on health-related quality of life and survival in a cohort of Irish ambulatory patients receiving chemotherapy.	2
Bhuachalla et al 2016	Prognostic values of abdominal body compositions on survival in advanced pancreatic cancer	6
Bian et al. 2016	Combined Effect of Sarcopenia and Systemic Inflammation on Survival in Patients with Advanced Stage Cancer Treated with Immunotherapy	0
Bilen et al 2020	Sarcopenia and inflammation predicts survival in advanced stage cancer patients (pts) treated with immunotherapy (IO)	2
Bilen et al 2018	Combined Effect of Sarcopenia and Systemic Inflammation on Survival in Patients with Advanced Stage Cancer Treated with Immunotherapy	6
Bilen et al 2020	Sarcopenia is associated with worse overall survival in patients with anal squamous cell cancer	6
Bingmer et al. 2020	Prognostic Value of Computed Tomography: Measured Parameters of Body Composition in Primary Operable Gastrointestinal Cancers	6
Black et al 2017	Loss of muscle mass during chemotherapy is predictive for poor survival of patients with metastatic colorectal cancer	0
Blauwhoff-Buskermolen et al. 2016	The influence of different muscle mass measurements on the diagnosis of cancer cachexia	6
Blauwhoff-Buskermolen et al. 2017	Prediction of 90 Day and Overall Survival after Chemoradiotherapy for Lung Cancer: Role of Performance Status and Body Composition	0
Bowden et al 2017	Cachexia-related biomarkers predict shortened survival and treatment-related adverse outcomes in a population receiving palliative chemotherapy for lung cancer	2
Bowden et al. 2019	Sarcopenia in Advanced Serous Ovarian Cancer	0
Bronger et al 2017	Weight stability masks changes in body composition in colorectal cancer: a retrospective cohort study	0
Brown et al. 2021	The deterioration of muscle mass and radiodensity is prognostic of poor survival in stage I-III colorectal cancer: a population-based cohort study (C-SCANS)	0

Brown et al. 2018	Evaluation of the impact of cachexia on clinical outcomes in aggressive lymphoma	6
Burkart et al. 2019	Weight loss of 5% or more predicts loss of fat-free mass during palliative chemotherapy in patients with advanced cancer: A pilot study	0
Buskermolen et al. 2012	Sarcopenia is a predictive factor on morbidity and overall survival in patients with colorectal cancer peritoneal metastasis	2
Agalar et al. 2018	Post-radiotherapy sarcopenia: a new prognostic factor in oropharyngeal cancers?	2
Dupin et al. 2019	Sarcopenia as an independent prognostic factor for survival and perioperative complications in patients with gastric cancer	6
Koch et al. 2018	Muscle anabolism in advanced cancer: Is cachexia an immutable phenomenon?	2
Prado et al. 2011	Central tenet of cancer cachexia therapy: Do patients with advanced cancer have exploitable anabolic potential?	0
Prado et al. 2013	Impact of sarcopenia on adverse effects in trimodality therapy for esophageal carcinoma	2
Pranje et al. 2016	Increased visceral to subcutaneous fat ratio is associated with decreased overall survival in pancreatic cancer	2
Nwachukwu et al. 2016	Muscle MASS and mortality in cancer patients	2
Salas et al. 2019	Assessment of sarcopenia and changes in body composition after neoadjuvant chemotherapy and associations with clinical outcomes in oesophageal cancer	0
Yip et al. 2013	Association of Muscle and Adiposity Measured by Computed Tomography With Survival in Patients With Nonmetastatic Breast Cancer	6
Caan et al. 2018	Resting energy expenditure and body mass changes in women during adjuvant chemotherapy for breast cancer	0
Campbell et al. 2007	Prognostic impact of fat tissue loss and cachexia assessed by computed tomography scan in elderly patients with diffuse large B-cell lymphoma treated with immunochemotherapy	6
Camus et al. 2014	Prognostic impact of body composition (BC) changes during neoadjuvant chemotherapy (NACT) in breast cancer patients (pts)	2
Carbogning et al. 2020	Impact on prognosis of early weight loss during palliative chemotherapy in patients diagnosed with advanced pancreatic cancer	5
Carnie et al 2020	Role of baseline computed-tomography-evaluated body composition in predicting outcome and toxicity from first-line therapy in advanced gastric cancer patients	6
Catanese et al 2021	Impact of genetic mutations and nutritional status on the survival of patients with colorectal cancer	6

Cavagnari et al. 2019	Body Composition, Adherence to Anthracycline and Taxane-Based Chemotherapy, and Survival After Nonmetastatic Breast Cancer	7
Cespedes Feliciano et al. 2020	Muscle mass at the time of diagnosis of nonmetastatic colon cancer and early discontinuation of chemotherapy, delays, and dose reductions on adjuvant FOLFOX: The C-SCANS study	7
Cespedes Feliciano et al. 2017	Sarcopenia, as assessed on computed tomography, as a predictive factor for curative colon cancer resection outcomes: A 5-year Australian cohort	2
Chai et al 2020	Quantitative body mass characterization before and after head and neck cancer radiotherapy: A challenge of height-weight formulae using computed tomography measurement	0
Chamchod et al. 2016	Prognostic value of adipose tissue and muscle mass in advanced colorectal cancer: A post hoc analysis of two non-randomized phase II trials	0
Charette et al. 2019	The association of cisplatin pharmacokinetics and skeletal muscle mass in patients with head and neck cancer: The prospective PLATISMA study	7
Chargi et al. 2022	The Role of Baseline Sarcopenia Index in Predicting Chemotherapy-Induced Undesirable Effects and Mortality in Older People with Stage III or IV Non-Small Cell Lung Cancer	7
Chen et al 2021	Skeletal muscle depletion predicts survival of patients with advanced biliary tract cancer undergoing palliative chemotherapy	0
Cho et al. 2017	Impact of sarcopenia on survival of pancreatic cancer patients treated with concurrent chemoradiotherapy	6
Cho et al. 2020	Prognostic Significance of Sarcopenia With Inflammation in Patients With Head and Neck Cancer Who Underwent Definitive Chemoradiotherapy	6
Cho et al. 2018	Preoperative sarcopenia and post-operative accelerated muscle loss negatively impact survival after resection of pancreatic cancer	0
Choi et al. 2018	Prognostic value of computed tomography-based volumetric body composition analysis in patients with head and neck cancer: Feasibility study	0
Choi et al 2020	Change of skeletal muscle index during the chemotherapy as a prognostic factor of survival in pancreatic cancer patients receiving palliative chemotherapy.	2
Choi et al. 2015	Skeletal Muscle Depletion Predicts the Prognosis of Patients with Advanced Pancreatic Cancer Undergoing Palliative Chemotherapy, Independent of Body Mass Index	0
Choi et al. 2015	Body composition is prognostic and predictive of ipilimumab activity in metastatic melanoma	6

Chu et al. 2020	Body composition is prognostic and predictive of ipilimumab activity in metastatic melanoma	0
Chu et al. 2020	Prognostic significance of sarcopenia and skeletal muscle mass change during preoperative chemoradiotherapy in locally advanced rectal cancer	4
Chung et al. 2020	Changes in Body Composition During Adjuvant FOLFOX Chemotherapy and Overall Survival in Non-Metastatic Colon Cancer	0
Chung et al. 2019	Anthropometric Changes in Patients with Pancreatic Cancer Undergoing Preoperative Therapy and Pancreatoduodenectomy	0
Cloyd et al 2018	The value of physical performance measurements alongside assessment of sarcopenia in predicting receipt and completion of planned treatment in non-small cell lung cancer: an observational exploratory study	7
Collins et al. 2018	The value of physical performance measurements alongside assessment of sarcopenia in predicting receipt and completion of planned treatment in non-small cell lung cancer: an observational exploratory study	6
Collins et al 2018	Patients with sarcopenia benefit from neoadjuvant chemotherapy in advanced ovarian cancer	2
Conrad et al. 2017	Characterization of Anthropometric Changes that Occur During Neoadjuvant Therapy for Potentially Resectable Pancreatic Cancer	0
Cooper et al. 2015	Characterization of Anthropometric Changes that Occur During Neoadjuvant Therapy for Potentially Resectable Pancreatic Cancer	0
Cooper et al. 2015	Weighing the role of skeletal muscle mass and muscle density in cancer patients receiving PD-1/PD-L1 checkpoint inhibitors: a multicenter real-life study	6
Cortellini et al. 2020	Weighing the role of skeletal muscle mass and muscle density in cancer patients receiving PD-1/PD-L1 checkpoint inhibitors: a multicenter real-life study	6
Cortellini et al. 2020	Impact of CT-based body composition parameters at baseline, their early changes and response in metastatic cancer patients treated with immune checkpoint inhibitors	0
Crombe et al. 2020	Body composition by computed tomography as a predictor of toxicity to docetaxel chemotherapy in patients with metastatic prostate cancer	2
Cushen et al. 2014	A prospective investigation of nutritional status of ambulatory Irish oncology patients undergoing chemotherapy: Prevalence of malnutrition, cachexia, sarcopenia and impact on quality of life	2

Cushen et al 2013	Body Composition by Computed Tomography as a Predictor of Toxicity in Patients With Renal Cell Carcinoma Treated With Sunitinib	6
Cushen et al. 2017	Early loss of skeletal muscle mass (LSMM) as prognostic factor in metastatic pancreatic cancer (PC) patients.	2
Basile et al. 2017	Computed tomography (CT)-Defined sarcopenia is prevalent in patients with neuroendocrine neoplasms (NENs)	2
Chan et al. 2019	Prognostic significant of loss of muscle mass in perioperative period	2
Furukawa et al. 2017	Determinants of quality of life and survival in ambulatory oncology patients receiving chemotherapy	2
Power et al. 2018	Impact of body composition parameters on clinical outcomes in patients with metastatic castration-resistant prostate cancer treated with docetaxel	2
Power et al. 2015	A prospective investigation of nutritional status in 517 Irish cancer patients undergoing chemotherapy: Prevalence of malnutrition, cachexia, sarcopenia and impact on quality of life	2
Power et al. 2015	Sarcopenia and altered body composition following abiraterone acetate (AA) and corticosteroid (C) treatment in men with castration-refractory prostate cancer (CRPC)	2
Mukherji et al. 2012	Impact of sarcopenia and phase angle on survival of palliative breast cancer patients	2
Perez et al. 2018	Assessment of skeletal muscle mass as a predictive factor for chemotherapy toxicity and TTP in advanced nsclc patients with cancer cachexia	2
Srdic et al. 2016	CLINICAL RELATIONSHIP BETWEEN THE CHANGES IN MUSCLE MASS AND PROGNOSIS AFTER CONCURRENT CHEMO-RADIATION THERAPY IN PATIENTS WITH ESOPHAGEAL CANCER	2
Dw. Ma et al. 2018	Cachexia index utilization in patients with non-small cell lung cancer	2
Dw. Rybar et al. 2016	Is cachexia associated with chemotherapy toxicities in gastrointestinal cancer patients? A prospective study	6
daRocha et al. 2019	Relationships among body mass index, longitudinal body composition alterations, and survival in patients with locally advanced pancreatic cancer receiving chemoradiation: a pilot study	4
Dalal et al. 2012	Loss of skeletal muscle during systemic chemotherapy is prognostic of poor survival in patients with foregut cancer	4
Daly et al. 2018	The impact of body composition parameters on ipilimumab toxicity in metastatic melanoma and longitudinal changes in body composition during treatment	2

Daly et al. 2015	The impact of body composition parameters on ipilimumab toxicity in metastatic melanoma and longitudinal changes in body composition during treatment	0
Daly et al. 2015	The impact of body composition parameters on ipilimumab toxicity and survival in patients with metastatic melanoma	0
Daly et al. 2017	Body composition predictors of therapy response in patients with primary extremity soft tissue sarcomas	6
De Amorim Bernstein et al. 2018	72P The association between skeletal muscle measures and chemotherapy-induced toxicity in non-small cell lung cancer patients treated with first-line platinum-based chemotherapy: A prospective follow-up study	2
De Jong et al. 2021	Skeletal muscle loss during neoadjuvant therapy negatively impacts on prognosis in patients with locally advanced low rectal cancer	2
De nardi et al. 2016	Changes in body composition during neoadjuvant therapy can affect prognosis in rectal cancer patients: An exploratory study	7
De nardi et al. 2020	Evolution of Body Composition Following Autologous and Allogeneic Hematopoietic Cell Transplantation: Incidence of Sarcopenia and Association with Clinical Outcomes	0
Defillipp et al 2018	The prognostic value of weight and body composition changes in patients with non-small-cell lung cancer treated with nivolumab	6
Degens et al. 2021	The prognostic value of early onset, CT derived loss of muscle and adipose tissue during chemotherapy in metastatic non-small cell lung cancer	0
Degens et al. 2019	Changes in body composition during first cycle of chemotherapy in metastatic non-small cell lung cancer (NSCLC) are predictive for poor overall survival	0
Degens et al. 2017	The relationship between body composition and response to neoadjuvant chemotherapy in women with operable breast cancer	5
Del fabbro et al. 2012	Impact on postoperative complications of changes in skeletal muscle mass during neoadjuvant chemotherapy for gastro-oesophageal cancer	0
denBoer et al. 2020	Progressive Sarcopenia in Patients With Colorectal Cancer Predicts Survival	0
Deng et al. 2018	Prognostic value of skeletal muscle index and monocyte-to-lymphocyte ratio for lymph node-positive breast cancer patients after mastectomy	6
Deng et al. 2019	Factors Contributing to Cancer-Related Muscle Wasting During First-Line Systemic Treatment for Metastatic Colorectal Cancer	0
Derksen et al. 2019	The association between changes in muscle mass and quality of life in patients with metastatic colorectal cancer	2

Derksen et al. 2020	Accelerated muscle and adipose tissue loss may predict survival in pancreatic cancer patients: the relationship with diabetes and anaemia	0
Di Sebastiano et al. 2013	Prognostic value of low psoas muscle mass in patients with cervical spine metastasis	6
Dohzono et al. 2019	Low paravertebral muscle mass in patients with bone metastases from lung cancer is associated with poor prognosis	6
Dohzono et al. 2020	Concurrent losses of skeletal muscle mass, adipose tissue and bone mineral density during bevacizumab / cytotoxic chemotherapy treatment for metastatic colorectal cancer	0
Dolly et al. 2020	Concurrent losses of skeletal muscle mass, adipose tissue and bone mineral density during bevacizumab / cytotoxic chemotherapy treatment for metastatic colorectal cancer	7
Dolly et al. 2020	Sarcopenia and pretreatment anemia as prognostic factors for patients with localized muscle invasive bladder cancer treated by neoadjuvant chemotherapy and radical cystectomy	2
Billion et al 2019	Changes in body composition during adjuvant folfox chemotherapy and overall survival in non- metastatic colon cancer	0
Chung et al. 2020	Weight history and nutritional status in advanced cancer patients	2
De Rosa et al. (??)	Prognostic Importance of Sarcopeni and Inflammatory Statements in Stage III Non Small Cell Lung Carcinoma	2
Gumustepe et al. 2018	Impact of muscle mass, nutrition alstatus and muscle strength on out comes following surgery for esophageal cancer	2
Hagens et al. 2018	Effects of abiraterone acetate and enzalutamide on muscle and adipose mass in men with metastatic castration-resistant prostate cancer (mCRPC)	2
Ileana et al. 2013	Association of Systemic Inflammation and Sarcopenia With Survival in Nonmetastatic Colorectal Cancer: Results From the C SCANS Study	6
Feliciano et al. 2017	Malnutrition and body composition predict poor quality of life and reduced survival in ambulatory oncology patients receiving chemotherapy: A cross sectional study of 1015 patients	2
Sullivan et al. 2017	Loss of subcutaneous adipose tissue during chemotherapy predicts reduced survival in patients with incurable colorectal cancer undergoing palliative therapy	2
Sullivan et al. 2019	Predictors of survival in patients with incurable cancer	2
Sullivan et al. 2019	Prevalence of and factors associated with sarcopenia in Korean cancer survivors: Based on data obtained by korea national health and nutrition examination survey (knhanes) 2008-2011	6

E.y Kim et al. 2016	Prognostic Significance of CT-Determined Sarcopenia in Patients with Small-Cell Lung Cancer	6
E.y Kim et al. 2015	Skeletal muscle density as a positive predictive factor for nivolumab therapy in patients with metastatic renal cell carcinoma	2
Ekenel et al. 2020	Sarcopenia: Prevalence, and Impact on Operative and Oncologic Outcomes in the Multimodal Management of Locally Advanced Esophageal Cancer	0
Elliott et al. 2017	Impact of sarcopenia on prediction of progression-free survival and overall survival of patients with pancreatic ductal adenocarcinoma receiving first-line gemcitabine and nab-paclitaxel chemotherapy	6
Emori et al. 2022	The impact of neoadjuvant chemotherapy on skeletal muscle depletion and preoperative sarcopenia in patients with resectable colorectal liver metastases	0
Eriksson et al. 2017	The impact of neoadjuvant chemotherapy on skeletal muscle depletion in patients with resectable colorectal liver metastases	0
Eriksson et al. 2016	THE ASSOCIATION BETWEEN MUSCLE MASS AND THE DEGREE OF MYOSTEATOSIS OF THE PSOAS MUSCLE AND MORTALITY IN OLDER PATIENTS WITH CANCER	2
Arien et al. 2019	Patients triaged to neoadjuvant chemotherapy have higher rates of sarcopenia: An opportunity for prehabilitation	0
Fadadu et al. 2021	Body Composition, Adherence to Anthracycline and Taxane-Based Chemotherapy, and Survival after Nonmetastatic Breast Cancer	0
Feliciano et al. 2020	Sarcopenia and myosteatosis in patients undergoing curative radiotherapy for head and neck cancer: Impact on survival, treatment completion, hospital admission and cost	7
Findlay et al. 2020	Influence of treatment with abiraterone and enzalutamide on development of sarcopenia in patients with metastatic castration resistant prostate cancer	0
Fischer et al. 2020	Computed tomography-based analyses of baseline body composition parameters and changes in breast cancer patients under treatment with CDK 4/6 inhibitors	7
Franzoi et al. 2020	Computed tomography-based analyses of baseline body composition parameters and changes in breast cancer patients under treatment with CDK 4/6 inhibitors	0
Franzoi et al. 2020	Body Composition Adjusted Dosing of Gemcitabine-Nab-Paclitaxel in Pancreatic Cancer Does Not Predict Toxicity Compared to Body Surface Area Dosing	6
Freckelton et al. 2019	Is skeletal muscle a predictor of toxicity in pancreatic cancer patients on gemcitabine-based chemotherapy regimens?	2

Freckelton et al. 2017	Cancer cachexia reduces the efficacy of nivolumab treatment in patients with advanced gastric cancer	5
Fujii et al. 2020	Postoperative pneumonia causes the loss of skeletal muscle volume and poor prognosis in patients undergoing esophagectomy for esophageal cancer	0
Fujishima et al. 2021	Sarcopenia, intramuscular fat deposition, and visceral adiposity independently predict the outcomes of hepatocellular carcinoma	6
Fujiwara et al. 2015	A retrospective cohort study to investigate the incidence of cancer-related weight loss during chemotherapy in gastric cancer patients	5
Fukahori et al. 2021	Change in PMI during neoadjuvant therapy is a predictive prognostic marker in rectal cancer	7
Fukuoka et al. 2019	Impact of sarcopenia on the efficacy of pembrolizumab in patients with advanced urothelial carcinoma: a preliminary report	6
Fukushima et al. 2020	Posttherapeutic skeletal muscle mass recovery predicts favorable prognosis in patients with advanced urothelial carcinoma receiving first-line platinum-based chemotherapy	0
Fukushima et al. 2018	Postoperative Changes in Skeletal Muscle Mass Predict Survival of Patients With Metastatic Renal Cell Carcinoma Undergoing Cytoreductive Nephrectomy	0
Fukushima et al. 2017	Factors associated with muscle function in patients with hematologic malignancies undergoing chemotherapy	6
Fukushima et al. 2020	Sarcopenia is associated with reduced survival in patients with advanced hepatocellular carcinoma undergoing sorafenib treatment	6
Antonelli et al. 2018	Body composition profile impacts short and long term outcomes in patients following colorectal cancer surgery	2
Malietzis et al. 2015	The impact of sarcopenia on toxicity and pharmacokinetics of 5-fluorouracil (5FU) in colorectal cancer	2
Williams et al. 2017	Accelerated sarcopenia and outcomes in older adults with cancer: The Health ABC Study	2
Williams et al. 2019	Skeletal mass depletion is a negative prognostic factor in gastrointestinal cancer patients in the terminal stage. [Japanese]	1
Takahashi et al. 2015	POSTOPERATIVE SKELETAL MUSCLE LOSS NEGATIVELY IMPACT SURVIVAL OF COLORECTAL CANCER	2
Wu et al. 2019	Impact of sarcopenia on outcomes of locally advanced esophageal cancer patients treated with neoadjuvant chemoradiation followed by surgery	2
Murimwa et al. 2016	Sarcopenia as a prognostic factor in Hepatolithiasis-associated intrahepatic cholangiocarcinoma patients following hepatectomy: A retrospective study	6

Zhou et al. 2015	Myosteatosis in a systemic inflammation-dependent manner predicts favorable survival outcomes in locally advanced esophageal cancer	6
Gabiatti et al. 2019	Skeletal muscle loss during chemotherapy and its association with survival and systemic treatment toxicity in metastatic colorectal cancer: An AGEO prospective multicenter study	0
Gallois et al. 2021	The impact of sarcopenia on tolerance of radiation and outcome in patients with head and neck cancer receiving chemoradiation	6
Ganju et al.2019	The impact of skeletal muscle abnormalities on tolerance to adjuvant chemotherapy and radiation and outcome in patients with endometrial cancer	6
Ganju et al. 2020	Disentangling the body weight-bone mineral density association among breast cancer survivors: an examination of the independent roles of lean mass and fat mass	6
George et al. 2013	Sarcopenia and inflammation are independent predictors of survival in male patients newly diagnosed with small cell lung cancer	6
Go et al. 2016	Prognostic impact of sarcopenia in patients with diffuse large B-cell lymphoma treated with rituximab plus cyclophosphamide, doxorubicin, vincristine, and prednisone	6
Go et al. 2016	Characterising the impact of body composition change during neoadjuvant chemotherapy for pancreatic cancer	0
Griffin et al 2019	Skeletal muscle depletion is associated with disease progression during neo-adjuvant therapy for borderline resectable pancreatic adenocarcinoma	2
Griffin et al. 2017	Body composition change during chemotherapy for borderline resectable pancreatic cancer	2
Griffin et al. 2019	Associations between severe co-morbidity and muscle measures in advanced non-small cell lung cancer patients	7
Grønberg et al. 2019	A comparison of CT based measures of skeletal muscle mass and density from the Th4 and L3 levels in patients with advanced non-small-cell lung cancer	6
Grønberg et al. 2019	Association of Body Composition With Survival and Locoregional Control of Radiotherapy-Treated Head and Neck Squamous Cell Carcinoma	7
Grossberg et al. 2016	Sarcopenia during neoadjuvant therapy for oesophageal cancer: characterising the impact on muscle strength and physical performance	7
Guinan et al. 2018	Body Composition as a Predictor of Toxicity and Prognosis in Patients with Diffuse Large B-Cell Lymphoma Receiving R-CHOP Immunochemotherapy	7
Guo et al. 2021	Sarcopenia as a prognostic biomarker of metastatic renal cell carcinoma: Its effect on survival benefit from cytoreductive nephrectomy	2

Fukushima et al. 2015	Prognostic significance of sarcopenia in metastatic renal cell carcinoma: Its association with survival benefit from cytoreductive nephrectomy	2
Fukushima et al. 2015	Sarcopenia is a prognostic biomarker of advanced urothelial carcinoma	2
Fukushima et al. 2014	Tetra-modality bladder sparing therapy can be a viable treatment option for muscle-invasive bladder cancer patients with sarcopenia	2
Fukushima et al. 2019	Post-therapeutic recovery of skeletal muscle mass predicts favorable prognosis in advanced urothelial carcinoma patients receiving 1st-line platinum-based chemotherapy	2
Fukushima et al. 2017	Prognostic significance of sarcopenia in upper tract urothelial carcinoma patients who underwent radical nephroureterectomy	2
Fukushima et al. 2017	Prognostic significance of sarcopenia in upper tract urothelial carcinoma patients who underwent radical nephroureterectomy	0
Fukushima et al. 2016	Postoperative recovery of skeletal muscle mass is associated with favorable prognosis in metastatic renal cell carcinoma patients who underwent cytoreductive nephrectomy	2
Fukushima et al. 2016	Impact of cancer cachexia on survival during chemotherapy in patients with upper gastrointestinal cancer	2
Goto 2017	Prognostic impact of quantitative imaging analysis of lean body mass after chemoradiation therapy for patients with advanced nasopharyngeal cancer	2
Inokuchi et al. 2018	Effect of changes in skeletal muscle mass on oncological outcomes during first-line sunitinib therapy for metastatic renal cell carcinoma	0
Ishihara et al. 2019	Association of sarcopenia with metabolic syndrome in Korean cancer survivors	1
Kim et al. 2017	Clinical relevance of cachexia assessed by an anthropometric tool in elderly patients with diffuse large b-cell lymphoma treated by immunochemotherapy	2
Lanic et al. 2013	Sarcopenia determined by computed tomography imaging is an independant prognostic factor in elderly patients with diffuse large B CELL lymphoma treated by immunochemotherapy	2
Lanic et al. 2012	Sarcopenia is associated with postoperative complication after pancreaticoduodenectomy	2
Lee et al. 2019	Staging of nutrition disorders in 531 non-small cell lung cancer (NSCLC) patients: Benefit from skeletal muscle mass, anorexia and performance status assessments	2
Morel et al. 2017	Significance of muscle mass decreasing on prognosis after pancreatic cancer surgery	2
Mori et al. 2018	Clinical impact of sarcopenia and skeletal muscle mass change during chemotherapy on outcomes of diffuse large b-cell lymphoma	2

Teranaka et al. 2017	Muscle wasting associated with poor outcome in patients with hepatocellular carcinoma undergoing sorafenib treatment	2
Ueki et al. 2016	Effect of muscle mass on toxicity and survival in patients with colon cancer undergoing adjuvant chemotherapy	6
H-W Jung et al. 2014	Morphometrics predicts overall survival in patients with lung, breast, prostate, or myeloma spine metastases, regardless of histology	2
Zakaria et al. 2018	Decrease in skeletal muscle index one year after radical cystectomy as a prognostic indicator in patients with urothelial bladder cancer	0
Yun-Sok et al. 2019	Influence of body composition and muscle strength on outcomes after multimodal oesophageal cancer treatment	0
Hagens et al. 2020	The role of sarcopenia in patients with intrahepatic cholangiocarcinoma: Prognostic marker or hyped parameter?	6
Hahn et al. 2019	Associations between muscle measures, survival, and toxicity in patients with limited stage small cell lung cancer	6
Halvorsen et al. 2020	Prognostic and clinical impact of sarcopenia in esophageal squamous cell carcinoma	6
Harada et al. 2016	O16-2 Prognostic impacts of change in skeletal muscle mass during neoadjuvant chemotherapy in patients with esophageal cancer	2
Harada et al. 2021	Low skeletal muscle density is associated with poor survival in patients who receive chemotherapy for metastatic gastric cancer	6
Hayashi et al. 2016	Skeletal muscle loss during neoadjuvant chemotherapy is a predictor of major postoperative complications in patients with esophagus cancer	2
Higashizono et al. 2021	Nutritional status, cachexia, and anorexia in women with peritoneal metastasis and intraperitoneal chemotherapy: A longitudinal analysis	0
Hilal et al. 2017	Muscle volume loss as a prognostic marker in hepatocellular carcinoma patients treated with sorafenib	0
Hiraoka et al. 2017	Impact of muscle volume and muscle function decline in patients undergoing surgical resection for hepatocellular carcinoma	6
Hiraoka et al. 2018	Effect of physical activity on bone strength and body composition in breast cancer premenopausal women during endocrine therapy	4
Hojan et al. 2013	Evaluation of sarcopenia, sarcopenic obesity, and phase angle in geriatric gastrointestinal cancer patients: before and after chemotherapy	7

Hopanci et al. 2019	Change in Skeletal Muscle Following Resection of Stage I-III Colorectal Cancer is Predictive of Poor Survival: A Cohort Study	0
Hopkins et al. 2019	The Impact of Muscle and Adipose Tissue on Long-term Survival in Patients With Stage I to III Colorectal Cancer	6
Hopkins et al. 2019	Prognostic significance of the skeletal muscle index and an inflammation biomarker in patients with breast cancer who underwent postoperative adjuvant radiotherapy	6
Hua et al. 2019	Low Skeletal Muscle Mass Impairs Quality of Life in Nasopharyngeal Carcinoma Patients Treated With Concurrent Chemoradiotherapy	6
Hua et al. 2020	Association between sarcopenia and clinical outcomes in patients with esophageal cancer under neoadjuvant therapy	7
Huang et al. 2020	Muscle loss during primary debulking surgery and chemotherapy predicts poor survival in advanced-stage ovarian cancer	0
Huang et al. 2020	Association Between Sarcopenia and Clinical Outcomes in Patients With Esophageal Cancer Under Neoadjuvant Therapy	6
Huang et al. 2020	Value of Sarcopenia defined by the new EWGSOP2 consensus for the prediction of Postoperative Complications and Long-term Survival after Radical Gastrectomy for Gastric Cancer: A comparison with four common nutritional screening tools	6
Huang et al. 2020	Severe muscle loss during radical chemoradiotherapy for non-metastatic nasopharyngeal carcinoma predicts poor survival	7
Huang et al. 2019	Body Composition in Patients with Radioactive Iodine-Refractory, Advanced Differentiated Thyroid Cancer Treated with Sorafenib or Placebo: A Retrospective Analysis of the Phase III DECISION Trial	0
Huillard et al. 2019	Changes of Skeletal Muscle Mass during Palliative Chemotherapy in Older Patients with Advanced Gastric Cancer	0
Hwang et al.	Loss of skeletal muscle mass during palliative chemotherapy is a poor prognostic factor in patients with advanced gastric cancer	2
Hwang et al. 2019	The prognostic impact of sarcopenic change after 8 weeks of 1st line gemcitabine based chemotherapy in advanced pancreatic adenocarcinoma	2
Kim et al. 2018	Psoas muscle measurements are inferior to total skeletal muscle measurements in the assessment of sarcopenia in ovarian cancer	2
Rutten et al. 2017	Prognostic factors for risk stratification of patients with recurrent or metastatic pancreatic adenocarcinoma who were treated with gemcitabine-based chemotherapy	6

Park et al. 2016	Changes in skeletal muscle mass during neoadjuvant chemotherapy are related to survival in ovarian cancer	2
Rutten et al. 2015	Impact of nutritional derangement on treatment outcome in advanced non-small-cell lung cancer (A-NSCLC) patients (pts)	2
Trestini et al. 2019	P1.16-43 Prevalence of Clinical and Sub-Clinical Malnutrition in Advanced Non-Small-Cell Lung Cancer Patients and Association with Outcome	2
Trestini et al. 2019	Muscle mass in elderly patients with DLBCL treated with RCHOP	2
Vaxman et al. 2016	Prognostic impact of sarcopenia in patients with metastatic hormone-sensitive prostate cancer	6
Ikeda et al. 2020	Prognostic impact of the psoas muscle index, a parameter of sarcopenia, in patients with diffuse large B-cell lymphoma treated with rituximab-based chemoimmunotherapy	7
Iltar et al. 2021	Rapid Depletion of Subcutaneous Adipose Tissue during Sorafenib Treatment Predicts Poor Survival in Patients with Hepatocellular Carcinoma	0
Imai et al. 2020	Skeletal muscle depletion predicts the prognosis of patients with hepatocellular carcinoma treated with sorafenib	6
Imai et al. 2015	Rapid Depletions of Subcutaneous Fat Mass and Skeletal Muscle Mass Predict Worse Survival in Patients with Hepatocellular Carcinoma Treated with Sorafenib	7
Imai et al. 2019	Skeletal muscle depletion is an independent prognostic factor for hepatocellular carcinoma	6
Iritani et al. 2015	Is cardiac wasting accompanied by skeletal muscle loss in breast cancer patients receiving anticancer treatment?	2
Ishida et al. 2017	Quantity and Quality of Skeletal Muscle as an Important Predictor of Clinical Outcomes in Patients with Esophageal Cancer Undergoing Esophagectomy after Neoadjuvant Chemotherapy	6
Ishida et al. 2021	Sarcopenia and the Modified Glasgow Prognostic Score are Significant Predictors of Survival Among Patients with Metastatic Renal Cell Carcinoma Who are Receiving First-Line Sunitinib Treatment	6
Ishida et al. 2016	Effect of Changes in Skeletal Muscle Mass on Oncological Outcomes During First-Line Sunitinib Therapy for Metastatic Renal Cell Carcinoma	0
Ishida et al. 2018	Effect of pretreatment psoas muscle mass on survival for patients with unresectable pancreatic cancer undergoing systemic chemotherapy	6
Ishii et al. 2017	Regorafenib is associated with increased skeletal muscle loss in gastrointestinal stromal tumor	2

Ito et al. 2020	Depletion of Psoas Muscle Mass after Systemic Chemotherapy Is Associated with Poor Prognosis in Patients with Unresectable Pancreatic Cancer	7
Iwai et al. 2021	Sarcopenia and Visceral Metastasis at Cabazitaxel Initiation Predict Prognosis in Patients With Castration-resistant Prostate Cancer Receiving Cabazitaxel Chemotherapy	7
Iwamoto et al 2021	Sarcopenia and visceral metastasis at cabazitaxel initiation predict prognosis in patients with castration-resistant prostate cancer receiving cabazitaxel chemotherapy	0
Iwamoto et al 2021	Prevalence of Sarcopenia in Colorectal Liver Metastases Varies According to Primary Tumor Location	2
J.e Waha et al. 2019	Body weight and composition changes, primary tumor side, and resection as survival predictors in metastatic colorectal cancer	2
Franko et al. 2018	Impact of postoperative skeletal muscle change on survival after resection of periampullary cancer	2
Lee etla. 2018	Role of body composition in early stage colorectal cancer (CRC) outcomes	2
Hopkins et al. 2017	Clinical implications of muscle mass and quality in early-stage colorectal cancer (CRC)	2
Hopkins et al. 2018	Body composition changes in stem cell transplantation: The case of lymphoma patients	2
Jabbour et al. 2017	Muscle wasting, visceral and subcutaneous adiposity, inflammation, nutritional deficiencies, and metastatic esophageal cancer (MEC) prognosis	2
Bajwa et al. 2019	Muscle density loss during cancer therapy for advanced endometrial cancer portends poor survival	2
Lee et al. 2019	Skeletal muscle depletion predicts the prognosis of patients with hepatocellular carcinoma treated with radiotherapy	7
Lee et al. 2018	Sarcopenia in patients (pts) with hepatocelluar carcinoma (HCC)	2
Meza-Junco et al. 2011	A prospective trial to validate appropriate functional or pro endpoints in evaluating cancer cachexia (CC) / sarcopenia in patients with non-small cell lung cancer (NSCLC)	2
Galeas et al. 2015	Visceral abdominal fat measured by computer tomography as a prognostic factor for gynecological malignancies?	6
Nattenmuller et al. 2018	Capecitabine (cape) dosing using skeletal muscle index (SMI) compared to body surface area (BSA)	2
Sun et al. 2016	Impact of Sarcopenia Using Normalized Total Psoas Area as a Surrogate on Overall Survival and Recurrence in Early Stage NSCLC Patients Treated with Stereotactic Body Radiotherapy	2
Taylor et al. 2019	Psoas muscle area is not representative of total skeletal muscle area in the assessment of sarcopenia in ovarian cancer	0

Ubachs et al. 2017	Loss of skeletal muscle mass during neoadjuvant chemotherapy and the relation to survival in patients with ovarian cancer; a prospective analysis of the ovhipec-1 cohort	2
Ubachs et al. 2019	Loss of skeletal muscle mass during neoadjuvant treatments correlates with worse prognosis in esophageal cancer: A retrospective cohort study	0
Jarvinen et al. 2018	Loss of skeletal muscle mass during neoadjuvant treatments correlates with worse prognosis in esophageal cancer: a retrospective cohort study	0
Jarvinen et al. 2018	Presence of Sarcopenia and Its Rate of Change Are Independently Associated with Long-term Mortality in Patients with Liver Cirrhosis	3
Jeong et al. 2018	A study on the correlation between body composition and durability of chemotherapy; A prospective cohort study	2
Ji et al. 2016	Prognostic significance of cachexia in advanced non-small cell lung cancer patients treated with pembrolizumab	5
Jo et al. 2021	Skeletal muscle and adipose tissue changes in the first phase of treatment of pediatric solid tumors	0
Joffe et al. 2020	Sarcopenia and Myosteatosis as Prognostic Markers in Patients with Advanced Cholangiocarcinoma Undergoing Palliative Treatment	6
Jordens et al. 2021	Prognostic value of body composition on recurrence and survival of advanced-stage head and neck cancer	0
Jung et al. 2019	Sarcopenia, related to neoadjuvant chemotherapy and perioperative outcomes, in resected gastric cancer	2
Mirkin et al. 2016	The serum levels of myostatin, follistatin, and IL-6 in hepatocellular carcinoma : Their association with sarcopenia and survival	1
Choi et al. 2018	The clinical impact of sarcopenia on chemotherapy delivery for gastric and esophageal cancers	2
Geddes et al. 2016	Postoperative Skeletal Muscle Loss Predicts Poor Prognosis of Adenocarcinoma of Upper Stomach and Esophagogastric Junction	0
Kudou et al. 2018	Relationship between perioperative change of total psoas muscle area and cancer prognosis in esophageal carcinoma	2
Matsui et al. 2020	Negative impact of cachexia during chemotherapy on survival as first-line chemotherapy for metastatic colorectal cancer	2
Nozawa et al. 2020	Effect of free amino acids mix with medium triglycerides and Withania somnifera on cancer cachexia	2

Gaddam et al. 2019	PSOAS VERSUS WHOLE L3 SKELETAL MUSCLE CROSS-SECTIONAL AREA: HOW DO THEY RELATE?	2
Rollins et al. 2019	Pre-chemotherapy functional and body status of patients with advanced gastrointestinal cancer in comparison to breast cancer patiens and healthy women	2
Stuecher et al. 2016	Pre-therapy physical function and body status of patients with advanced gastrointestinal cancer compared to breast cancer patients and healthy women	2
Stuecher et al. 2016	Prognostic significance of sarcopenia in metastatic esophageal squamous cell carcinoma	2
Taylor et al. 2019	The association between sarcopenia and cellular senescence of cancer associated fibroblast in pancreatic cancer	2
Yamamura et al. 2017	Differences in skeletal muscle loss caused by cytotoxic chemotherapy and molecular targeted therapy in patients with advanced non-small cell lung cancer	0
Kakinuma et al. 2018	Prognostic significance of osteopenia in patients with colorectal cancer: A retrospective cohort study	7
Kamada et al. 2021	Association of skeletal muscle loss with the long-term outcomes of esophageal cancer patients treated with neoadjuvant chemotherapy	6
Kamitani et al. 2019	Skeletal Muscle Mass Depletion After Gastrectomy Negatively Affects the Prognosis of Patients With Gastric Cancer	0
Kanazawa et al. 2020	Association between skeletal muscle loss and the response to nivolumab immunotherapy in advanced gastric cancer patients	6
Kano et al. 2021	A low psoas muscle index before treatment can predict a poorer prognosis in advanced bladder cancer patients who receive gemcitabine and nedaplatin therapy	7
Kasahara et al. 2017	A Low Psoas Muscle Index before Treatment Can Predict a Poorer Prognosis in Advanced Bladder Cancer Patients Who Receive Gemcitabine and Nedaplatin Therapy	6
Kasahara et al. 2017	Impact of sarcopenia and body composition change in patients with unresectable pancreatic cancer receiving systemic chemotherapy	0
Kawahira et al. 2019	Decreases in the Psoas Muscle Index Correlate More Strongly with Survival than Other Prognostic Markers in Esophageal Cancer After Neoadjuvant Chemoradiotherapy Plus Esophagectomy	0
Kawakita et al. 2020	The Influence of Sarcopenia on High-Risk Neuroblastoma	0
Kawakubo et al. 2019	Three cachexia phenotypes and the impact of fat-only loss on survival in FOLFIRINOX therapy for pancreatic cancer	0
Kays et al. 2018	Thoracic skeletal muscle loss is prognostic in malignant pleural mesothelioma	2

Kidd et al. 2021	Impact of sarcopenia on chemotherapy-triggered exacerbation of interstitial lung disease in patients with non-small cell lung cancer	6
Kikuchi et al. 2021	Clinical impact of sarcopenia on chemotherapy-triggered exacerbation of interstitial lung disease in patients with non-small cell lung cancer	2
Kikuchi et al. 2021	Prognostic significance of cachexia score assessed by CT in male patients with small cell lung cancer	6
Kim et al. 2018	Prognostic Significance of CT-Determined Sarcopenia in Patients with Small-Cell Lung Cancer	6
Kim et al. 2015	Clinical significance of skeletal muscle density and sarcopenia in patients with pancreatic cancer undergoing first-line chemotherapy: a retrospective observational study	0
Kim et al. 2021	Clinical significance of skeletal muscle density and sarcopenia in patients with pancreatic cancer undergoing first-line chemotherapy: a retrospective observational study	0
Kim et al. 2021	Preoperative nutritional risk index and postoperative one-year skeletal muscle loss can predict the prognosis of patients with gastric adenocarcinoma: a registry-based study	0
Kim et al. 2021	Effect on survival of skeletal muscle mass changes in patients with locally advanced pancreatic cancer receiving FOLFIRINOX	0
Kim et al. 2018	EFFECT ON SURVIVAL OF SKELETAL MUSCLE MASS CHANGES IN PATIENTS WITH LOCALLY ADVANCED PANCREATIC CANCER RECEIVING FOLFIRINOX	0
Kim et al. 2018	Effect on survival of skeletal muscle mass changes in patients with locally advanced pancreatic cancer receiving FOLFIRINOX	2
Kim et al. 2018	Effect on Survival of Skeletal Muscle Mass Changes in Patients with Locally Advanced Pancreatic Cancer Receiving Folfirinox	0
Kim et al. 2018	Prognostic implications of body composition change during primary treatment in patients with ovarian cancer: A retrospective study using an artificial intelligence-based volumetric technique	2
Kim et al. 2021	Prognostic significance of CT-determined sarcopenia in patients with advanced gastric cancer treated with chemotherapy	2
Kim et al. 2018	Prognostic significance of sarcopenia in microsatellite-stable gastric cancer patients treated with programmed death-1 inhibitors	6
Kim et al. 2021	Impact of Body Composition Status on 90-Day Mortality in Cancer Patients with Septic Shock: Sex Differences in the Skeletal Muscle Index	6

Kim et al. 2019	Sarcopenia as a predictor of post-transplant tumor recurrence after living donor liver transplantation for hepatocellular carcinoma beyond the Milan criteria	6
Kim et al. 2018	Doxorubicin Combined With Ifosfamide for Sarcoma Induces Muscle Atrophy and Sleep Disruption	0
Kinoshita et al. 2021	Predictors of physical and functional loss in lung cancer patients receiving chemotherapy	2
Kinsey et al. 2017	Predictors of Physical and Functional Loss in Advanced-Stage Lung Cancer Patients Receiving Platinum Chemotherapy	7
Kinsey et al. 2018	Early Skeletal Muscle Loss in Non-Small Cell Lung Cancer Patients Receiving Chemoradiation and Relationship to Survival	0
Kiss et al. 2019	Sarcopenia Affects Systemic and Local Immune System and Impacts Postoperative Outcome in Patients with Extrahepatic Cholangiocarcinoma	6
Kitano et al. 2019	Sarcopenia Is an Important Prognostic Factor in Patients With Cervical Cancer Undergoing Concurrent Chemoradiotherapy	7
Kiyotoki et al. 2018	Rapidly declining skeletal muscle mass predicts poor prognosis of hepatocellular carcinoma treated with transcatheter intra-arterial therapies	6
Kobayashi et al. 2018	Prognostic value of subcutaneous adipose tissue volume in hepatocellular carcinoma treated with transcatheter intra-arterial therapy	6
Kobayashi et al. 2018	Sarcopenia as a prognostic factor for survival in patients with locally advanced gastroesophageal adenocarcinoma	6
Koch et al. 2019	Is sarcopenia and sarcopenic obesity associated with clinical and pathological outcomes in patients undergoing radical nephroureterectomy?	6
Kocher et al. 2018	Skeletal muscle loss during anti-EGFR combined chemotherapy regimens predicts poor prognosis in patients with RAS wild metastatic colorectal cancer	0
Köstek et al. 2019	Muscle radiodensity and mortality in patients with colorectal cancer	6
Kroenke et al. 2018	Skeletal muscle loss and prognosis of breast cancer patients	0
Kubo et al. 2017	Postoperative Skeletal Muscle Loss Predicts Poor Prognosis of Adenocarcinoma of Upper Stomach and Esophagogastric Junction	0
Kudou et al. 2019	Postoperative development of sarcopenia is a strong predictor of a poor prognosis in patients with adenocarcinoma of the esophagogastric junction and upper gastric cancer	0
Kudou et al. 2019	Loss of skeletal muscle mass after curative gastrectomy is a poor prognostic factor	0
Kugimiya et al. 2018	Sarcopenia is a reliable prognostic factor in patients with advanced pancreatic cancer receiving FOLFIRINOX chemotherapy	6

Kurita et al. 2019	Sarcopenia is a reliable prognostic factor in patients with advanced pancreatic cancer receiving FOLFIRINOX chemotherapy	6
Kurita et al. 2019	Impact of different palliative systemic treatments on skeletal muscle mass in metastatic colorectal cancer patients	0
Kurk et al. 2018	Loss of skeletal muscle index and survival in patients with metastatic colorectal cancer: Secondary analysis of the phase 3 CAIRO3 trial	0
Kurk et al. 2020	Trajectory of body mass and skeletal muscle indices and disease progression in metastatic colorectal cancer patients	0
Kurk et al. 2019	Impact of skeletal muscle index (SMI) loss during palliative systemic treatment (Tx) on time to progression and overall survival (OS) in metastatic colorectal cancer (mCRC) patients.	2
Kurk et al. 2017	Skeletal muscle mass loss and dose-limiting toxicities in metastatic colorectal cancer patients	0
Kurk et al. 2019	Relationship between muscle mass and quality of life in breast cancer patients who underwent chemotherapy	0
Kurniawan et al. 2020	Relationship between low muscle mass pre-chemotherapy with hematological toxicity in breast cancer patients after 3 cycles of chemotherapy	2
Kurniawan et al. 2021	Core muscle index is prognostic of survival in advanced ovarian cancers	2
Conrad et al. 2016	Longitudinal changes in body composition during ipilimumab treatment for metastatic melanoma	2
Daly et al. 2015	Skeletal muscle mass as a prognostic indicator in resected pancreatic cancer patients	2
Jongchan et al. 2017	Opportunistic Measurement of Skeletal Muscle Size and Muscle Attenuation on Computed Tomography Predicts 1-Year Mortality in Medicare Patients	3
Lenchik et al. 2018	Cancer cachexia in the age of obesity: Skeletal muscle depletion is a powerful prognostic factor, independent of body mass index	2
Martin et al. 2013	Body Composition Is an Independent Predictor of Outcome in Patients with Hepatocellular Carcinoma Treated with Sorafenib	6
Labeur et al 2019	Sarcopenia is an independent prognostic factor in elderly patients with diffuse large B-cell lymphoma treated with immunochemotherapy	6
Lanic et al. 2014	Prognostic significance of sarcopenia and decreased relative dose intensity during the initial two cycles of first-line sunitinib for metastatic renal cell carcinoma	7
Lee et al. 2021	Sarcopena as a predicting factors for chemotherapy in advanced breast cancer patients	2

Lee et al. 2019	Skeletal Muscle Mass Predicts Poor Prognosis in Patients with Advanced Pancreatic Cancer Undergoing Second-Line FOLFIRINOX Chemotherapy	0
Lee et al. 2019	Skeletal Muscle Mass Predicts Poor Prognosis in Patients with Advanced Pancreatic Cancer Undergoing Second-Line FOLFIRINOX Chemotherapy	0
Lee et al. 2019	Longitudinal changes in skeletal muscle mass in patients with advanced squamous cell lung cancer	7
Lee et al. 2021	Postoperative muscle mass restoration as a prognostic factor in patients with resected pancreatic cancer	0
Lee et al. 2020	Muscle radiodensity loss during cancer therapy is predictive for poor survival in advanced endometrial cancer	7
Lee et al. 2019	Progressive muscle loss is an independent predictor for survival in locally advanced oral cavity cancer: A longitudinal study	0
Lee et al. 2021	Sarcopenia and Systemic Inflammation Synergistically Impact Survival in Oral Cavity Cancer	6
Lee et al. 2020	Skeletal Muscle Loss Is an Imaging Biomarker of Outcome after Definitive Chemoradiotherapy for Locally Advanced Cervical Cancer	0
Lee et al. 2018	Muscle Loss after Chemoradiotherapy as a Biomarker of Distant Failures in Locally Advanced Cervical Cancer	7
Lee et al. 2020	Muscle radiodensity loss during cancer therapy is predictive for poor survival in advanced endometrial cancer	0
Lee et al. 2019	Prognostic significance of CT-determined sarcopenia in patients with advanced gastric cancer	6
Lee et al. 2018	Subcutaneous Fat Distribution is a Prognostic Biomarker for Men with Castration Resistant Prostate Cancer	6
Lee et al. 2018	Association Between Sarcopenia and Metabolic Syndrome in Cancer Survivors	6
Lee et al. 2017	Sarcopenia in cancer survivors is associated with increased cardiovascular disease risk	6
Lee et al. 2018	Quantitative muscle mass biomarkers are independent prognosis factors in primary central nervous system lymphoma: The role of L3-skeletal muscle index and temporal muscle thickness	6
Leone et al. 2021	Toxicity of induction chemotherapy in head and neck cancer: The central role of skeletal muscle mass	7
Lere-Chevaleyre et al. 2021	Muscle wasting and survival following pre-operative chemoradiotherapy for locally advanced rectal carcinoma	7

Levolger et al. 2018	[The influence of cachexia on the immunotherapy efficacy of Sintilimab for non-small cell lung cancer]	1
Li et al. 2021	Predictive value of pancreatic dose-volume metrics on sarcopenia rate in gastric cancer patients treated with adjuvant chemoradiotherapy	7
Li et al. 2019	Muscle Mass, Density, and Strength Are Necessary to Diagnose Sarcopenia in Patients With Gastric Cancer	6
Lin et al. 2019	The geriatric syndrome of sarcopenia impacts allogeneic hematopoietic cell transplantation outcomes in older lymphoma patients	0
Lin et al. 2020	Decreased skeletal muscle mass after neoadjuvant therapy correlates with poor prognosis in patients with esophageal cancer	7
Liu et al. 2016	Decreased Skeletal Muscle Mass After Neoadjuvant Therapy Correlates with Poor Prognosis in Patients with Esophageal Cancer	0
Liu et al. 2016	Skeletal Muscle Composition Predicts Outcome in Critically Ill Patients	6
Loosen et al. 2020	Progressive sarcopenia correlates with poor response and outcome to immune checkpoint inhibitor therapy	0
Loosen et al. 2021	Circulating Activin A predicts survival in cancer patients	6
Loumaye et al. 2017	Prognostic impact of psoas muscle index in patients with diffuse large B-cell lymphoma might be dependent on the immunochemotherapy type	2
Lucijanić et al. 2021	More Pronounced Muscle Loss During Immunochemotherapy is Associated with Worse Clinical Outcomes in Newly Diagnosed Patients with Diffuse Large B-Cell Lymphoma with Unfavorable Features	7
Lucijanić et al. 2021	ABCL-018: Muscle Loss During Immunochemotherapy for Diffuse Large B-Cell Lymphoma and its Clinical and Prognostic Associations	2
Lucijanić et al. 2021	Sarcopenia and Response to Neoadjuvant Chemotherapy for Muscle-Invasive Bladder Cancer	0
Lyon et al. 2019	The impact of inflammatory biomarkers, BMI, and sarcopenia on survival in advanced hepatocellular carcinoma treated with immunotherapy	2
Akce et al. 2020	Fat quality: The handsome stranger in esophageal cancer prognosis	2
Anciaux et al. 2018	The impact of skeletal muscle and adipose tissue on long-term survival in patients with resectable colorectal cancer	2
Sawyer et al. 2018	Sarcopenia affects treatment toxicity in metastatic colorectal cancer patients: Results of a prospective multicenter study	2

Barret et al. 2012	Effect of cancer cachexia on treatment toxicity in metastatic colorectal cancer patients: Results of a prospective multicenter study	2
Barret et al. 2012	Baseline skeletal muscle index (SMI) values are associated with biomarkers of insulin resistance in stage IV non-small cell lung cancer	2
Batus et al. 2016	Obesity paradox in cancer: New insights provided by body composition	6
Gonzalez et al. 2014	Can sarcopenia be used as a prognostic indicator in patients with metastatic penile cancer?	2
Christodoulidou et al. 2016	Sarcopenia as a prognostic factor in penile cancer patients: Assessment of body composition in patients with advanced penile cancer	2
Christodoulidou et al. 2016	The association between sarcopenic overweight and chemotherapy toxicity in Bile Duct and Gallbladder cancer patients treated with Gemcitabine and Cisplatin	2
Gaspersz et al. 2018	Association between changing body composition after diagnosis and survival of patients with advanced pancreatic cancer	2
Rosenthal et al. 2018	Changes in skeletal muscle index and body mass are prognostic factors in first line stage IV non-small cell lung cancer (NCSCL) patients	2
Fidler et al. 2015	Impact of sarcopenia and body composition change in patients with unresectable pancreatic cancer receiving systemic chemotherapy	2
Kawahira et al. 2019	Prognostic impact of cancer cachexia in patients with advanced non-small cell lung cancer	0
Kimura et al. 2014	Change in body composition and survival in patients with pancreatic cancer	2
Krishnan et al. 2019	The effects of kinase inhibitors on body composition in endocrine tumor patients-pilot study	2
Kutahyalioglu et al. 2017	Postoperative psoas muscle loss and nutritional deterioration after radical cystectomy for patients with invasive bladder cancer	2
Miyake et al. 2017	Changes in skeletal muscle mass during PD-1 and PD-L1 checkpoint inhibitor therapy in advanced-stage non-small cell lung cancer patients	2
Multani et al. 2019	Skeletal muscle density is independently prognostic of outcomes in newly diagnosed mantle cell lymphoma patients: Post hoc analysis of LYM 3002	2
Chu et al. 2015	ARE BODY COMPOSITION PARAMETERS ASSOCIATED WITH THE CLINICAL OUTCOME OF PATIENTS WITH ADVANCED PANCREATIC CANCER RECEIVING FLUOROPYRIMIDINE-BASED CHEMOTHERAPY?	2
Som et al. 2019	The influence of the change of body composition after neoadjuvant treatment on outcome after pancreaticoduodenectomy for pancreatic cancer	2

Takahashi et al. 2015	Relationship Between Sarcopenia and Prognosis in Patient With Concurrent Chemo-Radiation Therapy for Esophageal Cancer	7
Ma et al. 2019	Prognostic role of sarcopenia in metastatic colorectal cancer patients during first-line chemotherapy: A retrospective study	6
Maddalena et al. 2021	P-272 Sarcopenia in metastatic colorectal cancer patients during first-line chemotherapy	2
Maddalena et al. 2020	P-272 Sarcopenia in metastatic colorectal cancer patients during first-line chemotherapy	2
Maddalena et al. 2020	Skeletal muscle loss in the postoperative acute phase after esophageal cancer surgery as a new prognostic factor	0
Maeda et al. 2020	Influence of body composition profile on outcomes following colorectal cancer surgery	6
Malietzis et al. 2016	"Sarcopenia and intramuscular fat deposition are associated with poor survival in Indonesian patients with hepatocellular carcinoma: a retrospective study"	6
Mardian et al. 2019	Sarcopenia as a Predictive Factor for Response to Upfront Cisplatin-Based Chemotherapy in Patients with Muscle-Invasive Urothelial Bladder Cancer	0
Mari et al. 2018	Sarcopenia as a Predictive Factor for Response to Upfront Cisplatin-Based Chemotherapy in Patients with Muscle-Invasive Urothelial Bladder Cancer	5
Mari et al. 2018	Body composition in long-term survivors of acute lymphoblastic leukemia diagnosed in childhood and adolescence: A focus on sarcopenic obesity	6
Marriott et al. 2018	Cancer cachexia in the age of obesity: skeletal muscle depletion is a powerful prognostic factor, independent of body mass index	6
Martin et al. 2013	Cancer-Associated Malnutrition and CT-Defined Sarcopenia and Myosteatosis Are Endemic in Overweight and Obese Patients	6
Martin et al. 2020	Impact of skeletal muscle mass in patients with unresectable gastric cancer who received palliative first-line chemotherapy based on 5-fluorouracil	6
Matsunaga et al. 2021	Impact of skeletal muscle mass in patients with unresectable gastric cancer who received palliative first-line chemotherapy based on 5-fluorouracil	7
Matsunaga et al. 2021	Correlation between Skeletal Muscle Mass and Adverse Events of Neoadjuvant Chemotherapy in Patients with Gastric Cancer	0
Matsuura et al. 2020	Cancer cachexia syndrome in the prediction of outcome in patients with metastatic non-small cell lung cancer (NSCLC) treated with immune checkpoint inhibitors (ICIs): Results from a singleinstitution, prospective, observational study	2
Mavroudis et al. 2020	Negative Impact of Skeletal Muscle Wasting After Neoadjuvant Chemotherapy Followed by Surgery on Survival for Patients with Thoracic Esophageal Cancer	0

Mayanagi et al. 2017	Negative Impact of Skeletal Muscle Wasting After Neoadjuvant Chemotherapy Followed by Surgery on Survival for Patients with Thoracic Esophageal Cancer	0
Mayanagi et al. 2017	Lean body mass wasting and toxicity in early breast cancer patients receiving anthracyclines	7
Mazzuca et al. 2018	Meeting Minimum ESPEN Energy Recommendations Is Not Enough to Maintain Muscle Mass in Head and Neck Cancer Patients	0
McCurdy et al. 2019	Prognostic Impact of Sarcopenia in Patients With Biliary Tract Cancer Undergoing Chemotherapy	6
Meguro et al. 2021	Prognostic impact of sarcopenia in patients with biliary tract cancer undergoing chemotherapy	0
Meguro et al. 2021	Effects of sarcopenia and background factors on elderly biliary cancer patients receiving chemotherapy	2
Meguro et al. 2019	Worsening central sarcopenia and increasing intra-abdominal fat correlate with decreased survival in patients with adrenocortical carcinoma	6
Miller et al. 2012	Sarcopenia and Visceral Adiposity Are Not Independent Prognostic Markers for Extensive Disease of Small-Cell Lung Cancer: A Single-Centered Retrospective Cohort Study	6
Minami et al. 2020	Sarcopenia predicts early dose-limiting toxicities and pharmacokinetics of sorafenib in patients with hepatocellular carcinoma	6
Mir et al. 2012	Incidence and frequency of cancer cachexia during chemotherapy for advanced pancreatic ductal adenocarcinoma	5
Mitsunaga et al. 2020	Incidence and frequency of cancer cachexia during chemotherapy for advanced pancreatic ductal adenocarcinoma	5
Mitsunaga et al. 2019	Assessment of nutritional status and changes in lean body weight in patients with head and neck cancer undergoing treatment	2
Mittal et al. 2014	Clinical utility of bioelectrical impedance analysis in patients with locoregional muscle invasive or metastatic urothelial carcinoma: a subanalysis of changes in body composition during neoadjuvant systemic chemotherapy	0
Miyake et al. 2018	Clinical impact of postoperative loss in psoas major muscle and nutrition index after radical cystectomy for patients with urothelial carcinoma of the bladder	0
Miyake et al. 2017	The association of skeletal muscle mass and cisplatin pharmacokinetics in head and neck cancer patients: The prospective PLATISMA study	2
Molenaar-Kuijsten et al. 2021	Worse capecitabine treatment outcome in patients with a low skeletal muscle mass is not explained by altered pharmacokinetics	7

Molenaar-Kuijsten et al. 2021	Detection and evaluation of the role of sarcopenia in elderly patients with cancer treated with chemotherapy. ONCOSARCO project	0
Molina Garrido et al. 2017	Correlation between muscle mass and handgrip strength in digestive cancer patients undergoing chemotherapy	6
Moreau et al. 2019	Correlation between muscle mass and handgrip strength in digestive cancer patients undergoing chemotherapy	0
Moreau et al. 2019	Impact of cancer cachexia on the therapeutic outcome of combined chemoimmunotherapy in patients with non-small cell lung cancer: a retrospective study	5
Morimoto et al. 2021	Impact of cancer cachexia on the therapeutic outcome of combined chemoimmunotherapy in patients with non-small cell lung cancer: a retrospective study	0
Morimoto et al. 2021	Reversible sarcopenia in patients with gastrointestinal stromal tumor treated with imatinib	0
Moryoussef et al. 2015	Computed tomography at C3 level and dynamometry as techniques for the diagnosis of sarcopenia in head and neck cancer patients	1
Muresan et al. 2020	Loss of adipose tissue and plasma phospholipids: relationship to survival in advanced cancer patients	5
Murphy et al. 2010	Low skeletal muscle density is associated with poor survival in patients who receive chemotherapy for metastatic gastric cancer	6
Hayashi et al. 2015	Poor prognosis linked to reduced muscle mass in hemodialysis patients is mainly confined to elderly men	2
Isoyama et al. 2016	PSOAS MUSCLE INDEX AFFECTS THE LONG-TERM PROGNOSIS AFTER ESD FOR ELDERLY GASTRIC CANCER PATIENTS OVER 80 YEARS OLD	2
Ito et al. 2019	Influence of sarcopenia in high-risk neuroblastoma	0
Kawakubo et al. 2017	Skeletal muscle depletion and relationship to survival in non-small cell lung cancer patients receiving chemo-radiation	2
Kiss et al. 2016	PROGNOSTIC SIGNIFICANCE OF MUSCLE DEPLETION IN END-STAGE CANCER PATIENTS	2
Mori et al. 2019	Prognosis value of psoas major muscle evaluated on CT scan in terminal ill cancer patients	2
Mori et al. 2014	Neoadjuvant chemotherapy results in skeletal muscle index decline in patients with muscle-invasive bladder cancer	2
Steinsiek et al. 2018	The impact of an intensive nutritional support programme during neoadjuvant chemotherapy for upper gastrointestinal cancer	2
Tewari et al. 2014	Skeletal Muscle Mass Reduction Velocity as a Simple Prognostic Indicator for Patients with Metastatic Urothelial Carcinoma Receiving Second-Line Chemotherapy	6

Nagai et al. 2019	Association between Skeletal Muscle Depletion and Sorafenib Treatment in Male Patients with Hepatocellular Carcinoma: A Retrospective Cohort Study	6
Naganuma et al. 2017	Impact of reduced skeletal muscle volume on clinical outcome after esophagectomy for esophageal cancer: A retrospective study	0
Nagata et al. 2018	Risk factors for progressive sarcopenia 6 months after complete resection of lung cancer: what can thoracic surgeons do against sarcopenia?	0
Nagata et al. 2020	Effect of progressive sarcopenia during postoperative 6 months on long-term prognosis of completely resected lung cancer	0
Nagata et al. 2019	[Impact of Muscle Mass Reduction on Postoperative Adjuvant Chemotherapy and Long-Term Prognosis in Patients with Gastric Cancer]	1
Nagata et al. 2018	Skeletal muscle depletion during chemotherapy has a large impact on physical function in elderly Japanese patients with advanced non-small-cell lung cancer	0
Naito et al. 2017	Alteration of muscle mass after chemotherapy in patients with newly diagnosed multiple myeloma	2
Nakamura et al. 2018	Alteration of muscle mass after chemotherapy in patients with newly diagnosed multiple myeloma	0
Nakamura et al. 2018	Skeletal Muscle Loss After Esophagectomy Is an Independent Risk Factor for Patients with Esophageal Cancer	0
Nakashima et al. 2020	Prognostic impact of CT-quantified muscle and fat distribution before and after first-line-chemotherapy in lung cancer patients	7
Nattenmuller et al. 2017	Outcome after neoadjuvant chemoradiation and correlation with nutritional status in patients with locally advanced pancreatic cancer	5
Naumann et al. 2013	Cachectic Body Composition and Inflammatory Markers Portend a Poor Prognosis in Patients with Locally Advanced Pancreatic Cancer Treated with Chemoradiation	0
Naumann et al. 2019	Computed tomography diagnosed cachexia and sarcopenia in 725 oncology patients: is nutritional screening capturing hidden malnutrition?	6
Ní Bhuachalla et al. 2018	Effect of psoas muscle mass after endoscopic therapy for patients with esophageal varices	6
Nishikawa et al. 2017	Unfavorable impact of decreased muscle quality on the efficacy of immunotherapy for advanced non-small cell lung cancer	6
Nishioka et al. 2021	A lower psoas muscle volume was associated with a higher rate of recurrence in male clear cell renal cell carcinoma	6

Noguchi et al. 2020	Change in skeletal muscle index and its prognostic significance in patients who underwent successful conversion therapy for initially unresectable colorectal cancer: observational study	0
Nozawa et al. 2020	Myosteatosis in a systemic inflammation-dependent manner predicts favorable survival outcomes in locally advanced esophageal cancer	6
Gabiatti et al. 2019	Skeletal muscle loss during chemotherapy and its association with survival and systemic treatment toxicity in metastatic colorectal cancer: An AGEO prospective multicenter study	0
Gallois et al. 2021	The impact of sarcopenia on tolerance of radiation and outcome in patients with head and neck cancer receiving chemoradiation	6
Ganju et al. 2019	The impact of skeletal muscle abnormalities on tolerance to adjuvant chemotherapy and radiation and outcome in patients with endometrial cancer	6
Ganju et al. 2020	Disentangling the body weight-bone mineral density association among breast cancer survivors: an examination of the independent roles of lean mass and fat mass	6
George et al. 2013	Sarcopenia and inflammation are independent predictors of survival in male patients newly diagnosed with small cell lung cancer	6
Go et al. 2016	Prognostic impact of sarcopenia in patients with diffuse large B-cell lymphoma treated with rituximab plus cyclophosphamide, doxorubicin, vincristine, and prednisone	6
Go et al. 2016	Characterising the impact of body composition change during neoadjuvant chemotherapy for pancreatic cancer	0
Griffin et al. 2019	Skeletal muscle depletion is associated with disease progression during neo-adjuvant therapy for borderline resectable pancreatic adenocarcinoma	2
Griffin et al. 2017	Body composition change during chemotherapy for borderline resectable pancreatic cancer	2
Griffin et al. 2019	Associations between severe co-morbidity and muscle measures in advanced non-small cell lung cancer patients	7
Grønberg et al. 2019	A comparison of CT based measures of skeletal muscle mass and density from the Th4 and L3 levels in patients with advanced non-small-cell lung cancer	6
Grønberg et al. 2019	Association of Body Composition With Survival and Locoregional Control of Radiotherapy-Treated Head and Neck Squamous Cell Carcinoma	7
Grossberg et al. 2016	Sarcopenia during neoadjuvant therapy for oesophageal cancer: characterising the impact on muscle strength and physical performance	7
Guinan et al. 2018	Body Composition as a Predictor of Toxicity and Prognosis in Patients with Diffuse Large B-Cell Lymphoma Receiving R-CHOP Immunochemotherapy	7

Guo et al. 2021	Sarcopenia as a prognostic biomarker of metastatic renal cell carcinoma: Its effect on survival benefit from cytoreductive nephrectomy	2
Fukushima et al. 2015	Prognostic significance of sarcopenia in metastatic renal cell carcinoma: Its association with survival benefit from cytoreductive nephrectomy	2
Fukushima et al. 2015	Sarcopenia is a prognostic biomarker of advanced urothelial carcinoma	2
Fukushima et al. 2014	Tetra-modality bladder sparing therapy can be a viable treatment option for muscle-invasive bladder cancer patients with sarcopenia	2
Fukushima et al. 2019	Post-therapeutic recovery of skeletal muscle mass predicts favorable prognosis in advanced urothelial carcinoma patients receiving 1st-line platinum-based chemotherapy	2
Fukushima et al. 2017	Prognostic significance of sarcopenia in upper tract urothelial carcinoma patients who underwent radical nephroureterectomy	2
Fukushima et al. 2016	Postoperative recovery of skeletal muscle mass is associated with favorable prognosis in metastatic renal cell carcinoma patients who underwent cytoreductive nephrectomy	2
Fukushima et al. 2016	Impact of cancer cachexia on survival during chemotherapy in patients with upper gastrointestinal cancer	2
Goto et al. 2017	Prognostic impact of quantitative imaging analysis of lean body mass after chemoradiation therapy for patients with advanced nasopharyngeal cancer	2
Inokuchi et al. 2018	Effect of changes in skeletal muscle mass on oncological outcomes during first-line sunitinib therapy for metastatic renal cell carcinoma	0
Ishihara et al. 2019	Association of sarcopenia with metabolic syndrome in Korean cancer survivors	1
Kim et al. 2017	Clinical relevance of cachexia assessed by an anthropometric tool in elderly patients with diffuse large b-cell lymphoma treated by immunochemotherapy	2
Lanic et al. 2013	Sarcopenia determined by computed tomography imaging is an independant prognostic factor in elderly patients with diffuse large B CELL lymphoma treated by immunochemotherapy	2
Lanic et al. 2012	Sarcopenia is associated with postoperative complication after pancreaticoduodenectomy	2
Lee et al. 2019	Staging of nutrition disorders in 531 non-small cell lung cancer (NSCLC) patients: Benefit from skeletal muscle mass, anorexia and performance status assessments	2
Morel et al. 2017	Significance of muscle mass decreasing on prognosis after pancreatic cancer surgery	2
Mori et al. 2018	Clinical impact of sarcopenia and skeletal muscle mass change during chemotherapy on outcomes of diffuse large b-cell lymphoma	2

Teranaka et al. 2017	Muscle wasting associated with poor outcome in patients with hepatocellular carcinoma undergoing sorafenib treatment	2
Ueki et al. 2016	Effect of muscle mass on toxicity and survival in patients with colon cancer undergoing adjuvant chemotherapy	6
H-W Jung et al. 2014	Morphometrics predicts overall survival in patients with lung, breast, prostate, or myeloma spine metastases, regardless of histology	2
Zakaria et al. 2018	Decrease in skeletal muscle index one year after radical cystectomy as a prognostic indicator in patients with urothelial bladder cancer	0
Yun-Sok Ha et al. 2019	Influence of body composition and muscle strength on outcomes after multimodal oesophageal cancer treatment	0
Hagens et al. 2020	The role of sarcopenia in patients with intrahepatic cholangiocarcinoma: Prognostic marker or hyped parameter?	6
Hahn et al. 2019	Associations between muscle measures, survival, and toxicity in patients with limited stage small cell lung cancer	6
Halvorsen et al. 2020	Prognostic and clinical impact of sarcopenia in esophageal squamous cell carcinoma	6
Harada et al. 2016	O16-2 Prognostic impacts of change in skeletal muscle mass during neoadjuvant chemotherapy in patients with esophageal cancer	2
Harada et al. 2021	Low skeletal muscle density is associated with poor survival in patients who receive chemotherapy for metastatic gastric cancer	6
Hayasi et al. 2016	Skeletal muscle loss during neoadjuvant chemotherapy is a predictor of major postoperative complications in patients with esophagus cancer	2
Higashizono et al. 2021	Nutritional status, cachexia, and anorexia in women with peritoneal metastasis and intraperitoneal chemotherapy: A longitudinal analysis	0
Hilal et al. 2017	Muscle volume loss as a prognostic marker in hepatocellular carcinoma patients treated with sorafenib	0
Hiraoka et al. 2017	Impact of muscle volume and muscle function decline in patients undergoing surgical resection for hepatocellular carcinoma	6
Hiraoka et al. 2018	Effect of physical activity on bone strength and body composition in breast cancer premenopausal women during endocrine therapy	4
Hojan et al. 2013	Evaluation of sarcopenia, sarcopenic obesity, and phase angle in geriatric gastrointestinal cancer patients: before and after chemotherapy	7

Hopanci et al. 2019	Change in Skeletal Muscle Following Resection of Stage I-III Colorectal Cancer is Predictive of Poor Survival: A Cohort Study	0
Hopkins et al. 2019	The Impact of Muscle and Adipose Tissue on Long-term Survival in Patients With Stage I to III Colorectal Cancer	6
Hopkins et al. 2019	Prognostic significance of the skeletal muscle index and an inflammation biomarker in patients with breast cancer who underwent postoperative adjuvant radiotherapy	6
Hua et al. 2019	Low Skeletal Muscle Mass Impairs Quality of Life in Nasopharyngeal Carcinoma Patients Treated With Concurrent Chemoradiotherapy	6
Hua et al. 2020	Association between sarcopenia and clinical outcomes in patients with esophageal cancer under neoadjuvant therapy	7
Huang et al. 2020	Muscle loss during primary debulking surgery and chemotherapy predicts poor survival in advanced-stage ovarian cancer	0
Huang et al. 2020	Association Between Sarcopenia and Clinical Outcomes in Patients With Esophageal Cancer Under Neoadjuvant Therapy	6
Huang et al. 2020	Value of Sarcopenia defined by the new EWGSOP2 consensus for the prediction of Postoperative Complications and Long-term Survival after Radical Gastrectomy for Gastric Cancer: A comparison with four common nutritional screening tools	6
Huang et al. 2020	Severe muscle loss during radical chemoradiotherapy for non-metastatic nasopharyngeal carcinoma predicts poor survival	7
Huang et al. 2019	Body Composition in Patients with Radioactive Iodine-Refractory, Advanced Differentiated Thyroid Cancer Treated with Sorafenib or Placebo: A Retrospective Analysis of the Phase III DECISION Trial	0
Hulliard et al. 2019	Changes of Skeletal Muscle Mass during Palliative Chemotherapy in Older Patients with Advanced Gastric Cancer	0
Hwang et al. 2019	Loss of skeletal muscle mass during palliative chemotherapy is a poor prognostic factor in patients with advanced gastric cancer	2
I.g Hwang et al. 2019	The prognostic impact of sarcopenic change after 8 weeks of 1st line gemcitabine based chemotherapy in advanced pancreatic adenocarcinoma	2
I-H Kim et al. 2018	Psoas muscle measurements are inferior to total skeletal muscle measurements in the assessment of sarcopenia in ovarian cancer	2
Rutten et al. 2017	Prognostic factors for risk stratification of patients with recurrent or metastatic pancreatic adenocarcinoma who were treated with gemcitabine-based chemotherapy	6

Park et al. 2016	Changes in skeletal muscle mass during neoadjuvant chemotherapy are related to survival in ovarian cancer	2
Rutten et al. 2015	Impact of nutritional derangement on treatment outcome in advanced non-small-cell lung cancer (A-NSCLC) patients (pts)	2
Trestini et al. 2019	Muscle mass in elderly patients with DLBCL treated with RCHOP	2
Vaxman et al. 2016	Prognostic impact of sarcopenia in patients with metastatic hormone-sensitive prostate cancer	6
Ikeda et al. 2020	Prognostic impact of the psoas muscle index, a parameter of sarcopenia, in patients with diffuse large B-cell lymphoma treated with rituximab-based chemoimmunotherapy	7
Iltar et al. 2021	Rapid Depletion of Subcutaneous Adipose Tissue during Sorafenib Treatment Predicts Poor Survival in Patients with Hepatocellular Carcinoma	0
Imai et al. 2020	Skeletal muscle depletion predicts the prognosis of patients with hepatocellular carcinoma treated with sorafenib	6
Imai et al. 2015	Rapid Depletions of Subcutaneous Fat Mass and Skeletal Muscle Mass Predict Worse Survival in Patients with Hepatocellular Carcinoma Treated with Sorafenib	7
Imai et al. 2019	Skeletal muscle depletion is an independent prognostic factor for hepatocellular carcinoma	6
Iritani et al. 2015	Is cardiac wasting accompanied by skeletal muscle loss in breast cancer patients receiving anticancer treatment?	2
Ishida et al. 2017	Quantity and Quality of Skeletal Muscle as an Important Predictor of Clinical Outcomes in Patients with Esophageal Cancer Undergoing Esophagectomy after Neoadjuvant Chemotherapy	6
Ishida 2021	Sarcopenia and the Modified Glasgow Prognostic Score are Significant Predictors of Survival Among Patients with Metastatic Renal Cell Carcinoma Who are Receiving First-Line Sunitinib Treatment	6
Ishihara et al. 2016	Effect of Changes in Skeletal Muscle Mass on Oncological Outcomes During First-Line Sunitinib Therapy for Metastatic Renal Cell Carcinoma	0
Ishihara et al. 2018	Effect of pretreatment psoas muscle mass on survival for patients with unresectable pancreatic cancer undergoing systemic chemotherapy	6
Ishii et al. 2017	Regorafenib is associated with increased skeletal muscle loss in gastrointestinal stromal tumor	2
Ito et al. 2020	Depletion of Psoas Muscle Mass after Systemic Chemotherapy Is Associated with Poor Prognosis in Patients with Unresectable Pancreatic Cancer	7

Iwai et al. 2021	Sarcopenia and Visceral Metastasis at Cabazitaxel Initiation Predict Prognosis in Patients With Castration-resistant Prostate Cancer Receiving Cabazitaxel Chemotherapy	7
Iwamoto et al. 2021	Sarcopenia and visceral metastasis at cabazitaxel initiation predict prognosis in patients with castration-resistant prostate cancer receiving cabazitaxel chemotherapy	0
Iwamoto et al. 2021	Prevalence of Sarcopenia in Colorectal Liver Metastases Varies According to Primary Tumor Location	2
J.e Waha et al. 2019	Body weight and composition changes, primary tumor side, and resection as survival predictors in metastatic colorectal cancer	2
Franko et al. 2018	Impact of postoperative skeletal muscle change on survival after resection of periampullary cancer	2
Lee et al. 2018	Role of body composition in early stage colorectal cancer (CRC) outcomes	2
Hopkins et al. 2017	Clinical implications of muscle mass and quality in early-stage colorectal cancer (CRC)	2
Hopkins et al. 2018	Body composition changes in stem cell transplantation: The case of lymphoma patients	2
Jabbour et al. 2017	Muscle wasting, visceral and subcutaneous adiposity, inflammation, nutritional deficiencies, and metastatic esophageal cancer (MEC) prognosis	2
Bajwa et al. 2019	Muscle density loss during cancer therapy for advanced endometrial cancer portends poor survival	2
Lee et al. 2019	Skeletal muscle depletion predicts the prognosis of patients with hepatocellular carcinoma treated with radiotherapy	7
Lee et al. 2018	Sarcopenia in patients (pts) with hepatocelluar carcinoma (HCC)	2
Meza-Junco et al. 2011	A prospective trial to validate appropriate functional or pro endpoints in evaluating cancer cachexia (CC) / sarcopenia in patients with non-small cell lung cancer (NSCLC)	2
Galeas et al. 2015	Visceral abdominal fat measured by computer tomography as a prognostic factor for gynecological malignancies?	6
Nattenmuller et al. 2018	Capecitabine (cape) dosing using skeletal muscle index (SMI) compared to body surface area (BSA)	2
Sun et al. 2016	Impact of Sarcopenia Using Normalized Total Psoas Area as a Surrogate on Overall Survival and Recurrence in Early Stage NSCLC Patients Treated with Stereotactic Body Radiotherapy	2
Talyor et al. 2019	Psoas muscle area is not representative of total skeletal muscle area in the assessment of sarcopenia in ovarian cancer	0
Ubachs et al. 2017	Loss of skeletal muscle mass during neoadjuvant chemotherapy and the relation to survival in patients with ovarian cancer; a prospective analysis of the ovhipec-1 cohort	2

Ubachs et al. 2019	Loss of skeletal muscle mass during neoadjuvant treatments correlates with worse prognosis in esophageal cancer: a retrospective cohort study	0
Järvinen et al. 2018	CT-based assessment of body composition following neoadjuvant chemohormonal therapy in patients with castration-naïve oligometastatic prostate cancer	0
Sheikhbahaei et al. 2021	Body Composition As A Predictor Of Critical Toxicities In Patients With Locoregionally Nasopharyngeal Carcinoma Undergoing Neoadjuvant Chemotherapy	2
Shen et al. 2020	No Deterioration in Clinical Outcomes of Carbon Ion Radiotherapy for Sarcopenia Patients with Hepatocellular Carcinoma	6
Shiba et al. 2018	A Retrospective Cohort Study to Investigate the Incidence of Cachexia During Chemotherapy in Patients with Colorectal Cancer	5
Shibata et al. 2020	Sarcopenia is associated with survival in patients with urothelial carcinoma treated with systemic chemotherapy	6
Shimizu et al. 2022	Effect of Muscle Mass Loss After Esophagectomy on Prognosis of Oesophageal Cancer	0
Shimoda et al. 2020	Impact of sarcopenia in patients with advanced non-small cell lung cancer treated with PD-1 inhibitors: A preliminary retrospective study	6
Shiroyama et al. 2019	Sarcopenia is not associated with morbidity, mortality, or recurrence after esophagectomy for cancer	6
Siegal et al. 2018	Pre-operative psoas major muscle size is negatively effected by neoadjuvant chemoradiotherapy in patients with rectal cancer	2
Simpson et al. 2019	Body mass index (BMI), albumin and skeletal muscle mass in non small cell lung carcinoma (NSCLC) patients before and after chemotherapy in persahabatan hospital Jakarta, Indonesia	2
Siregar et al. 2014	Low muscle mass is associated with chemotherapy-induced haematological toxicity in advanced non-small cell lung cancer	6
Sjoblom et al. 2015	Accelerated aging among childhood, adolescent, and young adult cancer survivors is evidenced by increased expression of p16(INK4a) and frailty	6
Smitherman et al. 2020	Muscle mass loss in patients with metastatic breast cancer	7
Solomayer et al. 2019	Skeletal muscle loss predicts oncological outcomes in T1HG patients treated with adjuvant intravesical BCG: Implications for decision-making?	2
Soria et al. 2020	Comparative Analysis Between Computed Tomography and Surrogate Methods to Detect Low Muscle Mass Among Colorectal Cancer Patients	6

Souza et al. 2019	Assessment of skeletal muscle mass as a predictive factor for chemotherapy toxicity and TTP in advanced nsclc patients with cancer cachexia	0
Srdic et al. 2017	Cancer cachexia, sarcopenia and biochemical markers in patients with advanced non-small cell lung cancer-chemotherapy toxicity and prognostic value	6
Srdic et al. 2016	Cancer cachexia, sarcopenia and biochemical markers in patients with advanced non-small cell lung cancer - Chemotherapy toxicity and prognostic value	0
Srdic et al. 2017	Sarcopenia as a predictor of survival and chemotoxicity in patients with epithelial ovarian cancer receiving platinum and taxane-based chemotherapy	6
Staley et al. 2020	Sarcopenia as a predictor of survival and chemotoxicity in patients with epithelial ovarian cancer receiving platinum and taxane-based chemotherapy	6
Staley et al. 2020	Loss of adipose tissue mass during chemotherapy predicts reduced survival in patients with colorectal cancer treated with palliative intent	2
Stella Sullivan et al. 2019	Changes in skeletal muscle mass during palliative chemotherapy in patients with advanced lung cancer	0
Stene et al. 2015	Evaluating Potential Biomarkers of Cachexia and Survival in Skeletal Muscle of Upper Gastrointestinal Cancer Patients	5
Stephens et al. 2015	The impact of sarcopenia and obesity on the tolerability of neoadjuvant chemotherapy in patients with muscle invasive bladder cancer	2
Stout et al. 2021	Combination of psoas muscle mass index and neutrophil/lymphocyte ratio as a prognostic predictor for patients undergoing nonsurgical hepatocellular carcinoma therapy	6
Sugama et al. 2021	Relationships among body composition, muscle strength, and sarcopenia in esophageal squamous cell carcinoma patients	6
Sugawara et al. 2020	Baseline Sarcopenia and Skeletal Muscle Loss During Chemotherapy Affect Survival Outcomes in Metastatic Gastric Cancer	0
Sugiyama et al. 2018	Respiratory strength and pectoralis muscle mass as measures of sarcopenia: Relation to outcomes in resected non-small cell lung cancer	6
Sun et al. 2020	Prognostic significance of low pectoralis muscle mass on preoperative chest computed tomography in localized non-small cell lung cancer after curative-intent surgery	6
Sun et al. 2020	Sarcopenia in patients with normal body mass index is an independent predictor for postoperative complication and long-term survival in gastric cancer	6
Sun et al. 2020	Sarcopenia after induction therapy in childhood acute lymphoblastic leukemia: its clinical significance	7

Suzuki et al. 2018	Distinct profile and prognostic impact of body composition changes in idiopathic pulmonary fibrosis and idiopathic pleuroparenchymal fibroelastosis	3
Suzuki et al. 2018	Skeletal muscle index (SMI) status and survival in patients undergoing surgery for colorectal cancer (CRC): A longitudinal study	2
Abbass et al. 2020	The change in the psoas muscle index in neoadjuvant therapy is a predictive prognostic marker in locally advanced rectal cancer	2
Fukuoka et al. 2017	Clinical significance of measurement of skeletal muscle volume and serum nutritional markers in esophageal cancer patients	2
Ishida et al. 2018	Low skeletal muscle mass in stented esophageal cancer predicts poor survival: A retrospective observational study	0
Jarvinen et al. 2018	Loss of skeletal muscle mass in follow up in palliatively stented esophageal cancer patients predicts worse outcome	2
Jarvinen et al. 2018	Reduction of skeletal muscle index as a predictive factor in patients with urothelial carcinoma	2
Nagai et al. 2017	Does sarcopenia impact oncologic outcomes in patients undergoing adjuvant chemotherapy following radical cystectomy?	2
Peak et al. 2017	A decline in weight and attrition of muscle in colorectal cancer patients receiving chemotherapy with bevacizumab	0
Poterucha et al. 2012	Sarcopenia determines post-progression outcomes in advanced hepatocellular carcinoma after sorafenib failure	2
Cheng et al. 2019	Can serum IL-6 levels predict sarcopenia and poor outcome in relapsed/refractory gynecologic cancer patients?	2
Yoshikawa et al. 2019	Measurement of body composition in cancer patients using CT planning scan at the third lumbar vertebra	6
Muresan et al. 2019	Sarcopenia Evaluated Using the Skeletal Muscle Index Is a Significant Prognostic Factor for Metastatic Urothelial Carcinoma	6
Taguchi et al. 2016	Relationship between presarcopenia and event occurrence in patients with primary hepatocellular carcinoma	6
Takada et al. 2020	Clinical impact of skeletal muscle area in patients with non-small cell lung cancer treated with anti-PD-1 inhibitors	6
Takada et al. 2020	Prognostic Significance of Skeletal Muscle Loss During Early Postoperative Period in Elderly Patients with Esophageal Cancer	0

Takada et al. 2019	Significance of skeletal muscle mass in neoadjuvant chemotherapy for pancreatic cancer	2
Takahashi et al. 2017	Change in body composition following systemic chemotherapy in patients with testicular germ cell tumor	2
Takai et al. 2019	Prognostic Impact of Postoperative Skeletal Muscle Decrease in Non-Small Cell Lung Cancer	0
Takamori et al. 2020	Clinical Impact and Risk Factors for Skeletal Muscle Loss After Complete Resection of Early Non-small Cell Lung Cancer	0
Takamori et al. 2018	The impact of body composition on short-term outcomes of neoadjuvant chemotherapy with gemcitabine plus S-1 in patients with resectable pancreatic cancer	6
Takeda et al. 2021	The impact of cachexia and sarcopenia in elderly pancreatic cancer patients receiving palliative chemotherapy	6
Takeda et al. 2021	Preoperative skeletal muscle index and visceral-to-subcutaneous fat area ratio are associated with long-term outcomes of elderly gastric cancer patients after gastrectomy	6
Taki et al. 2021	Sarcopenia is associated with toxicity in patients undergoing neo-adjuvant chemotherapy for oesophago-gastric cancer	0
Tan et al. 2015	Sarcopenia in an overweight or obese patient is an adverse prognostic factor in pancreatic cancer	0
Tan et al. 2009	Body composition measurement using bioelectrical impedance analysis scale during the first three cycles of chemotherapy	2
Tan et al. 2021	Characterization of advanced pancreatic cancer patients showing a decrease of the skeletal muscle mass while receiving first-line chemotherapy	0
Tatematsu et al. 2017	Association of body composition measured by bioelectrical impedance analysis and hematologic adverse events in early-stage breast cancer patients receiving chemotherapy	2
Thanestada et al. 2020	Nutritional status, cachexia and survival in patients with advanced colorectal carcinoma. Different assessment criteria for nutritional status provide unequal results	6
Thoresen et al. 2013	Sarcopenia and sarcopenic obesity in pancreatic ductal adenocarcinoma (PDAC) patients undergoing surgery after neoadjuvant therapy (NAT): Clinical implications	2
Trestini et al. 2020	Sarcopenia and sarcopenic obesity in pancreatic ductal adenocarcinoma (PDAC) patients undergoing surgery after neoadjuvant therapy (NAT): Prevalence and clinical implications	0
Trestini et al. 2020	Clinical impact of sarcopenia and sarcopenic obesity in pancreatic ductal adenocarcinoma (PDAC) patients undergoing surgery after neoadjuvant therapy (NAT)	2
Trestini et al. 2020	Impact of sarcopenia in patients treated with chemoimmunotherapy for advanced NSCLC	2
Trudu et al. 2021		6

	Body composition and sarcopenia before and after surgery for oesophageal cancer	7
Smedh et al. 2020	LOSS OF SKELETAL MUSCLE MASS DURING NEO-ADJUVANT CHEMOTHERAPY AND THE RELATION TO SURVIVAL IN PATIENTS WITH OVARIAN CANCER; A PROSPECTIVE ANALYSIS OF THE OVHIPEC-1 COHORT	0
Ubachs et al. 2019	Loss of skeletal muscle mass during neoadjuvant chemotherapy and the relation to survival in patients with ovarian cancer: A prospective analysis of the OVHIPEC-1 cohort	0
Ubachs et al. 2020	Loss of skeletal muscle mass during neoadjuvant chemotherapy and the relation to survival in patients with ovarian cancer: A prospective analysis of the OVHIPEC-1 cohort	2
Ubachs et al. 2020	The Impact of Skeletal Muscle Mass Loss in Patients with Advanced Pancreatic Cancer during Folfirinox	0
Uemura et al. 2020	The Impact of Skeletal Muscle Mass Loss in Patients with Advanced Pancreatic Cancer during Folfirinox	0
Uemura et al. 2020	The impact of sarcopenia and decrease in skeletal muscle mass in patients with advanced pancreatic cancer during FOLFIRINOX therapy	0
Uemura et al. 2021	Is sarcopenia associated with increased toxicity of neoadjuvant/ adjuvant chemotherapy for breast cancer?	2
Ueno et al. 2018	Is sarcopenia associated with increased toxicity of neoadjuvant/adjuvant chemotherapy for breast cancer?	0
Ueno et al. 2018	Sarcopenia and post-hospital outcomes in older adults: A longitudinal study	3
Ulises Perez-Zepeda et al. 2017	Incidence of sarcopenia in geriatric gastrointestinal cancer patients and its relationship with body mass index	2
Alivizatos et al. 2016	A natural history of cancer-associated cachexia	2
Baracos et al. 2012	Body composition analysis using computed tomography image in patients with advanced lung cancer	2
Coats et al. 2013	Changes in body composition during chemotherapy in women with breast cancer treated in the Netherlands	2
Van Den Berg et al. 2015	Cancer Cachexia: Identification by Clinical Assessment versus International Consensus Criteria in Patients with Metastatic Colorectal Cancer	6
van der Werf et al. 2018	The influence of body composition on the systemic exposure of paclitaxel in esophageal cancer patients	7
van Doorn et al. 2021	The influence of body composition on the systemic exposure of paclitaxel in esophageal cancer patients	2

Van Doorn et al. 2017	Low Skeletal Muscle Density Is Associated with Early Death in Patients with Perihilar Cholangiocarcinoma Regardless of Subsequent Treatment	6
Van Vugt et al. 2019	Adipose tissue and muscle attenuation as novel biomarkers predicting mortality in patients with extremity sarcomas	6
Veld et al. 2016	The effect of everolimus on adipose tissue in patients with metastatic breast cancer	2
Villaorduna et al. 2020	Prevalence and prognostic effect of sarcopenia in breast cancer survivors: the HEAL Study	6
Villaseñor et al. 2012	Impact of sarcopenia in patients with metastatic melanoma treated with immunotherapy	2
Vitale et al. 2019	Early changes in skeletal muscle as a strong prognostic biomarker in patients with metastatic renal cell carcinoma	2
w. Gu et al. 2016	Can total psoas area predict toxicity after stereotactic body radiation therapy in borderline resectable and locally advanced pancreatic cancers?	2
W. Jin et al. 2017	Sarcopenia Is Associated with Reduced Overall and Disease-Free Survival in Patients Undergoing Esophagectomy for Locally Advanced Esophageal Cancer	2
Wakefield et al. 2020	Sarcopenia Is Associated with Reduced Overall and Disease-Free Survival in Patients Undergoing Esophagectomy for Locally Advanced Esophageal Cancer	0
Wakefield et al. 2020	Loss of muscle mass in the end of life in patients with advanced cancer	0
Wallengren et al. 2015	Association of BMI, body composition and outcomes in Chinese patients with metastatic renal cell carcinoma treated with immunotherapy: A retrospective, multicohort analysis	2
Wang et al. 2021		6
Wang et al. 2021	Assessment of sarcopenia as a predictor of poor overall survival for advanced non-small-cell lung cancer patients receiving salvage anti-PD-1 immunotherapy	0
Wang et al. 2021	Diet and Exercise Are not Associated with Skeletal Muscle Mass and Sarcopenia in Patients with Bladder Cancer	6
Wang et al. 2019	Association of sarcopenia with dose-limiting toxicties and survival in oesophageal adenocarcinoma treated with neoadjuvant chemotherapy	2
Weaver et al. 2018	Determining the prevalence and severity of cancer cachexia in advanced non-small cell lung cancer and its relationship with chemotherapy outcomes	7
White et al. 2020	Determining the prevalence and severity of cancer cachexia in advanced non-small cell lung cancer and its relationship with chemotherapy outcomes	6
White et al. 2020	Pectoralis muscle wasting during chemotherapy	2
Wiederin et al. 2020	Hematological Toxicity in Low Muscle Mass Breast Cancer Patient Underwent Chemotherapy: A Preliminary Study	2

Wijovi et al. 2019	Early Loss of Fat Mass During Chemoradiotherapy Predicts Overall Survival in Locally Advanced Squamous Cell Carcinoma of the Lung, but Not in Locally Advanced Squamous Cell Carcinoma of the Head and Neck	6
Willemsen et al. 2020	Assessment of Sarcopenia Measures, Survival, and Disability in Older Adults Before and After Diagnosis With Cancer	0
Williams et al. 2020	Patient-reported and objectively measured physical function in older breast cancer survivors and cancer-free controls	6
Winters-Stone et al. 2019	Impact of sarcopenia on treatment tolerance in United States veterans with diffuse large B-cell lymphoma treated with CHOP-based chemotherapy	6
Xiao et al. 2016	The impact of body composition parameters on severe toxicities in patients with locoregionally advanced nasopharyngeal carcinoma undergoing neoadjuvant chemotherapy	6
Xing et al. 2021	The impact of body composition parameters on severe toxicities in patients with locoregionally advanced nasopharyngeal carcinoma undergoing neoadjuvant chemotherapy	6
Xing et al. 2021	Impact of skeletal muscle loss after adjuvant chemoradiotherapy in patients with gastric cancer	2
Gao et al. 2016	The influence of perioperative decreasing total psoas area on prognosis of esophagieal cancer patients	2
Hirata et al. 2016	Predictive value of pancreatic dose-volume metrics on sarcopenia rate in gastric cancer patients treated with adjuvant chemoradiotherapy	7
Li et al. 2018	Sarcopenia in patients with testicular cancer undergoing chemotherapy: Prognostic impact of psoas major muscle loss	2
Mitsui et al. 2018	Low skeletal muscle mass before salvage-line chemotherapy is a poor prognostic factor in patients with refractory metastatic colorectal cancer	6
Miyamoto et al. 2018	Prognostic significance of CT-determined sarcopenia in patients with advanced gastric cancer treated with chemotherapy	6
Y.s , Kim et al. 2018	P2.16-27 Longitudinal Skeletal Muscle Changes in Patients with Advanced Squamous Cell Lung Cancer	2
Y.s , Kim et al. 2019	Depletion of pre-transplant skeletal muscle is a significant poor prognostic factor in allogeneic hematopoietic cell transplantation	2
Shibasaki et al. 2019	The impact of muscle mass loss after esophagectomy for esophageal cancer on prognosis	2
Shimoda et al. 2019	Surgical approach and postoperative changes in body weight, skeletal muscle and adipose tissue mass in early gastric cancer patients	2

Yokota et al. 2017	Muscle volume loss after the induction of first-line chemotherapy as a novel prognostic factor in metastatic colorectal cancer patients	2
Yoshikawa et al. 2015	Skeletal muscle depletion during chemoradiotherapy and radiomics features as prognostic factors of toxicity and outcome in patients with gastric cancer	2
Yuan et al. 2018	Early cancer cachexia phenotype predicts survival of advanced urothelial cancer patients treated with pembrolizumab	6
Yamamoto et al. 2021	Skeletal muscle loss after total gastrectomy, exacerbated by adjuvant chemotherapy	0
Yamaoka et al. 2015	Quantifying skeletal muscle wasting during chemoradiotherapy with Jacobian calculations for the prediction of survival and toxicity in patients with gastric cancer	7
Yang et al. 2020	Correlation between skeletal muscle loss and clinical outcomes after chemotherapy in patients with advanced lung cancer	2
Yao et al. 2018	Decreased total psoas muscle area after neoadjuvant therapy is a predictor of increased mortality in patients undergoing oesophageal cancer resection	0
Yassaie et al. 2019	Role of the Appendicular Skeletal Muscle Index for Predicting the Recurrence-Free Survival of Head and Neck Cancer	7
Yeh et al. 2021	Assessment of sarcopenia and changes in body composition after neoadjuvant chemotherapy and associations with clinical outcomes in oesophageal cancer	6
Yip et al. 2014	Skeletal Muscle Changes Assessed by Preoperative Computed Tomography Images Can Predict the Long-Term Prognosis of Stage III Colorectal Cancer	7
Yokoi et al. 2021	Prognostic Impact of Sarcopenia and Skeletal Muscle Loss During Neoadjuvant Chemoradiotherapy in Esophageal Cancer	7
Yoon et al. 2020	Sarcopenia as a Predictor of Survival Among Patients With Organ Metastatic Cervical Cancer	6
Yoshikawa et al. 2020	Psoas muscle volume as a predictor of peripheral neurotoxicity induced by primary chemotherapy in ovarian cancers	6
Yoshikawa et al. 2017	Psoas muscle volume as a predictor of peripheral neurotoxicity induced by primary chemotherapy in ovarian cancers	6
Yoshikawa et al. 2017	An exploratory study of body composition as a predictor of dose-limiting toxicity in metastatic pancreatic cancer treated with gemcitabine plus nab-paclitaxel	0
Youn et al. 2021	An exploratory study of body composition as a predictor of dose-limiting toxicity in metastatic pancreatic cancer treated with gemcitabine plus nab-paclitaxel	6
Youn et al. 2021	Impact of body composition on outcomes from anti-PD1 +/- anti-CTLA-4 treatment in melanoma	6

Young et al. 2020	Effect of baseline sarcopenia on adjuvant treatment for D2 dissected gastric cancer: Analysis of the ARTIST phase III trial	6
Yu et al. 2020	Prognostic Importance of Sarcopenia in Patients with Clear Cell Carcinoma	0
Yuki et al. 2017	Sarcopenia as a significant predictive factor of neutropenia and overall survival in urothelial carcinoma patients underwent gemcitabine and cisplatin or carboplatin	6
Yumioka et al. 2020	Imaging-Based Subtypes of Pancreatic Ductal Adenocarcinoma Exhibit Differential Growth and Metabolic Patterns in the Pre-Diagnostic Period: Implications for Early Detection	6
Zaid et al. 2020	Sarcopenia Predicts Overall Survival in Patients with Lung, Breast, Prostate, or Myeloma Spine Metastases Undergoing Stereotactic Body Radiation Therapy (SBRT), Independent of Histology	7
Zakaria et al. 2020	Application of morphometrics as a predictor for survival in female patients with breast cancer spinal metastasis: a retrospective cohort study	6
Zakaria et al. 2018	Application of Morphometrics as a Predictor for Survival in Patients with Prostate Cancer Metastasis to the Spine	6
Zakaria et al. 2018	Refractory Nasopharyngeal Carcinoma: Positron Emission Tomography Combined with Computed Tomography-Guided I-125 Seed Implantation Therapy after Repeated Traditional Radiochemotherapy	6
Zhang et al. 2013	Incidence and risk factor analysis for sarcopenia in patients with cancer	6
Zhang et al. 2016	Skeletal-muscle index predicts survival after percutaneous transhepatic biliary drainage for obstructive jaundice due to perihilar cholangiocarcinoma	6
Zhang et al. 2020	Marked loss of adipose tissue during neoadjuvant therapy as a predictor for poor prognosis in patients with gastric cancer: A retrospective cohort study	7
Zhang et al. 2021	Impact of body composition on clinical outcomes in people with gastric cancer undergoing radical gastrectomy after neoadjuvant treatment	0
Zhang et al. 2021	Impact of body composition on clinical outcomes in people with gastric cancer undergoing radical gastrectomy after neoadjuvant treatment	0
Zhang et al. 2021	Novel Nutrition-Based Nomograms to Assess the Outcomes of Lung Cancer Patients Treated With Anlotinib or Apatinib	0
Zheng et al. 2021	Low Subcutaneous Adiposity and Mortality in Esophageal Cancer	6
Zhou et al. 2021	Development and validation of a clinically applicable score to classify cachexia stages in advanced cancer patients	6

Zhou et al. 2018	EWGSOP2 versus EWGSOP1 for sarcopenia to predict prognosis in patients with gastric cancer after radical gastrectomy: Analysis from a large-scale prospective study	6
Zhuang et al. 2020	Sarcopenia in Metastatic Renal Cell Carcinoma Patients Treated with Cabozantinib	7
Buchler 2020	Loss of adipose tissue or skeletal muscle during first-line gemcitabine/nab-paclitaxel therapy is associated with worse survival after second-line therapy of advanced pancreatic cancer	6
Miki 2022	Skeletal muscle mass loss and dose-limiting toxicities in metastatic colorectal cancer patients	6
Kurk 2019	Early skeletal muscle loss during target therapy is a prognostic biomarker in metastatic renal cell carcinoma patients	7
Gu 2017	Posttherapeutic skeletal muscle mass recovery predicts favorable prognosis in patients with advanced urothelial carcinoma receiving first-line platinum-based chemotherapy	7
Fukushima 2018	Loss of skeletal muscle during systemic chemotherapy is prognostic of poor survival in patients with foregut cancer	7
Daly 2018	Comparison of skeletal muscle mass loss in patients with metastatic colorectal cancer treated with regorafenib or TAS-102	6
Hacioglu 2019	Change in Psoas Muscle Volume as a Predictor of Outcomes in Patients Treated with Chemotherapy and Radical Cystectomy for Muscle-Invasive Bladder Cancer	6
Zargar 2017	Changes in skeletal muscle function during chemotherapy and related factors in patients with acute leukemia	6
Terao 2024	Skeletal muscle mass during chemotherapy for haematological malignancies: a retrospective study	6
Takahashi 2024	Prognostic value of computed tomography associated body composition measurement changes in metastatic colorectal cancer patients	5
Sevgilioglu 2023	Changes in body composition and muscle attenuation during taxane-based chemotherapy in patients with metastatic breast cancer	6
Rier 2018	The impact of lenvatinib on sarcopenia in patients with advanced unresectable hepatocellular carcinoma	7
Praktiknjo 2024	CT-based body composition in diffuse large B cell lymphoma patients: changes after treatment and association with survival	7
Pirosa 2023	Developing sarcopenia during neoadjuvant therapy is associated with worse survival in esophageal adenocarcinoma patients	7

Pierce 2024	Association of Sarcopenia with and Efficacy of Anti-PD-1/PD-L1 Therapy in Non-Small-Cell Lung Cancer	6
Nishioka 2019	Survival impact and risk factors of skeletal muscle loss during first-line EGFR-TKIs therapy in advanced lung adenocarcinoma patients	7
Nie 2025	Skeletal muscle depletion during chemotherapy has a large impact on physical function in elderly Japanese patients with advanced non-small-cell lung cancer	6
Naito 2017	Skeletal Muscle Mass Reduction Velocity as a Simple Prognostic Indicator for Patients with Metastatic Urothelial Carcinoma Receiving Second-Line Chemotherapy	6
Nagai 2019	Measuring body composition using the bioelectrical impedance method can predict the outcomes of gemcitabine-based chemotherapy in patients with pancreatobiliary tract cancer	6
Muramatso 2015	Negative Impact of Skeletal Muscle Loss after Systemic Chemotherapy in Patients with Unresectable Colorectal Cancer	6
Miyamoto 2015	Changes in Skeletal Muscle Volume During Preoperative Chemotherapy Affect the Outcome of Pancreatic Cancer.	6
Matsumoto 2025	The application of QCT in the prognostic assessment of mCRC undergoing first-line treatment based on bevacizumab	7
Liu 2024	Muscle and Adipose Wasting despite Disease Control: Unaddressed Side Effects of Palliative Chemotherapy for Pancreatic Cancer	6
Klassen 2023	Doxorubicin combined with ifosfamide for sarcoma induces muscle atrophy and sleep disruption	6
Kinoshita 2021	Prognostic impact of cancer cachexia in patients with advanced non-small cell lung cancer	6
Kimura 2015	Three cachexia phenotypes and the impact of fat-only loss on survival in FOLFIRINOX therapy for pancreatic cancer	6
Kays 2018	Depletion of psoas muscle mass after systemic chemotherapy is associated with poor prognosis in patients with unresectable pancreatic cancer	6
Iwai 2021	Lenvatinib or Sorafenib Treatment Causing a Decrease in Skeletal Muscle Mass, an Independent Prognostic Factor in Hepatocellular Carcinoma: A Survival Analysis Using Time-Varying Covariates	7
Imai 2023	Impact of CT-based body composition parameters at baseline, their early changes and response in metastatic cancer patients treated with immune checkpoint inhibitors	6

Crombé 2020	Skeletal muscle depletion predicts survival of patients with advanced biliary tract cancer undergoing palliative chemotherapy	6
Cho 2017	Skeletal muscle depletion predicts the prognosis of patients with advanced pancreatic cancer undergoing palliative chemotherapy, independent of body mass index	6
Choi 2015	The Analysis of Muscle Volume Measured by Bioelectrical Impedance in Patients with Hepatocellular Carcinoma Treated with First-Line Atezolizumab plus Bevacizumab Combination Therapy or First-Line Lenvatinib	7
Chihiro 2023	Frequency of weight and body composition increases in advanced non-small cell lung cancer patients during first line therapy	6
Bonomi 2024	The impact of changes in radiographic sarcopenia on overall survival in older adults undergoing different treatment pathways for pancreatic cancer	6
Benjamin 2018	The Impact of Multidisciplinary Weight Management on Body Weight and Body Mass Composition in Women with Breast Cancer Post-Adjuvant Chemotherapy: A Retrospective Chart Review.	4
Nguyen 2022	Clinical impact of the prognostic nutritional index and skeletal muscle index for the incompletion of adjuvant chemotherapy for pancreatic cancer	7
Maehira 2025	Muscle loss during cancer therapy is associated with poor outcomes in advanced ovarian cancer.	7
Polen-De 2023	Loss of Skeletal Muscle Mass During NeoadjuvantChemotherapy for Pancreatic Cancer Is Related to the Continuation of S-1 Adjuvant Chemotherapy After Pancreatectomy	2
Kawahara 2024	Weight Loss During Neoadjuvant Therapy Is Associated With Poor Response Among the Patients With Gastrointestinal Cancer: A Propensity Score Matching Analysis	7
Bu 2023	Early skeletal muscle loss in adolescent and young adult cancer patients treated with anthracycline chemotherapy	3
Wooten 2023	Dynamic Anthropometrics: Sarcopenia and Survival in Pancreatic Cancer	7
Yee 2023	Investigating sarcopenia, physical activity, and inflammation biomarkers in newly diagnosed oral cancer patients during curative treatment: A prospective longitudinal study	7
Huang 2023	The impact of preoperative skeletal muscle loss on the completion of S-1 adjuvant chemotherapy for gastric cancer	7
Nakabayashi 2024	Prognostic Impact of the Loss of Skeletal Muscle Mass During Neoadjuvant Chemotherapy on Older Patients with Esophageal Cancer.	7

Harada 2022	Association of Perioperative Skeletal Muscle Index Change With Outcome in Colorectal Cancer Patients	7
Li 2024	Body composition parameters were associated with response to abiraterone acetate and prognosis in patients with metastatic castration-resistant prostate cancer.	7
Ke 2023	Nomogram of Combining CT-Based Body Composition Analyses and Prognostic Inflammation Score: Prediction of Survival in Advanced Epithelial Ovarian Cancer Patients.	7
Wang 2022	Usefulness of skeletal muscle measurement by computed tomography in patients with esophageal cancer: changes in skeletal muscle mass due to neoadjuvant therapy and the effect on the prognosis	7
Yamamoto 2023	Body composition in recurrent prostate cancer and the role of steroidogenic genotype	7
Venkatesh 2024	Cholinesterase as a predictor of skeletal muscle loss after gastrectomy for gastric cancer	7
Takano 2024	The ratio of skeletal muscle mass to body mass index combined with inflammatory immune markers to stratify survival of pancreatic cancer after pancreatoduodenectomy	6
Jin 2024	The impact of sarcopenia on overall survival in patients with pan-RAS wild-type colorectal liver metastasis receiving hepatectomy.	7
Yang 2023	Association of malignant ascites with systemic inflammation and muscle loss after treatment in advanced-stage ovarian cancer	7
Weng 2023	Increased adipose tissue is associated with improved overall survival, independent of skeletal muscle mass in non-small cell lung cancer	7
Tao 2023	Impact of body composition, grip strength, and physical performance on clinical outcomes for locally advanced gastric cancer during neoadjuvant chemotherapy: A prospective cohort study	7
Zhou 2024	Effect of Multimodal Prehabilitation on Muscle Mass in Rectal Cancer Patients Receiving Neoadjuvant Treatment	7
Yadav 2024	Evaluation of sarcopenia-associated survival in breast cancer with computed tomography-based pectoral muscle area measurements	7
Kuzan 2024	Changes in skeletal muscle and adipose tissue during cytotoxic chemotherapy for testicular germ cell carcinoma and associations with adverse events.	7
Phoung 2022	Skeletal muscle mass recovery after oesophagectomy and neoadjuvant chemotherapy in oesophageal cancer: Retrospective cohort study	6
Harada 2023	CT-based skeletal muscle loss predicts long term prognosis in patients with distal cholangiocarcinoma undergone pancreaticoduodenectomy	7

Qu 2025	Prognostic value of body composition measures in breast cancer patients treated with chemotherapy	5
Kang 2024	Increment of Skeletal Muscle Mass Predicts Survival Benefit for Hepatocellular Carcinoma Treated with Transarterial Chemoembolization Combining Molecular Targeted Agents and Immune Checkpoint Inhibitors	7
Chen 2025	Association between quantitative CT body composition analysis and prognosis in cetuximab-based first-line treatment for advanced colorectal cancer patients	6
Dang 2024	Body composition and inflammation impact in non-small-cell lung cancer patients treated by first-line immunotherapy.	6
Baldessari 2021	Sarcopenia and high NLR are associated with the development of hyperprogressive disease after second-line pembrolizumab in patients with non-small-cell lung cancer	6
Petrova 2020	Cachexia - sarcopenia as a determinant of disease control rate and survival in non-small lung cancer patients receiving immune-checkpoint inhibitors	6
Roch 2020	Skeletal muscle adaptations in patients with lung cancer: Longitudinal observations from the whole body to cellular level	6
Snoke 2023	Physical function, nutritional status, and quality of life before and after chemotherapy in patients with malignant lymphoma.	4
Jonbo 2023	Early skeletal muscle deconditioning and reduced exercise capacity during (neo)adjuvant chemotherapy in patients with breast cancer.	5
Mallard 2023	Longitudinal Body Composition Identifies Hepatocellular Carcinoma With Cachexia Following Combined Immunotherapy and Target Therapy (CHANCE2213)	5
Jin 2024	Association of computed tomography-based body composition with survival in metastatic renal cancer patient received immunotherapy: a multicenter, retrospective study.	6
Wang 2023	CT-derived skeletal muscle change before immunotherapy predicts survival of advanced gastric cancer: associations with inflammatory markers and liver lipid metabolism	6
Hayano 2024	Early skeletal muscle mass decline is a prognostic factor in patients receiving gemcitabine plus nab-paclitaxel for unresectable pancreatic cancer: a retrospective observational study.	6
Suzuki 2023	Poor Muscle Status, Dietary Protein Intake, Exercise Levels, Quality of Life and Physical Function in Women with Metastatic Breast Cancer at Chemotherapy Commencement and during Follow-Up.	6
Parkinson 2023	Impact of losing adipose tissue on outcomes from PD-1/PD-L1 inhibitor monotherapy in non-small cell lung cancer.	6

Nishioka 2022	Effect of changes in body composition and sociodemographic factors on colon cancer survival	2
Vilaplana 2025	Influence of the skeletal muscle index on pharmacokinetics and toxicity of fluorouracil	6
Schmulenson 2023	The Impact of Pre-Chemotherapy Body Composition and Immunonutritional Markers on Chemotherapy Adherence in Stage III Colorectal Cancer Patients	6
Lee 2023	Is Computed-Tomography-Based Body Composition a Reliable Predictor of Chemotherapy-Related Toxicity in Pancreatic Cancer Patients?	6
Cefali 2023	Impact of muscle mass loss on outcomes in advanced or metastatic gastric cancer patients receiving a second-line treatment	6
Zurlo 2024	Longitudinal Changes in Skeletal Muscle Metabolism, Oxygen Uptake, and Myosteatosis During Cardiotoxic Treatment for Early-Stage Breast Cancer.	6
Kirkham 2022	Dynamic Changes in Body Composition and Protein Intake in Epithelial Ovarian Cancer Patients Undergoing Chemotherapy: A Preliminary Study	6
Manikan 2024	CT-derived body composition measurements as predictors for neoadjuvant treatment tolerance and survival in gastroesophageal adenocarcinoma.	6
DeFreitas 2023	Impact of Skeletal Muscle Depletion on Patients with Myelodysplastic Syndrome Treated with Azacitidine	6
Takada 2024	Subcutaneous Fat Abundance and Density Are Associated with an Enhanced Response to Immunotherapy in Metastatic Melanoma: A Retrospective Cohort Study.	5
Mangoni 2023	[Observation of Nutritional Status Changes in Patients with Acute Leukemia During Chemotherapy].	5
Zong 2022	Evaluation of the cachexia index using a bioelectrical impedance analysis in elderly patients with non-Hodgkin's lymphoma: A single-center prospective study.	6
Okubo 2024	Dose optimization of pancreatic enzyme replacement therapy is essential to mitigate muscle loss in patients with advanced pancreatic cancer and exocrine pancreatic insufficiency.	7
Klassen 2024	Hematologic toxicities, sarcopenia, and body composition change in breast cancer patients undergoing neoadjuvant chemotherapy	5
Jang 2023	Diagnosis of Sarcopenia and Myosteatosis by Computed Tomography in Patients with Esophagogastric and Pancreatic Cancer	5
Sales-Balaguer 2024	Changes in Skeletal Muscle Volume During Preoperative Chemotherapy Affect the Outcome of Pancreatic Cancer	5

Matsumoto 2024	Use of automated assessment for determining associations of low muscle mass and muscle loss with overall survival in patients with colorectal cancer - A validation study	6
Smit 2024	Predicting the Risks of Aggressive-Intent Chemotherapy Toxicity in Older Patients With Lymphoma: A Prospective Observational Pilot Study.	5
Dhir 2022	Toxicity of induction chemotherapy in head and neck cancer: The central role of skeletal muscle mass.	6
Lere-Chevaleyre 2022	The association between geriatric assessment, muscle measures, and treatment-related toxicity in older adults with cancer: An Israeli prospective study.	6
Schchar 2022	Creatinine-to-cystatin C ratio and body composition predict response to PD-1 inhibitors-based combination treatment in metastatic gastric cancer	6
Ji 2024	Low Skeletal Muscle as a Risk Factor for Worse Survival in Nonmetastatic Renal Cell Carcinoma with Venous Tumor Thrombus	6
Schmeusser 2023	Influence of adipose tissue distribution, sarcopenia, and nutritional status on clinical outcomes after CD19 CAR T-cell therapy.	6
Rejeski 2023	Chemotherapy dose per kilogram lean body mass increased dose-limiting toxicity event in male head and neck cancer with taxane and platinum-based induction therapy.	6
Hung 2022	Implication of Skeletal Muscle Loss in the Prognosis of Patients with Pancreatic Ductal Adenocarcinoma Receiving Chemotherapy	6
Ishizaki 2023	Prognostic Value of Skeletal Muscle Loss in Patients with Hepatocellular Carcinoma Treated with Hepatic Arterial Infusion Chemotherapy	6
Oura 2023	Sarcopenia in Men With Bone-Predominant Metastatic Castration-Resistant Prostate Cancer Undergoing Ra-223 Therapy	7
Khan 2023	Dynamic changes in body composition during XELOX/SOX chemotherapy in patients with gastric cancer	4
Li 2024	Efficacy and safety of anamorelin in patients with cancer cachexia: Post-hoc subgroup analyses of a placebo-controlled study.	6
Takayama 2023	Quantitative analysis of the effect of docetaxel-induced edema on quality of life in patients with breast cancer and related factors: a prospective cohort study	6
Izawa 2024	Body composition parameters for predicting the efficacy of neoadjuvant chemotherapy with immunotherapy for gastric cancer.	4

Lin 2022	Rituximab, cyclophosphamide, doxorubicin, vincristine, and prednisolone (R-CHOP) therapy decreases lean body mass and appendicular skeletal muscle mass index even until one year after the final treatment in patients with B-cell non-Hodgkin lymphoma	6
Nakao 2024	CROSS Versus FLOT Regimens in Esophageal and Esophagogastric Junction Adenocarcinoma: A Propensity-Matched Comparison.	6
Donlon 2022	Clinical Significance of Increased Skeletal Muscle Mass During Nab-Paclitaxel Plus Gemcitabine Treatment in Patients With Advanced Pancreatic Cancer	6
Lede 2024	Sarcopenic obesity predicts short- and long-term outcomes after neoadjuvant chemotherapy and surgery for gastric cancer	4
Duan 2024	Gonadotropic Axis, Bone Mass, and Sarcopenia Assessment After Autologous Hematopoietic Stem Cell Transplantation for Lymphoma.	7
Leal 2024	The relationship between prognosis and temporal muscle thickness in 102 patients with glioblastoma	7
Tang 2024	Sarcopenia and myosteatosis are associated with survival in patients receiving immunotherapy for advanced hepatocellular carcinoma	6

References

1 Griffin, O. M., Duggan, S. N., Ryan, R., McDermott, R., Geoghegan, J. & Conlon, K. C. Characterising the impact of body composition change during neoadjuvant chemotherapy for pancreatic cancer. *Pancreatology* **19**, 850-857 (2019). https://doi.org:10.1016/j.pan.2019.07.039

2 Lee, M. W. *et al.* Prognostic value of initial and longitudinal changes in body composition in metastatic pancreatic cancer. *J Cachexia Sarcopenia Muscle* **15**, 735-745 (2024). https://doi.org:10.1002/jcsm.13437

3 Rollins, K. E. *et al.* The impact of sarcopenia and myosteatosis on outcomes of unresectable pancreatic cancer or distal cholangiocarcinoma. *Clin Nutr* **35**, 1103-1109 (2016). https://doi.org:10.1016/j.clnu.2015.08.005

4 Davis, M. P. *et al.* Are Muscle and Fat Loss Predictive of Clinical Events in Pancreatic Cancer? The Importance of Precision Metrics. *J Pain Symptom Manage* **69**, 141-151 (2025). https://doi.org:10.1016/j.jpainsymman.2024.10.004

5 Uemura, S. *et al.* The impact of sarcopenia and decrease in skeletal muscle mass in patients with advanced pancreatic cancer during FOLFIRINOX therapy. *Br J Nutr*, 1-8 (2020). https://doi.org:10.1017/s0007114520003463

6 Lee, H. *et al.* Skeletal Muscle Mass Predicts Poor Prognosis in Patients with Advanced Pancreatic Cancer Undergoing Second-Line FOLFIRINOX Chemotherapy. *Nutrition and Cancer* (2019).

7 Takai, Y. *et al.* Body composition changes following chemotherapy for testicular germ cell tumor: obesity is the long-term problem. *Asian journal of andrology.* **03** (2021).

8 Rimar, K. J., Glaser, A. P., Kundu, S., Schaeffer, E. M., Meeks, J. & Psutka, S. P. Changes in Lean Muscle Mass Associated with Neoadjuvant Platinum-Based Chemotherapy in Patients with Muscle Invasive Bladder Cancer. *Bladder Cancer* **4**, 411-418 (2018). https://doi.org:10.3233/BLC-180188

9 Buxton, C. *et al.* A Multicenter Evaluation of Treatment-associated Changes in Body Composition in Men With Germ Cell Tumors of the Testis: Implications for Adverse Events and Complications. *Urology* **192**, 74-82 (2024). https://doi.org:10.1016/j.urology.2024.06.030

10 Semerad, O., Buchler, T., Vejmelka, J., Rozsypalova, A., Tomesova, J. & Kohout, P. Body composition changes during and after curative chemotherapy in patients with testicular cancer. *Biomedical Papers-Olomouc* https://doi.org:10.5507/bp.2020.058

11 Mitsui, Y. *et al.* Loss of psoas major muscle volume during systemic chemotherapy is related to worse prognosis in testicular cancer. *Japanese Journal of Clinical Oncology* **49**, 183-189 (2019). https://doi.org:10.1093/jjco/hyy166

12 MacDonald, L. *et al.* An in-depth analysis on the effects of body composition in patients receiving neoadjuvant chemotherapy for urothelial cell carcinoma. *Can Urol Assoc J* **18**, 180-184 (2024). https://doi.org:10.5489/cuaj.8542

13 Miyake, M. *et al.* Clinical utility of bioelectrical impedance analysis in patients with locoregional muscle invasive or metastatic urothelial carcinoma: a subanalysis of changes in body composition during neoadjuvant systemic chemotherapy. *Support Care Cancer* **26**, 1077-1086 (2018). https://doi.org:10.1007/s00520-017-3924-0

14 Kazemi-Bajestani, S. *et al.* Rapid atrophy of cardiac left ventricular mass in patients with non-small cell carcinoma of the lung. *Journal of Cachexia* (2019).

15 Khan, A. *et al.* Association of Computed Tomography Measures of Muscle and Adipose Tissue and Progressive Changes throughout Treatment with Clinical Endpoints in Patients with Advanced Lung Cancer Treated with Immune Checkpoint Inhibitors. *Cancers (Basel)* **15** (2023). https://doi.org:10.3390/cancers15051382

16 Nattenmüller, J. *et al.* Prognostic Impact of CT-Quantified Muscle and Fat Distribution before and after First-Line-Chemotherapy in Lung Cancer Patients. *PLoS ONE* **12**, e0169136 (2017). https://doi.org:10.1371/journal.pone.0169136
17 Kakinuma, K. *et al.* Differences in skeletal muscle loss caused by cytotoxic chemotherapy and molecular targeted therapy in patients with advanced non-small cell lung cancer. *Thoracic Cancer* **9(1)**, 99-104 (2018).
18 Kidd, A. C. *et al.* The prevalence and prognostic significance of Sarcopenia and Adipopenia in Pleural Mesothelioma. *Cancer Treat Res Commun* **42**, 100856 (2024). https://doi.org:10.1016/j.ctarc.2024.100856
19 Goncalves, M. D. *et al.* Imaging skeletal muscle volume, density, and FDG uptake before and after induction therapy for non-small cell lung cancer. *Clinical Radiology* **73**, 505.e501-505.e508 (2018). https://doi.org:10.1016/j.crad.2017.12.004
20 Juez, L. D. *et al.* Impact of Neoadjuvant Treatment on Body Composition in Patients with Locally Advanced Gastric Cancer. *Cancers (Basel)* **16** (2024). https://doi.org:10.3390/cancers16132408
21 Palmela, C. *et al.* Body Composition as a Prognostic Factor of Neoadjuvant Chemotherapy Toxicity and Outcome in Patients with Locally Advanced Gastric Cancer. *J Gastric Cancer* **17**, 74-87 (2017). https://doi.org:10.5230/jgc.2017.17.e8
22 Hacker, U. T. *et al.* Modified Glasgow prognostic score (mGPS) is correlated with sarcopenia and dominates the prognostic role of baseline body composition parameters in advanced gastric and esophagogastric junction cancer patients undergoing first-line treatment from the phase III EXPAND trial. *Ann Oncol* **33**, 685-692 (2022). https://doi.org:10.1016/j.annonc.2022.03.274
23 Miyata, H. *et al.* Clinical Assessment of Sarcopenia and Changes in Body Composition During Neoadjuvant Chemotherapy for Esophageal Cancer. *Anticancer Res* **37**, 3053-3059 (2017). https://doi.org:10.21873/anticanres.11660
24 Li, W. *et al.* Body Composition Decrease and Impact on Clinical Outcome in Gastric Cancer Patients Undergoing Radical Gastrectomy After Neoadjuvant Treatment. *Nutrition and cancer* **77**, 1-12 (2024). https://doi.org:10.1080/01635581.2024.2418622
25 den Boer, R. B. *et al.* Impact on postoperative complications of changes in skeletal muscle mass during neoadjuvant chemotherapy for gastro-oesophageal cancer. *Bjs Open* **4**, 847-854 (2020). https://doi.org:10.1002/bjs5.50331
26 Awad, S. *et al.* Marked changes in body composition following neoadjuvant chemotherapy for oesophagogastric cancer. *Clin Nutr* **31**, 74-77 (2012). https://doi.org:10.1016/j.clnu.2011.08.008
27 Ishida, T. *et al.* Impact of measurement of skeletal muscle mass on clinical outcomes in patients with esophageal cancer undergoing esophagectomy after neoadjuvant chemotherapy. *Surgery* **166**, 1041-1047 (2019). https://doi.org:10.1016/j.surg.2019.07.033
28 Matsuura, N. *et al.* Correlation between Skeletal Muscle Mass and Adverse Events of Neoadjuvant Chemotherapy in Patients with Gastric Cancer. *Oncology* **98**, 29-34 (2020). https://doi.org:10.1159/000502613
29 Rinninella, E. *et al.* Body Composition Changes in Gastric Cancer Patients during Preoperative FLOT Therapy: Preliminary Results of an Italian Cohort Study. *Nutrients* **13** (2021). https://doi.org:10.3390/nu13030960
30 Onishi, S. *et al.* Prognostic impact of shift to low visceral fat mass after neoadjuvant chemotherapy in patients with esophageal cancer. *Cancer Rep (Hoboken)* **7**, e2084 (2024). https://doi.org:10.1002/cnr2.2084
31 Zhao, Y. *et al.* Impact of Body Composition on Clinical Outcomes in Patients with Esophageal Squamous Cell Carcinoma Receiving Neoadjuvant Immunotherapy Plus Chemotherapy. *Ann Ital Chir* **95**, 284-293 (2024). https://doi.org:10.62713/aic.3336
32 Sugiyama, K. *et al.* Baseline sarcopenia and skeletal muscle loss during chemotherapy affect survival outcomes in metastatic gastric cancer. *Anticancer Research* **38(10)**, 5859-5866 (2018).

33 Fujihata, S. *et al.* The impact of skeletal muscle wasting during neoadjuvant chemotherapy on postoperative anastomotic leakage in patients with esophageal cancer. *Esophagus* **18**, 258-266 (2021). https://doi.org:10.1007/s10388-020-00774-1
34 Park, S. E. *et al.* Loss of skeletal muscle mass during palliative chemotherapy is a poor prognostic factor in patients with advanced gastric cancer. *Sci Rep* **10**, 17683 (2020). https://doi.org:10.1038/s41598-020-74765-8
35 Horii, N. *et al.* Psoas muscle depletion during preoperative chemotherapy for advanced gastric cancer has a negative impact on long-term outcomes after gastrectomy. *Asia-Pacific Journal of Clinical Oncology* https://doi.org:10.1111/ajco.13514
36 Dijksterhuis, W. P. M. *et al.* Association between body composition, survival, and toxicity in advanced esophagogastric cancer patients receiving palliative chemotherapy. *J Cachexia Sarcopenia Muscle* **10**, 199-206 (2019). https://doi.org:10.1002/jcsm.12371
37 Harada, T. *et al.* Clinical mechanism of muscle mass loss during neoadjuvant chemotherapy in older patients with esophageal cancer: a prospective cohort study. *Dis Esophagus* **38** (2025). https://doi.org:10.1093/dote/doae096
38 van der Zanden, V. *et al.* Loss of skeletal muscle density during neoadjuvant chemotherapy in older women with advanced stage ovarian cancer is associated with postoperative complications. *European Journal of Surgical Oncology.* (2021).
39 Ubachs, J. *et al.* No influence of sarcopenia on survival of ovarian cancer patients in a prospective validation study. *Gynecol Oncol* **159**, 706-711 (2020). https://doi.org:10.1016/j.ygyno.2020.09.042
40 Yoshino, Y. *et al.* Extreme skeletal muscle loss during induction chemotherapy is an independent predictor of poor survival in advanced epithelial ovarian cancer patients. *J Obstet Gynaecol Res* (2020). https://doi.org:10.1111/jog.14516
41 Wood, N. *et al.* Association between CT-based body composition assessment and patient outcomes during neoadjuvant chemotherapy for epithelial ovarian cancer. *Gynecol Oncol* **169**, 55-63 (2023). https://doi.org:10.1016/j.ygyno.2022.11.024
42 Toama, W. *et al.* Impact of pectoralis muscle loss on cardiac outcome and survival in Cancer patients who received anthracycline based chemotherapy: retrospective study. *BMC Cancer* **22**, 763 (2022). https://doi.org:10.1186/s12885-022-09882-w
43 Roeland, E. J. *et al.* FIT: Functional and imaging testing for patients with metastatic cancer. *Supportive Care in Cancer* https://doi.org:10.1007/s00520-020-05730-4
44 Loosen, S. H. *et al.* Progressive Sarcopenia Correlates with Poor Response and Outcome to Immune Checkpoint Inhibitor Therapy. *Journal of Clinical Medicine* **10** (2021). https://doi.org:10.3390/jcm10071361
45 Okuno, M. *et al.* Loss of muscle mass during preoperative chemotherapy as a prognosticator for poor survival in patients with colorectal liver metastases. *Surgery (United States)* **165(2)**, 329-336 (2019).
46 Blauwhoff-Buskermolen, S. *et al.* Loss of Muscle Mass During Chemotherapy Is Predictive for Poor Survival of Patients With Metastatic Colorectal Cancer. *J. Clin. Oncol.* **34**, 1339-1344 (2016). https://doi.org:10.1200/JCO.2015.63.6043
47 Gallois, C. *et al.* Skeletal muscle loss under chemotherapy and its association with survival and systemic treatment toxicity in metastatic colorectal cancer: An AGEO prospective multicenter study. *Journal of Clinical Oncology. Conference* **38** (2020).
48 Nozawa, H., Emoto, S., Murono, K., Shuno, Y. & Ishihara, S. Change in skeletal muscle indexand its prognostic significance inconversion therapy for initiallyunresectable colorectal cancer. *Journal of Clinical Oncology* **39** (2021). https://doi.org:http://dx.doi.org/10.1200/JCO.2021.39.3_suppl.56
49 Palle, S. S. *et al.* Multi-frequency bioelectrical impedance analysis (BIA) compared to magnetic resonance imaging (MRI) for estimation of fat-free mass in colorectal cancer patients treated with chemotherapy. *Clinical Nutrition Espen* **16**, 8-15 (2016).

50 Karaca, M. *et al.* Sarcopenia's Role in Neoadjuvant Chemotherapy Outcomes for Locally Advanced Breast Cancer: A Retrospective Analysis. *Med Sci Monit* **30**, e945240 (2024). https://doi.org:10.12659/msm.945240

51 Zhang, Y. *et al.* Neoadjuvant therapy increases the risk of metabolic disorders and osteosarcopenia in patients with early breast cancer. *Jpn J Clin Oncol* **54**, 959-966 (2024). https://doi.org:10.1093/jjco/hyae070

52 Rossi, F. *et al.* Muscle mass loss in breast cancer patients of reproductive age (≤ 45 years) undergoing neoadjuvant chemotherapy. *Radiol Med* **128**, 49-57 (2023). https://doi.org:10.1007/s11547-022-01574-6

53 Amitani, M. *et al.* Skeletal muscle loss during neoadjuvant chemotherapy predicts poor prognosis in patients with breast cancer. *BMC Cancer* **22**, 327 (2022). https://doi.org:10.1186/s12885-022-09443-1

54 Mazzuca, F. *et al.* Lean body mass wasting and toxicity in early breast cancer patients receiving anthracyclines. *Oncotarget* **9**, 25714-25722 (2018). https://doi.org:10.18632/oncotarget.25394

55 Camilleri, G. M. *et al.* Prevalence and survival implications of malnutrition and sarcopenia in metastatic breast cancer: A longitudinal analysis. *Clin Nutr* **43**, 1710-1718 (2024). https://doi.org:10.1016/j.clnu.2024.06.014

56 Jung, G. H., Kim, J. H. & Chung, M. S. Changes in weight, body composition, and physical activity among patients with breast cancer under adjuvant chemotherapy. *Eur J Oncol Nurs* **44**, 101680 (2020). https://doi.org:10.1016/j.ejon.2019.101680

57 Campbell, K. L., Lane, K., Martin, A. D., Gelmon, K. A. & McKenzie, D. C. Resting energy expenditure and body mass changes in women during adjuvant chemotherapy for breast cancer. *Cancer nursing* **30(2)**, 95-100 (2007).

58 Jang, M. K., Park, S., Park, C., Doorenbos, A. Z., Go, J. & Kim, S. Does neoadjuvant chemotherapy regimen affect sarcopenia status in patients with breast cancer? *Breast* **66**, 1-7 (2022). https://doi.org:10.1016/j.breast.2022.08.009
